# Defect patterns on the curved surface of fish retinae suggest a mechanism of cone mosaic formation

**DOI:** 10.1371/journal.pcbi.1008437

**Published:** 2020-12-15

**Authors:** Hayden Nunley, Mikiko Nagashima, Kamirah Martin, Alcides Lorenzo Gonzalez, Sachihiro C. Suzuki, Declan A. Norton, Rachel O. L. Wong, Pamela A. Raymond, David K. Lubensky

**Affiliations:** 1 Biophysics Program, University of Michigan, Ann Arbor, Michigan, United States of America; 2 Department of Ophthalmology and Visual Sciences, University of Michigan, Ann Arbor, Michigan, United States of America; 3 Neuroscience Graduate Program, University of Michigan, Ann Arbor, Michigan, United States of America; 4 Department of Biological Structure, University of Washington, Seattle, Washington, United States of America; 5 Department of Physics, Trinity College Dublin, Dublin, Ireland; 6 Department of Physics, University of Michigan, Ann Arbor, Michigan, United States of America; 7 Department of Molecular, Cellular, and Developmental Biology, University of Michigan, Ann Arbor, Michigan, United States of America; Purdue University, UNITED STATES

## Abstract

The outer epithelial layer of zebrafish retinae contains a crystalline array of cone photoreceptors, called the cone mosaic. As this mosaic grows by mitotic addition of new photoreceptors at the rim of the hemispheric retina, topological defects, called “Y-Junctions”, form to maintain approximately constant cell spacing. The generation of topological defects due to growth on a curved surface is a distinct feature of the cone mosaic not seen in other well-studied biological patterns like the R8 photoreceptor array in the *Drosophila* compound eye. Since defects can provide insight into cell-cell interactions responsible for pattern formation, here we characterize the arrangement of cones in individual Y-Junction cores as well as the spatial distribution of Y-junctions across entire retinae. We find that for individual Y-junctions, the distribution of cones near the core corresponds closely to structures observed in physical crystals. In addition, Y-Junctions are organized into lines, called grain boundaries, from the retinal center to the periphery. In physical crystals, regardless of the initial distribution of defects, defects can coalesce into grain boundaries via the mobility of individual particles. By imaging in live fish, we demonstrate that grain boundaries in the cone mosaic instead appear during initial mosaic formation, without requiring defect motion. Motivated by this observation, we show that a computational model of repulsive cell-cell interactions generates a mosaic with grain boundaries. In contrast to paradigmatic models of fate specification in mostly motionless cell packings, this finding emphasizes the role of cell motion, guided by cell-cell interactions during differentiation, in forming biological crystals. Such a route to the formation of regular patterns may be especially valuable in situations, like growth on a curved surface, where the resulting long-ranged, elastic, effective interactions between defects can help to group them into grain boundaries.

## Author summary

From hair cells in the mammalian inner ear to the bristles on a fly’s back, sensory cells often form precise arrays, ensuring that these cells are evenly spread out on the tissue’s surface. Here we consider the zebrafish cone mosaic, a crystal of cone photoreceptors in the outer retinal layer. Because the cone mosaic grows from the rim of the curved retinal surface, new rows of cones (*i*.*e*., defects) are inserted to maintain constant spacing between sensory cells. We study the spatial distribution of these defects to gain insight into how the cone pattern forms. By imaging retinae in live fish, we find that as differentiating cones are incorporated into the mosaic, defects form lines (grain boundaries) that separate mostly defect-free domains. Then, we show that a computational model based on repulsion between mobile cells during their incorporation into the mosaic generates similar grain boundaries. This study thus suggests that cell motion governed by repulsive cell-cell interactions can play an important role in establishing regular patterns in living systems.

## Introduction

In epithelial sheets that sense an external stimulus, the sensory function often depends on the spatial ordering of the constituent cells. In several examples [1–7], the pattern is sufficiently precise that if one knows the fate of just one cell, one can determine the identities of all the others. It remains a major challenge to understand how these extraordinarily regular cell arrays are created during development. Here we focus on one such system, the photoreceptor cell layer in the zebrafish retina, in which cone photoreceptors are organized by spectral subtype into a crystalline, two-dimensional lattice called the cone mosaic [8–10]; in particular, we use defects in this lattice as a window into possible mechanisms of mosaic formation. Although the precise evolutionary advantage and functional significance of the cone mosaic remain unknown, establishing an organized lattice in which each cone maintains some characteristic spacing from neighboring cones of the same subtype is thought to optimize sensitivity to a broad range of wavelengths over the full spatial extent of the retina [11–13].

**Fig 1:**
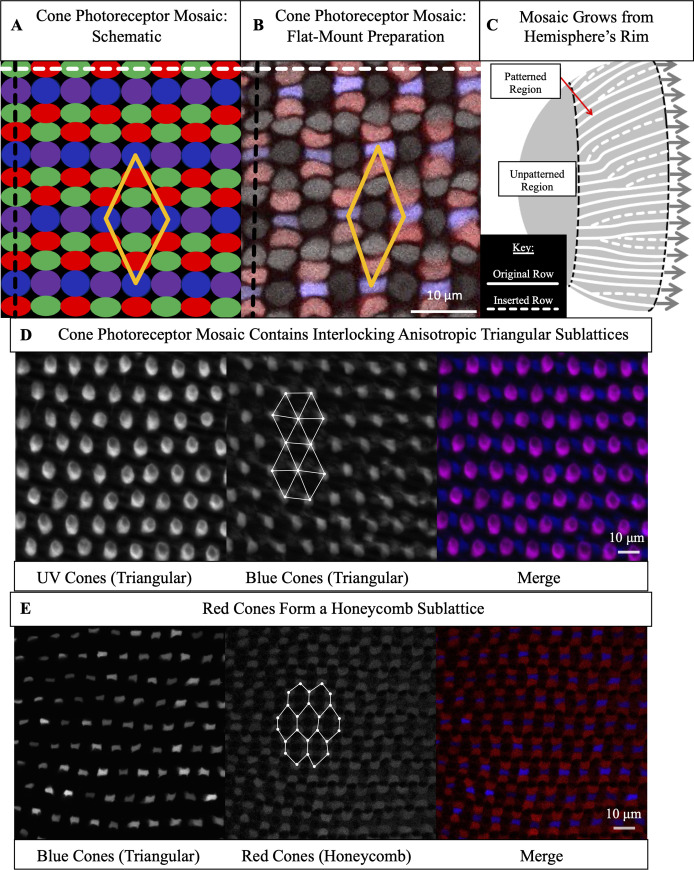
The cone mosaic is composed of four interpenetrating sublattices: two triangular sublattices and two honeycomb sublattices. (A) Schematic of cone photoreceptors (colored by subtype) in apical plane of zebrafish retina. The ‘unit cell’ (yellow parallelogram) contains one UV cone, one Blue cone, two Green cones, and two Red cones. White dashed line: ‘row’ axis. Black dashed line: ‘column’ axis. (B) Cone mosaic from flat-mount retinal preparation of an adult, triple transgenic fish, Tg[*sws2*:*GFP*; *trβ2*:*tdTomato*; *gnat2*:*CFP*]. Blue cones express a fluorescent reporter (pseudo-colored blue) under control of the *sws2* promoter, and Red cones express a fluorescent reporter (pseudo-colored red) under control of the *trβ2* promoter. All cones express an additional reporter (pseudo-colored gray) under control of the *gnat2* promoter. We distinguish between UV and Green cone subtypes based on morphology. (C) Schematic of photoreceptor epithelium, lining the outer surface of the hemispheric retina. The central retina, which surrounds the hemispheric pole and forms during the larval period, is unpatterned. As the retina grows by mitotic addition of new photoreceptors (and other retinal cells) at the hemispheric rim (gray arrows), there is a disorder-to-order transition (black dashed line). After this transition, the cone mosaic grows by neurogenesis at the hemispheric rim throughout the fish’s life. Because the hemispheric circumference grows, rows of cells are inserted to maintain approximately constant cell spacing. (D) UV and Blue cones in flat-mount retinal preparation from a double transgenic (Tg[*sws1*:*GFP*; *sws2*:*mCherry*]) line in which UV and Blue cones express distinct fluorescent reporters. UV cones (pseudo-colored magenta) form an anisotropic triangular sublattice that interpenetrates with an anisotropic triangular sublattice of Blue cones (pseudo-colored blue). We connect (white lines) a subset of nearest neighbors in the Blue cone sublattice. (E) Blue (pseudo-colored blue) and Red (pseudo-colored red) cones in flat-mount retinal preparation from panel B. Red cones neighbor Blue cones in each column. The Red cones form an anisotropic honeycomb sublattice. We connect (white lines) a subset of nearest neighbors in the Red cone sublattice; note the different nearest neighbor patterns in the Blue cone triangular sublattice (panel D) and the Red cone honeycomb sublattice (panel E). The Green cones form a honeycomb sublattice (not shown here).

Four spectral subtypes form the zebrafish cone mosaic: Red, Green, Blue, and Ultraviolet (UV) [14,15]. The ‘unit cell’, or the smallest repeating unit necessary to build the entire lattice, is composed of one Blue cone, one UV cone, two Green cones, and two Red cones (Fig 1A and 1B). Blue and UV cones form interpenetrating anisotropic triangular sublattices (Fig 1D). Green and Red cones form interpenetrating anisotropic honeycomb sublattices (Fig 1E). Along ‘rows’, Blue cones alternate with UV cones, and Red cones alternate with Green cones (Fig 1A and 1B). Along ‘columns’, each Blue cone is flanked by two Red cones, and each UV cone is flanked by two Green cones (Fig 1A and 1B). Rows radiate from the center of the retina to the periphery. Columns are approximately parallel to the rim of the retina (Fig 1A-1C).

The retinal hemisphere grows outward from the rim by mitotic addition of new photoreceptors (and other retinal cells) (Fig 1C) [16–19]. Until approximately two to three weeks post-fertilization, the newly incorporated cones are not arranged in an ordered mosaic [20,21]. Then, a disorder-to-order transition, in which newly incorporated cones begin to form a regular lattice, occurs. The region of cones generated earlier than this transition, called the ‘larval remnant’, remains disordered [20,21]. We call the rows that originate from the boundary of the larval remnant the ‘original rows’ (Fig 1C).

As new cells are incorporated at the rim, the circumference of the retinal hemisphere enlarges, and the spacing between the original rows necessarily increases (Fig 1C). To maintain approximately constant spacing between rows, new rows, that do not originate at the larval remnant, are inserted (Fig 1C). The topological defects that generate new rows are called Y-Junctions [20,22]. For a crystal on a spherical (closed) surface, defects are inevitable, as required by Euler’s formula [23–26]. In contrast, defects in the hemispheric photoreceptor layer, a non-closed surface, result not from a fundamental topological constraint but from the biophysical requirement to maintain reasonable cell sizes and not to leave gaps between cells in the retinal epithelium.

The generation of topological defects to maintain approximately constant cell spacing during growth on a curved surface makes the cone mosaic distinct from other patterned tissues, such as sensory bristles [27] and R8 photoreceptors in *Drosophila* [2,3,5,7]. Previous investigators have noted the existence of defects in the teleost cone mosaic [20,22]. Because these topological defects can provide insight into the biological mechanisms of pattern formation [28–30], in this paper we characterize the spatial distribution of each cone subtype in the Y-Junction core and compare Y-junction cores to defect cores in physical crystals. We show that a Y-Junction is a dislocation [31,32], the insertion of a row and a column.

Additionally, we characterize the spatial distribution of Y-Junctions in the retinae. We demonstrate that this distribution is as expected in a physical crystal on a hemisphere near an energy minimum [23,33–38]. As in a physical crystal, the defects form lines, called grain boundaries, from the center of the retina to the periphery [23,33–38]. In a physical crystal at finite temperature, defects are mobile; therefore, defects can coalesce into grain boundaries after formation of the crystal, regardless of the initial spatial distribution of defects [31,32,36,38,39]. We demonstrate that in the zebrafish retina, in contrast, grain boundaries appear during initial mosaic formation and do not require subsequent defect motion.

Having observed grain boundaries in fish retinae, we seek to take advantage of this finding to gain insight into the mechanisms of cone mosaic formation. We previously reported that cones of different subtypes are in approximately correct locations relative to each other within hours after they are generated by the terminal divisions of progenitor cells [40]. Though previous studies have documented interactions between cones in mature columns [21,41], little is known about the mechanisms by which premature columns initially form; in particular, the genetic and signaling networks that lead to spectral fate specification remain almost completely unexplored. Evidence from embryonic retina suggests that the spectral subtype of each cone is determined at the time of a symmetric, terminal division of its precursor [42]. If this finding from embryonic retinae holds for juvenile and adult retinae, it implies that the two daughter cells of the same subtype must move away from each other after their birth in order to reach the correct positions in the cone mosaic. This would suggest that interactions between differentiating cones with an established subtype generate crystalline order as these cones are incorporated into the retina.

Inspired by this evidence from embryonic retinae as well as other examples of neural cell mosaics [43–47], we propose a computational model in which fate-committed cells repel each other in an anisotropic medium. This model generates grain boundaries during initial mosaic formation, consistent with our observations of fish retinae. We, then, contrast our model of motile, fate-committed cells with a second model in which cells are neither fate-committed nor motile. In this second model, inspired by the example of Notch-mediated lateral inhibition in neural fate specification, static cells in a disordered packing signal to each other at short range to set up a fate pattern [3,5–7,27,48,49]. Because the signaling range must be roughly the cell size to produce a pattern of the correct wavelength, we find that in the absence of cell motion, this mechanism generates many excess defects (in line with a very recent, independent study [1]). We conclude that our model of motile, fate-committed cells is more consistent with observations of cone mosaic formation than a model of cell-cell signaling in a disordered packing.

The biological example of grain boundary formation during initial patterning in zebrafish retinae also poses interesting physical questions. A primary concern in the existing physics literature has been the existence of grain boundaries in the ground state of crystals on curved surfaces [23,24,26,34,50,51]; although some aspects of the kinetics of crystal growth on curved surfaces have also been considered [52,53], the question of how the growth geometry affects the positioning of defects has received little attention [39,54]. For example, in which growth geometries does crystallization produce defect distributions that are close to the ground state without defect motion? We show that for crystal growth in geometries comparable to the zebrafish retina, repulsive cell-cell (more generally, particle-particle) interactions produce just such low energy defect distributions during the initial growth process.

In the remainder of this paper, after characterizing the spatial distribution of each cone subtype in the Y-Junction core, we demonstrate the presence of grain boundaries in fish retinae. To quantify whether grain boundary formation occurs via defect motion, we track motion of individual defects in the retina in live fish. By comparing the timescales of defect motion and grain boundary growth, we conclude that grain boundaries form as cones are initially incorporated into the mosaic. We explain why cone mosaic formation is unlikely to occur via fate specification in a static, disordered cell packing, and we test a model of cell motion guided by cell-cell repulsion in an anisotropic medium. The latter model generates grain boundaries during initial mosaic formation, consistent with our observations of the retina.

## Results

### A Y-Junction is the insertion of a row and a column in the cone mosaic

**Fig 2:**
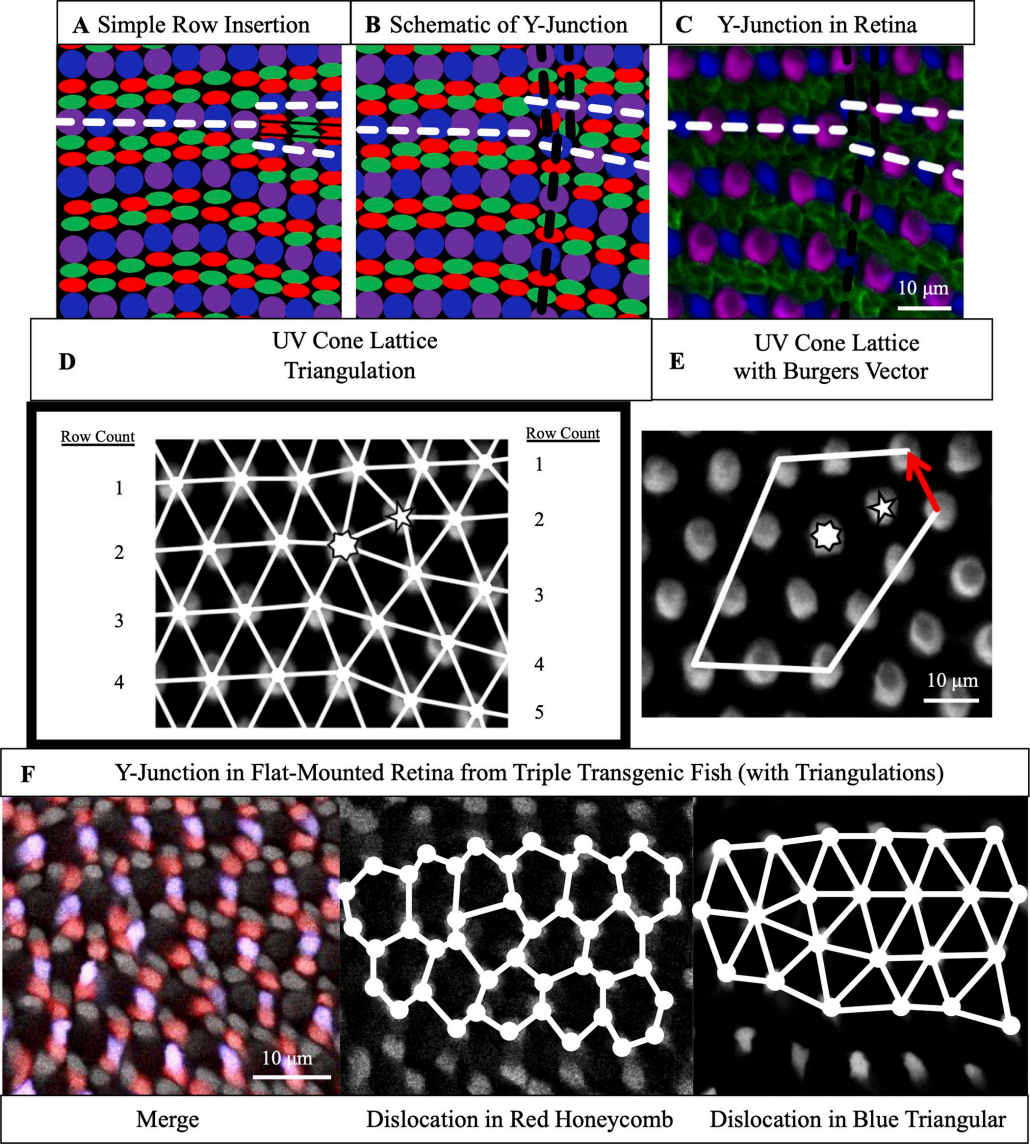
A Y-Junction, a topological defect in the cone mosaic, is an insertion of a row and a column. (A) Schematic of simple row insertion in cone mosaic. As new cone photoreceptors are incorporated to the right of the defect, a series of improper cone contacts (black box) within columns form. White dashed lines: rows associated with defect. (B) Schematic of a Y-Junction, a topological defect in the zebrafish cone mosaic. A Y-Junction only disrupts the cone mosaic near the core rather than along an entire line of contacts. White (black) dashed lines: rows (columns) associated with the defect. (C) A Y-Junction in a flat-mount retinal preparation from an adult, double transgenic (Tg[*sws1*:*GFP*; *sws2*:*mCherry*]) line in which UV and Blue cones express distinct fluorescent reporters (pseudo-colored magenta and blue, respectively) under control of UV and Blue opsin promoters, respectively. Antibody staining labels Red and Green cones (both pseudo-colored green). White (black) dashed lines: rows (columns) associated with the defect. (D) Each UV cone from panel C is connected (white bonds) to its nearest UV cone neighbors. To the left and right of the defect, rows are counted. Seven-sided (five-sided) star: seven-coordinated (five-coordinated) UV cone. (E) A circuit of triangulation bonds around defect from panels C-D. Red arrow is the Burgers vector, the additional bond necessary to close the circuit containing a dislocation. (F) Y-Junction in the same flat-mount retinal preparation as in Fig 1B. The round cells with dim fluorescence are UV cones. The Red cones are pseudo-colored red. The Blue cones are pseudo-colored blue. The remaining cones (bright gray fluorescence) are Green cones. We connect (solid white lines) nearest neighbors in the Red cone sublattice and Blue cone sublattice.

To maintain approximately constant cell spacing as the retina grows by mitotic addition of cone photoreceptors at the rim, rows must be inserted (Fig 1C) [20,22]. It is straightforward to demonstrate that a simple row insertion causes a disruption, that is not limited to a point defect but extends along an entire line, in the cone mosaic (Fig 2A). To avoid this disruption along an entire line of the cone mosaic, it is necessary to consider more complex defects: the insertion of two rows (S1 Fig) or the insertion of a row and a column, neither of which disrupts formation of the cone mosaic. In the zebrafish cone mosaic, the most common topological defect is the insertion of a row and a column, *i*.*e*., a ‘Y-Junction’ (Fig 2B and 2C). To understand why the insertion of a row and a column is expected to be the most prevalent defect, we employ a tool used in analyzing defects in physical crystals: the Burgers vector [31,32].

### Y-Junction generates minimal lattice deformation, as quantified by Burgers vector

As discussed in the introduction, the unit cell of the cone mosaic is composed of one Blue cone, one UV cone, two Green cones, and two Red cones (Fig 1A and 1B). One can generate an infinite cone mosaic on a flat plane given the unit cell and two lattice vectors, which define the Bravais lattice [55]. The Bravais lattice defines which defects one expects to observe in the cone mosaic lattice, though the distribution of particles in the defect core may vary [31,32,55]. For the sake of clarity, we analyze the defects in the cone mosaic from the perspective of a cone subtype that appears only once in the unit cell: UV cones.

To define the Burgers vector, we build a triangulation for the UV cones in which we connect nearest neighbors of the same cone subtype (Fig 2D and 2E). Away from the core of the defect, every UV cone is surrounded by six nearest UV cone neighbors [31,32,55]. Near the defect core, as in physical crystals that are triangular, one UV cone is surrounded by seven nearest UV cone neighbors. Neighboring this seven-coordinated UV cone is a UV cone that has only five nearest UV cone neighbors. This pair of five- and seven-coordinated UV cones constitutes the core of the dislocation.

We, then, construct a circuit that surrounds the core of the defect. If there were no defect, the circuit would be a parallelogram (Fig 2E). The bottom side of the circuit would contain as many bonds as the top side of the circuit. The right side of the circuit would contain as many bonds as the left side of the circuit. If there is a dislocation inside of the circuit, to close the circuit, one must add a bond, called the Burgers vector [31,32]. The magnitude of the Burgers vector quantifies the amount of lattice deformation associated with the dislocation [31,32].

In physical crystals, where the elastic deformation associated with the dislocation is proportional to the magnitude of the Burgers vector squared, the defect that generates the least deformation is expected to be the most prevalent. Even though we have no reason *a priori* to treat this biological crystal as elastic, we expect this measure of deformation to be generally applicable. The mechanism that drives ordering in a non-physical crystal likely also resists large deformations due to defects in the lattice.

The Y-Junction in the cone mosaic lattice is the dislocation that introduces the smallest deformation. This can be seen by comparing the Burgers vector of a Y-Junction to the Burgers vector of a double row insertion. For a double row insertion, the length of the Burgers vector is equal to the spacing between UV cones along a column, which is approximately twelve microns, as compared to the Burgers vector of a Y-Junction, with a length of approximately eight microns (for quantification of spacings between UV cones in same column and in same row, see S2 Fig). For the sake of minimizing lattice deformations, we expect double row insertions to be less prevalent than Y-Junctions.

### Distribution of Red and Green cones near the Y-Junction core

For Red and Green cones, that each appear twice in the unit cell, we connect nearest neighbors of the same subtype and analyze the spatial distribution in the Y-Junction core. Away from a Y-Junction core, the cells that form the lattice can be grouped into hexagons (Figs 1E and 2F; S3 Fig) [55–57], but this grouping breaks down near the defect core. The distribution of Red and Green cones in the Y-Junction core is variable but is often either a ‘glide’ dislocation or a ‘shuffle’ dislocation, which differ in the distributions of cones at the core.

A ‘glide’ dislocation has a heptagon and a pentagon in the core. For example, in S3A and S3B Fig, one heptagon of Red cones neighbors a pentagon of Red cones. A ‘shuffle’ dislocation has a single octagon in the core. For Red cones (S3E Fig), the octagon contains two UV cones, and for Green cones (S3D and S3F Fig), the octagon contains two Blue cones. Interestingly, both ‘glide’ and ‘shuffle’ dislocations are commonly observed in honeycomb crystals like graphene [56,57].

### Y-Junctions form lines, called grain boundaries, from center of retina to its periphery

Having verified that individual Y-Junctions are the dislocations that generate minimal lattice deformation, we next study the spatial distribution of Y-Junctions on the retinal surface. On the retinal hemisphere, the row direction rotates by 2π about the pole of the hemisphere, similar to the convergence of longitudinal lines toward a pole on the globe (Fig 1C). For physical crystals in this orientation, the ground state contains lines of dislocations, called grain boundaries, from the center (pole) of the hemisphere to the edge (equator) [23,58]. In physical crystals, dislocations are usually mobile; therefore, in physical crystals, it is possible for defects to rearrange into grain boundaries after crystallization, regardless of the initial spatial distribution of defects [31,32,36,38,39,58].

In a biological crystal, it is not obvious that the Y-Junctions will form a spatial pattern that is equivalent to the ground state of a physical crystal. If Y-Junctions do form grain boundaries, however, we may be able to leverage that information to understand the mechanism by which the biological crystal forms. By manually tracing rows of UV cones over approximately fifty percent of the retinal area in eight retinae (see Row tracing of flat-mounted retinae, S1 Table), we identified the locations of approximately one thousand seven hundred Y-Junctions.

### In flat-mounted retinae, a large fraction of Y-Junctions form grain boundaries

**Fig 3:**
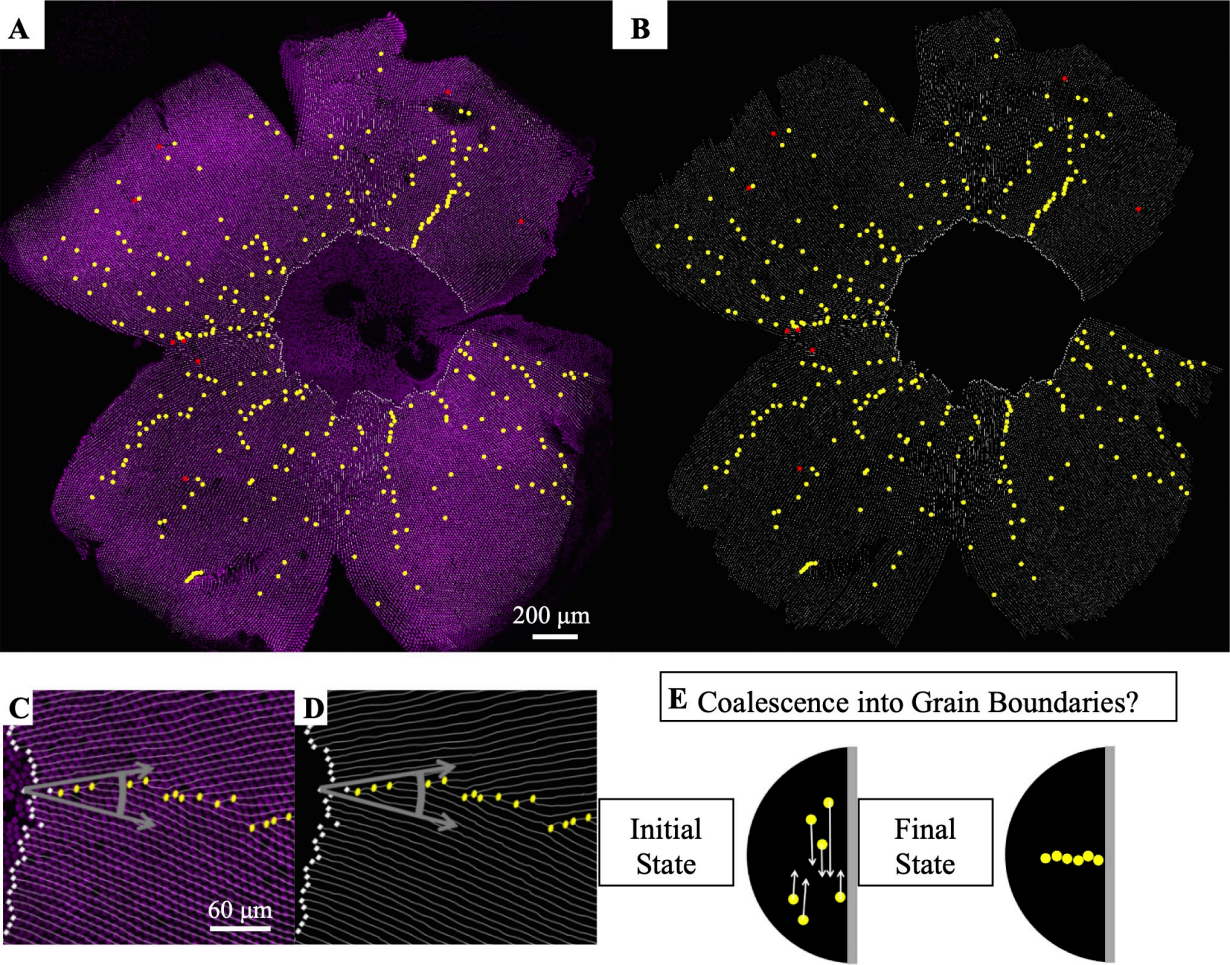
Y-Junctions form lines, called grain boundaries, from the center of the retina to the periphery. (A) Flat-mounted retina in which UV cones express a transgenic reporter (pseudo-colored magenta) under control of the UV cone opsin promoter. The dorsal side of the retina is left, ventral is right, temporal is down, and nasal is up. White lines: rows of UV cones. Yellow dots: Y-Junctions. Red dots: reverse Y-Junctions, generating row deletions. (B) Row tracing and identification of defects from retina in panel A. (C) Grain boundary from the retina in panel A. White squares indicate onset of patterning. Gray arrows indicate rotation of crystallographic orientation at the grain boundary. (D) Grain boundary presented in panel C with only the row tracing. White squares indicate onset of patterning. (E) Illustration of potential role of defect motion in generating the final spatial distribution of defects. Black region: photoreceptor epithelium. Gray region: retinal margin, where photoreceptor epithelium grows. Yellow circles: Y-Junctions. If defect motion does occur, it could allow defects to move together to form grain boundaries, as indicated by the white arrows. If defect motion is too slow, the patterning mechanism would instead have to generate grain boundaries during initial mosaic formation (not shown).

Y-Junctions do, indeed, form grain boundaries that run from the center of the retina to the periphery (Figs 3A, 3B and S4). These grain boundaries reconcile domains of differing crystallographic orientations (Fig 3C and 3D). The angle by which the local row direction rotates from one side of a grain boundary to the other side is determined by the linear density of Y-Junctions in the grain boundary and the length of the Burgers vector of an individual Y-Junction [31,32,58].

Although these grain boundaries can be picked out by eye, we developed a less subjective way to identify them, which can be applied to biological data and, later, to simulation results of potential models of cone mosaic formation. Before we describe this method, however, it is worth noting that although the existence of a grain boundary in the idealized case of a single boundary in an infinite crystal is unambiguous, there is no completely sharp definition of a grain boundary in a finite-sized system like ours with a non-zero defect density. If the dislocations needed to accommodate the cone mosaic to the hemispherical surface are uniformly distributed across the retina, then we do not want to say that grain boundaries are present, whereas if these defects are arranged in perfectly straight lines, we certainly do. There is necessarily some measure of arbitrariness, however, in where exactly we draw the line between these two limiting cases. Importantly, we will use our method of identifying grain boundaries primarily to compare the fraction of Y-junctions in grain boundaries in experimental images to the corresponding fraction in simulation results. We will use the same cutoff to define a grain boundary in both sorts of data, and we are able to show that our central conclusions are independent of the precise choice of cutoff.

To determine whether a specific Y-Junction is in a grain boundary, we quantify how much the row orientation rotates in the vicinity of the Y-Junction; we, then, check whether that rotation of row orientation is above a fixed threshold (Fig 3C and 3D; see Detection of grain boundaries in flat-mounted, row-traced retinae). By applying this measure to the eight analyzed retinae, we found that a majority of the identified Y-Junctions are in grain boundaries (S4 Fig and Tables 1 and S2).

**Table 1:** Quantification of grain boundaries in flat-mounted retinae. From the row tracing of eight retinae, we show the percentage of retinal area analyzed, the number of Y-Junctions identified, and the percentage of Y-Junctions in grain boundaries (see Detection of grain boundaries in flat-mounted, row-traced retinae). Here we assume that the rotation of row orientation about a Y-Junction must be greater than twelve degrees for that Y-Junction to be in a grain boundary, but we systematically scan that (arbitrary) threshold in S2 Table.

Fish #	Retinal Area (10^6^ *μm*^2^)	% Area Analyzed	Y-Junction Count	% of Y-Junctions in GB
1	2.4	47	155	85
2	2.1	46	166	83
3	3.9	57	221	86
4	3.8	70	275	69
5	5.3	66	249	78
6	4.2	61	184	85
7	5.4	51	182	77
8	5.4	63	285	73

### Defect motion is not responsible for grain boundary formation

To study whether defect motion is responsible for the existence of grain boundaries, we need to observe the dynamics of individual cones during cone mosaic formation in live fish rather than the fixed positions of cones in flat-mounted retinae. If defect motion is not responsible for the existence of grain boundaries, we will be able to test potential models of cone mosaic formation based on their ability to form grain boundaries at the boundary of an expanding cellular crystal.

To quantify the motion of individual UV cones during cone mosaic development, one must observe the same region of the retina in the same fish at two distinct time points as the fish remains alive. To make sure that one can locate the same set of UV cones at the two distinct time points, one must have some common point as a reference for UV cone positions in the two images.

**Fig 4:**
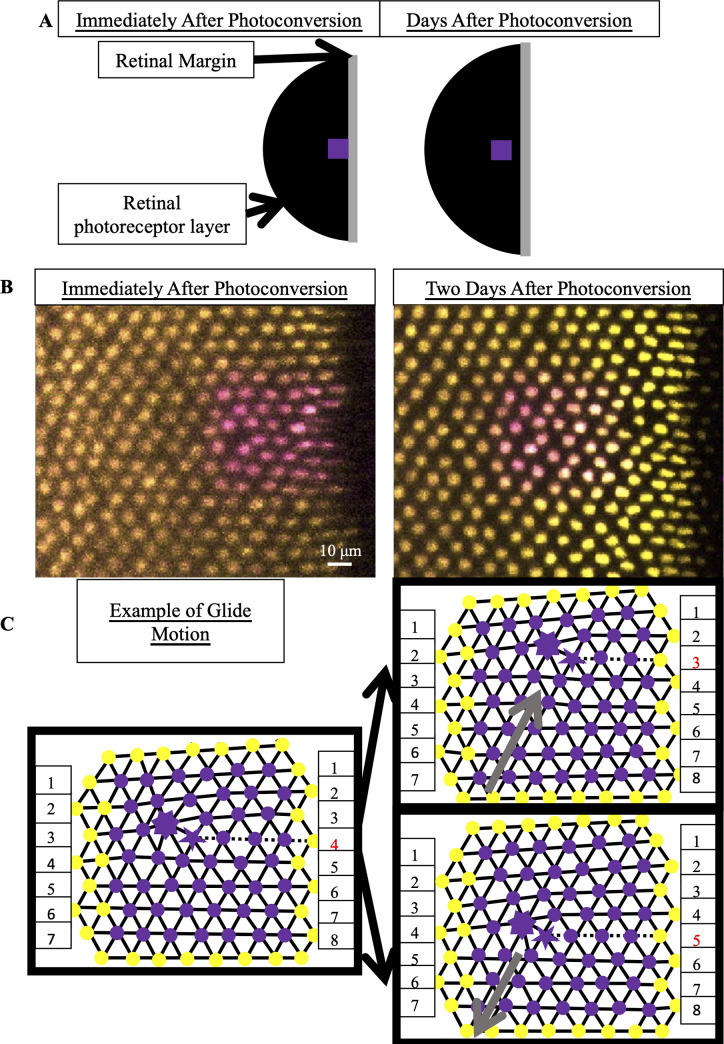
By photoconverting UV cones near the retinal margin, we track Y-Junction motion. (A) Schematic of photoconverted UV cones in photoreceptor epithelium near the retinal margin. We photoconvert a patch of UV cones (purple box) near the margin, where new cone photoreceptors are incorporated by mitotic addition. After two, three or four days of retinal growth, we image the photo-converted region, which is now separated from the margin by newly added retinal tissue (black). (B) Example of patch of UV cones immediately after photoconversion and two days later. In this line (Tg[*sws1*:*nEOS*]), UV cones express a nuclear-localized, photoconvertible fluorescent protein under control of the UV cone opsin promoter. The non-photoconverted fluorescent protein is pseudo-colored yellow, and the photoconverted fluorescent protein is pseudo-colored magenta. Retinal margin is to the right of each image. Approximately eight columns of UV cones have been added in two days since photoconversion. (C) Glide motion involves subtle motion of individual UV cones near the Y-junction core. Magenta circles are UV cones with photoconverted fluorescent signal, and yellow circles are surrounding UV cones with non-photoconverted fluorescent signal. Every cone is connected to nearest neighbors. Five-sided and seven-sided stars: five-coordinated and seven-coordinated UV cones, respectively. Dashed black line: the “inserted” row. The two triangulations on the right describe positions of UV cones (from the left triangulation) after glide in the direction denoted by gray arrow. Note that the assignment of five- and seven-coordinated UV cones has shifted by one row.

To locate the same set of UV cones at two different time points in live fish, we use transgenic zebrafish in which the UV cones express a nuclear-localized, photoconvertible fluorescent protein under the control of the UV cone opsin promoter (see Generation of transgenic zebrafish with nuclear-localized photoconvertible (green-to-red) EOS protein expressed specifically in UV cones and nEOS photoconversion and imaging). We photoconvert and image a small patch at the retinal margin, near where newly generated UV cones are incorporated into the growing retina (Fig 4A and 4B). At the time of imaging, the cone mosaic is composed of approximately eighty columns and is growing by approximately three columns of cones per day [40]. Two, three, or four days later, we image both photoconverted and non-photoconverted UV cones in the same retinal area (Fig 4A and 4B). By comparing the two images, we can determine how the different UV cones have moved relative to each other (see Tracking UV cone positions in photoconverted regions and measuring glide motion).

### Eliminating the possibility of defect motion perpendicular to the Burgers vector

To understand what types of motion we expect to observe, it is useful to revisit the concept of a Burgers vector. When a dislocation moves along the direction defined by the Burgers vector, this motion requires only subtle rearrangements of individual particles, here UV cones, near the core of the dislocation. This motion is called glide motion [31,32]. To illustrate this motion, we focus on the inserted row associated with the Y-Junction as well as the five- and seven-coordinated UV cones. When the dislocation glides by one row, as shown in Fig 4C, the initial inserted row incorporates itself into a neighboring row, as a new inserted row is generated. The assignment of the five- and seven-coordinated UV cones shifts by one row in the direction of the glide motion. Glide by one row is the flipping of one bond in the UV cone triangulation near the Y-Junction core.

On the other hand, motion of dislocations perpendicular to the direction defined by the Burgers vector, called climb motion, requires the creation or annihilation of point defects, which are interstitials or vacancies in the crystal [31,32]. A vacancy in the cone mosaic corresponds to the absence of an entire unit cell—that is, six contiguous cones—which we never observe (S5 Fig). Therefore, in monitoring the motion of individual UV cones, we test specifically for glide motion rather than climb motion [38].

### Quantifying glide motion

**Fig 5:**
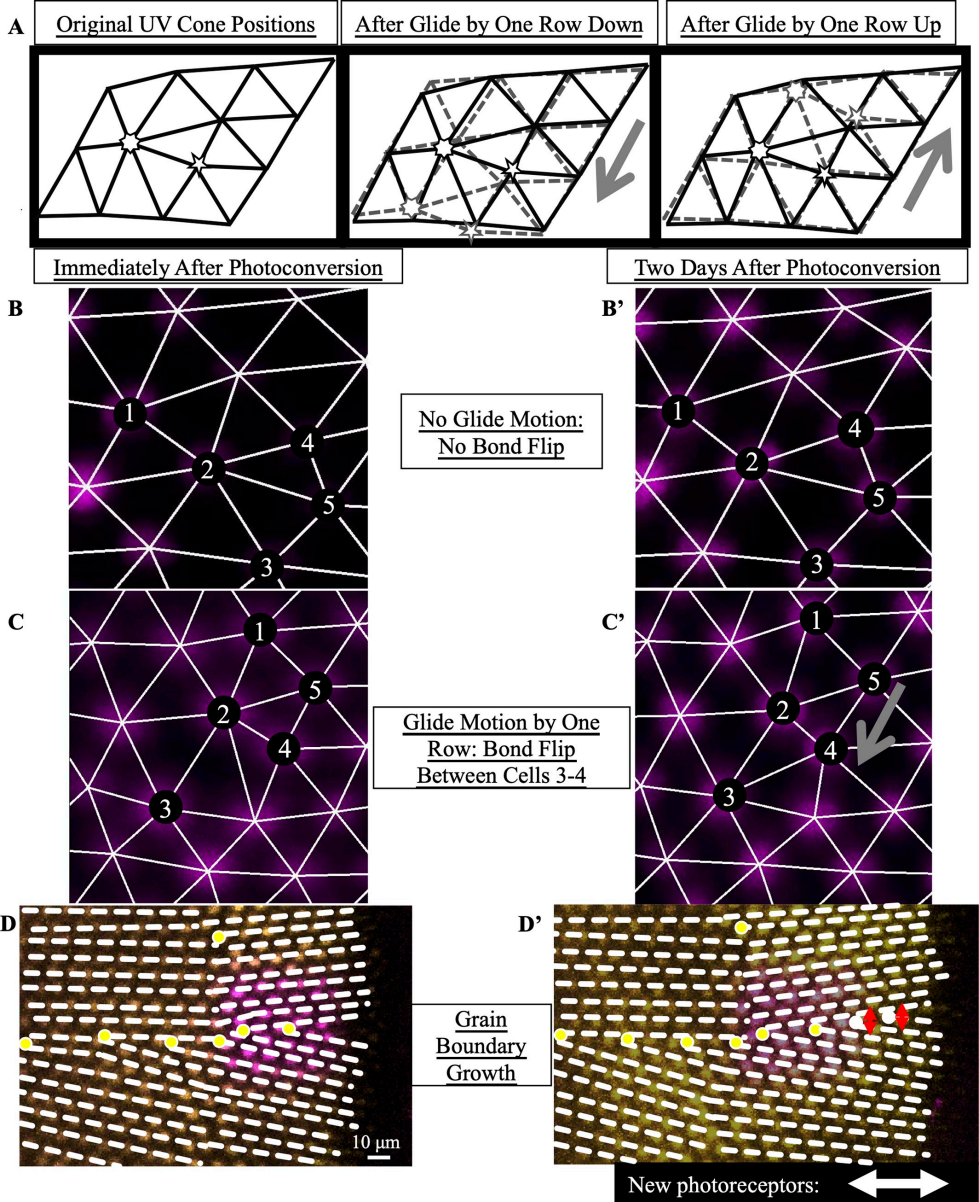
By estimating the timescale of Y-Junction motion, we conclude that Y-Junctions line up into grain boundaries during initial mosaic formation rather than by subsequent Y-Junction motion. (A) Expected motion of individual UV cones in the case of glide motion by one row in either direction. Left triangulation shows UV cones near the defect core; a UV cone sits on each site of triangulation. The center and right panels overlay the positions of UV cones before (black) and after (gray) glide in the direction denoted by the gray arrows. Note that the originally five- and seven-coordinated UV cones in the black triangulation both become six-coordinated. (B,B’) Example of Y-Junction in photoconverted region in which no bond flips in two days. The photoconverted fluorescent signal in UV cone nuclei is pseudo-colored magenta. For reference, the same five cones are numbered in both images. White lines: triangulation of UV cones. (C,C’) Y-Junction in photoconverted region from Fig 4B. One bond has flipped in the triangulation over two days, meaning that the Y-Junction has glided in the direction of the gray arrow by a row. (D,D’) Observation of grain boundary growth during initial mosaic formation. Immediately after photoconversion, one observes seven Y-Junctions (yellow dots), six within a grain boundary and an isolated Y-Junction nearby. White dashed lines: rows of UV cones. Two days later, one observes two additional Y-Junctions in the grain boundary. Based on the constraint that a Y-Junction does not glide faster than one row in two days, Y-Junctions must have initially formed within the regions indicated by red arrows (thus, within the grain boundary). White double-headed arrow: columns of cones incorporated since photoconversion.

Based on the positions of UV cones, we connect nearest neighbors in the lattice (see Tracking nuclear positions in photoconverted regions and Tracking UV cone positions in photoconverted regions and measuring glide motion). We identify the location of the Y-Junction core based on the location of the inserted row (Fig 5A). To quantify glide motion, we search for bond flips (Figs 4C and 5A) along the glide line (see Tracking UV cone positions in photoconverted regions and measuring glide motion) near the Y-Junction core between the time of photoconversion and the time of subsequent imaging (two, three, or four days later).

We observe non-negligible motion near the core; however, in our sample of sixteen Y-Junctions in thirteen distinct retinae (S3 Table), we never observe glide motion by more than one row per two days, where glide motion by one row is illustrated in Fig 4C. We show two examples of Y-Junctions within photoconverted regions: an example in which there is no glide motion (Fig 5B and 5B’) and an example in which the Y-Junction glides by one row in two days (Fig 5C and 5C’). These experiments thus provide an upper bound on the rate of glide motion of one row per two days. If we compare this constraint on the timescale of glide motion to the timescale of grain boundary formation, we can determine whether grain boundaries form via the coalescence of initially isolated dislocations that move together after initial crystallization.

### Comparing the timescale of glide motion to the timescale of grain boundary formation

In many of our photoconversion experiments, we photoconvert a patch of UV cones near at least one existing grain boundary (Figs 5D, 5D’ and S6). At the time of subsequent imaging of the UV cones near the photoconverted region (two to four days later), approximately eight UV cone columns are newly incorporated into the cone mosaic. After identifying Y-Junctions in the newly incorporated columns of UV cones, we can ask whether their locations are correlated with the positions of existing (*i*.*e*., observed at the time of photoconversion) grain boundaries. If we do observe a correlation between the positions of new Y-Junctions and existing lines of Y-Junctions, we can ask whether that correlation could be explained by glide of Y-junctions from completely random initial positions during the 2–4 days of observation or whether it must imply that new Y-junctions form preferentially near existing grain boundaries.

Table 2 lists the lines of Y-junctions we observed in our live imaging at the time of photoconversion; for each example, Table 2 also lists how much the row orientation rotates at the retinal margin. Similar to our identification of grain boundaries in flat-mounted retinae, we classify a line of Y-Junctions as a grain boundary if the rotation of the row orientation at the retinal margin exceeds a fixed cutoff (see Statistical significance of growing grain boundaries in live fish). For a cutoff of twelve degrees as in Table 1, out of the eighteen samples, nine samples have grain boundaries. Since some samples have two grain boundaries, in total we observe eleven grain boundaries above the twelve-degree cutoff in live fish (S6 Fig; and Tables 2 and S4). Two to four days later, we identify the positions of newly incorporated Y-Junctions. To these samples, we apply the following null model: each new Y-Junction’s initial position is uncorrelated with existing grain boundaries, but after formation, each new defect moves one row closer to the closest grain boundary per two days (see Statistical significance of growing grain boundaries in live fish). We find that newly incorporated defects are more aligned with existing grain boundaries than can be explained by this null model (*p*<0.0004). (We explore the effect of changing the threshold in row rotation angle used to define a grain boundary in S4 Table.) Because glide motion is slow relative to the grain boundary growth, we conclude that grain boundaries form at the time of cone mosaic formation, not by subsequent defect motion.

**Table 2:** Growth of grain boundaries in live fish. For images immediately after photoconversion, we measure how much the row orientation rotates at the retinal margin (see Statistical significance of growing grain boundaries in live fish). Although we define a grain boundary based on a rotation of the row orientation by twelve degrees or more, for the sake of completeness, this table lists all cases with a rotation of the row orientation of ten degrees or more. (See S4 Table for the effect of changing this angle cutoff.) For each of these cases, we count the number of defects in the corresponding grain boundary at the time of photoconversion and the number of defects added to the grain boundary by the time of later imaging. Though we photoconverted one region per fish, that region sometimes neighbors two grain boundaries, allowing us to measure growth of two grain boundaries in the same fish (*e*.*g*., 4–1 and 4–2). Fig 5D and 5D’ show grain boundary 3.

Line of Y-Junctions	1	2	3	4–1	4–2	5	6	7	8	9	10	11–1	11–2	12–1	12–2
Δ*t* (days)	2	2	2	2	2	2	2	2	2	2	3	4	4	4	4
Domain Rotation at Photoconversion (°)	24	19	16	19	17	10	14	11	12	12	16	18	10	16	14
Number of Defects at Photoconversion	4	8	6	4	9	4	8	5	5	6	3	4	3	2	2
Number of Defects Added Later	+5	+1	+2	+2	+2	+1	+2	+1	+0	+2	+2	+3	+2	+3	+0

### Testing computational models of cone mosaic formation

**Fig 6:**
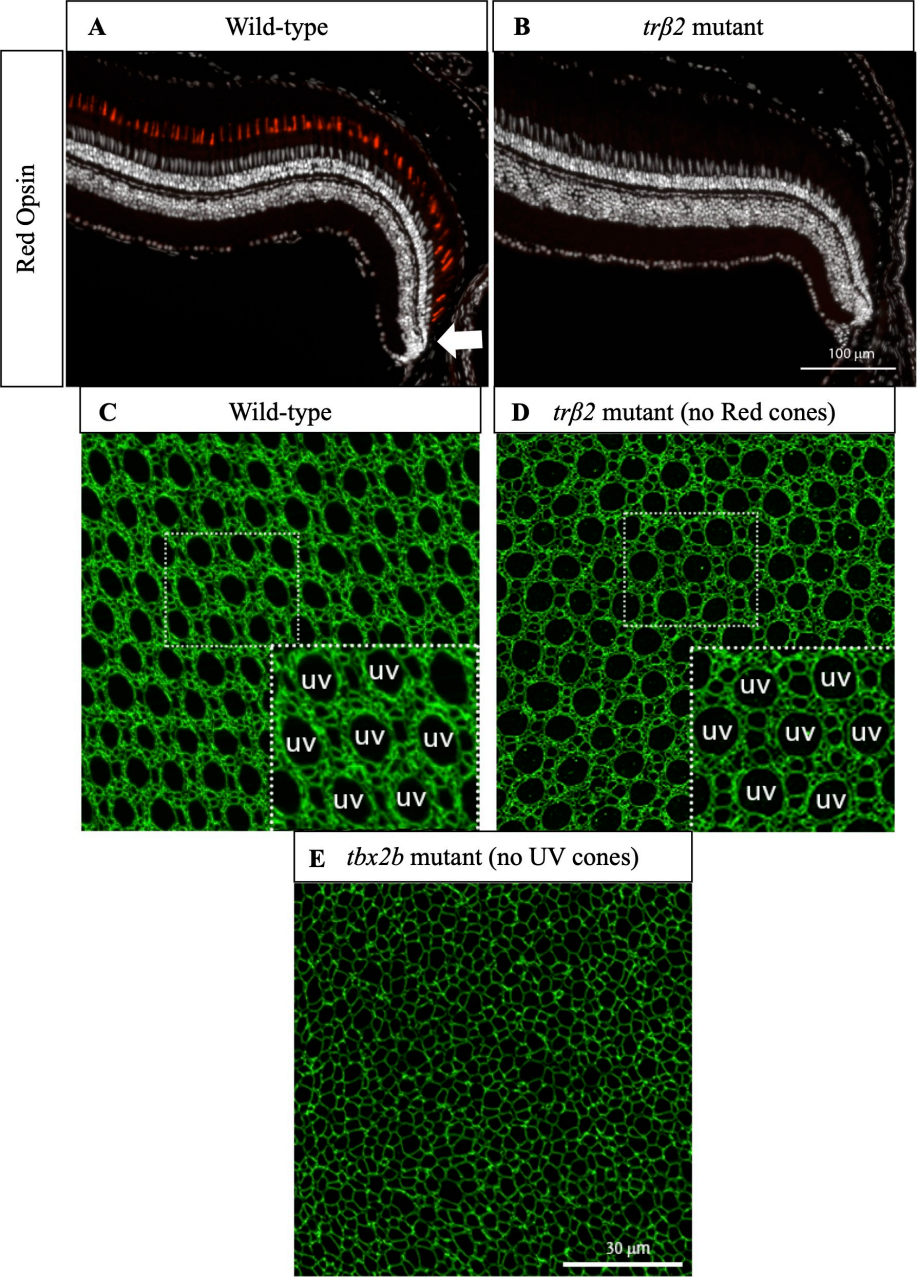
Cone mosaic formation is minimally disrupted in Red cone mutant but is significantly disrupted in UV cone mutant. (A) Cross-section of wild-type retina in which immunostaining of Red cone opsin labels Red cones. White arrow indicates approximate location of precolumn area [40]. (B) Cross-section of *trβ2* mutant in which immunostaining of Red cone opsin labels Red cones. Note absence of differentiated Red cones. White arrow indicates approximate location of precolumn area. (C) Apical plane of wild-type cone mosaic lattice in retinal flat-mount in which anti-ZO-1 stains cell profiles. UV cones are indicated in the inset. (D) Apical plane of cone mosaic in retinal flat-mount from *trβ2* mutant, which lacks Red cones. Anti-ZO-1 stains cell profiles. UV cones (indicated in inset) identified based on large, rounded profiles. The triangular lattice of UV cones is minimally disrupted in absence of Red cones. (E) Apical plane of cones in retinal flat-mount from *tbx2b* mutant, which lacks UV cones. The cone mosaic is disrupted in this mutant.

Near the rim of the retinal hemisphere (Fig 6A) is a region defined as the precolumn area, where newly generated but not fully differentiated cones are in approximately correct locations relative to each other based on cone subtype before the formation of mature columns [40]. Composed of differentiating post-mitotic cones, this precolumn area lies between two regions: a central region of mature columns and the rim, which contains proliferative cells. It remains unclear how proper positioning of cones by subtype in the precolumn area occurs, but importantly, this must occur within hours of the generation of post-mitotic cells by terminal divisions of neighboring proliferative cells [40].

In this section, we ask whether our observations of grain boundary creation can provide information about the mechanism for proper positioning of cones by subtype into immature columns. In principle, one can imagine two extremal models for the creation of regular cell fate patterns in biological tissues. In the first model, cell fates are specified first, and motile cells with a clear identity then rearrange themselves into the final pattern; in the second model, cells instead first arrange themselves in space, then the correct fate pattern is imposed on this static cell packing by cell-cell signaling. Evidence from embryonic retinae suggests that post-mitotic cones are of fixed subtype and move relative to each other during integration into the retina [42].

This finding from embryonic retinae, together with other examples of cell-cell repulsion in neural cell mosaics [43–47], suggests that mosaic formation in zebrafish retinae falls closer to the first model. To test whether such a picture is consistent with the observed behavior of grain boundaries, we construct a computational model in which fate-specified cones repel each other during differentiation. After finding that this cell-cell repulsion model does indeed generate grain boundaries during the initial process of differentiation, we demonstrate that the alternative model, in which cell fate patterns arise through lateral inhibition in a static and disordered packing, is not likely to be responsible for cone mosaic formation. Before turning to the descriptions of the two models, we first gather some additional experimental data, on which cone subtypes are essential to establishing a crystalline mosaic and on lattice anisotropy in the zebrafish mosaic, to help in model formulation.

### Absence of Red cones only minimally disrupts cone mosaic formation, but the absence of UV cones significantly disrupts mosaic formation

As noted above, crystals are described by identifying the smallest repeating unit that can be used to build the crystal, the unit cell–consisting of one Blue, one UV, two Red, and two Green cones (Fig 1A) for the zebrafish retina–and the way different unit cells are positioned relative to each other in space, the Bravais lattice–for the zebrafish retina, a slightly anisotropic triangular lattice [31,32,55]. It is well established that the defect core structures can depend on all the features of the unit cell but that elastic interactions between two defects are determined only by the Bravais lattice and the defects’ Burgers vectors [23,24,34,55,59]. Thus, we expect that most features of the spatial distribution of Y-junctions, which should depend primarily on defect-defect interactions [23,24,34,37,50,51], can be recapitulated by a model in which each unit cell is replaced by a single cone photoreceptor. In order to provide biological justification for this simplification of the cone mosaic and to determine which cone subtype to focus on, we consider mutants in which certain cone subtypes are absent.

To evaluate the role of Red cones in establishing a crystalline mosaic, we generated a targeted mutation in a single gene that resulted in a loss of Red cones (Fig 6B; see CRISPR-Cas9 mediated mutation in the *thrb* gene). This gene, *trß2*, is an early fate marker of Red cones and is expressed in proliferating progenitors and mature Red cones, but not other cone subtypes [40,42]. All other cone subtypes are still present in the outer retinal layer (S7A Fig). Strikingly, in the *trß2* mutant, we find that cone mosaic formation, including ordering of UV cones, is only minimally disrupted by the absence of Red cones (Figs 6D and S7B).

The robustness of cone mosaic formation to the absence of Red cones is in marked contrast with previous experiments with *tbx2b* mutants in which UV cones are largely absent [60]. In *tbx2b* mutants, it is difficult to discern long-ranged crystalline order in the cone positions (Fig 6E). The spatial distribution of cones and absence of long-ranged order are similar in other zebrafish mutants in which cell-cell adhesion is perturbed [21,41]. Given this evidence from both *trß2* mutants and *tbx2b* mutants, we simplify the cone mosaic to a lattice of UV cones.

### Measuring lattice anisotropy of the cone mosaic in live fish

Previous studies have established the importance of anisotropy of the mosaic lattice both in its formation and refinement [21,40–42]. In modeling the cone mosaic lattice as a lattice of UV cones, we need to make sure that we produce a lattice with the same anisotropic spacing as the cone mosaic. For this reason, we first measure the lattice vectors of the UV cone sublattice in live fish. We use images of photoconverted regions, immediately after photoconversion, in which there are no Y-Junctions (S2 Fig).

In an isotropic triangular lattice, the ratio of the distance between UV cones in the column direction to the distance between UV cones in the row direction is equal to the square root of three. We find that this ratio is approximately six-fifths in our live images of the UV cone sublattice (S2 Fig). This means that the spacing along the row direction is longer than would be expected in an isotropic triangular lattice. We use this degree of anisotropy as an input for modelling the cone mosaic formation.

### Model of repulsive interactions between fate-committed cells generates grain boundaries during initial cone mosaic formation

In building a model of cone mosaic formation, we hypothesize that cell motion is generated by repulsive interactions between fate-committed cones of the same subtype as they differentiate in a mechanically anisotropic medium. This model is motivated by cell mosaics in other vertebrate retinal layers [43,45–47] and in the nervous system in *Drosophila* larvae [44]. For example, in mice, specific neuron subtypes disperse after fate commitment and during morphological differentiation [45,47]. By cell ablation, previous investigators have established that for retinal horizontal cells in mice, the cells are fate-committed and homotypically interact via transient neurites (*i*.*e*., cell processes) [45]. Similarly, in *Drosophila*, certain classes of peripheral sensory neurons tile the body wall. By ablation of dendritic processes, previous investigators have established that neurons of a specific class (class IV) interact homotypically to establish a tiling of the body wall by non-overlapping cell territories [44].

**Fig 7:**
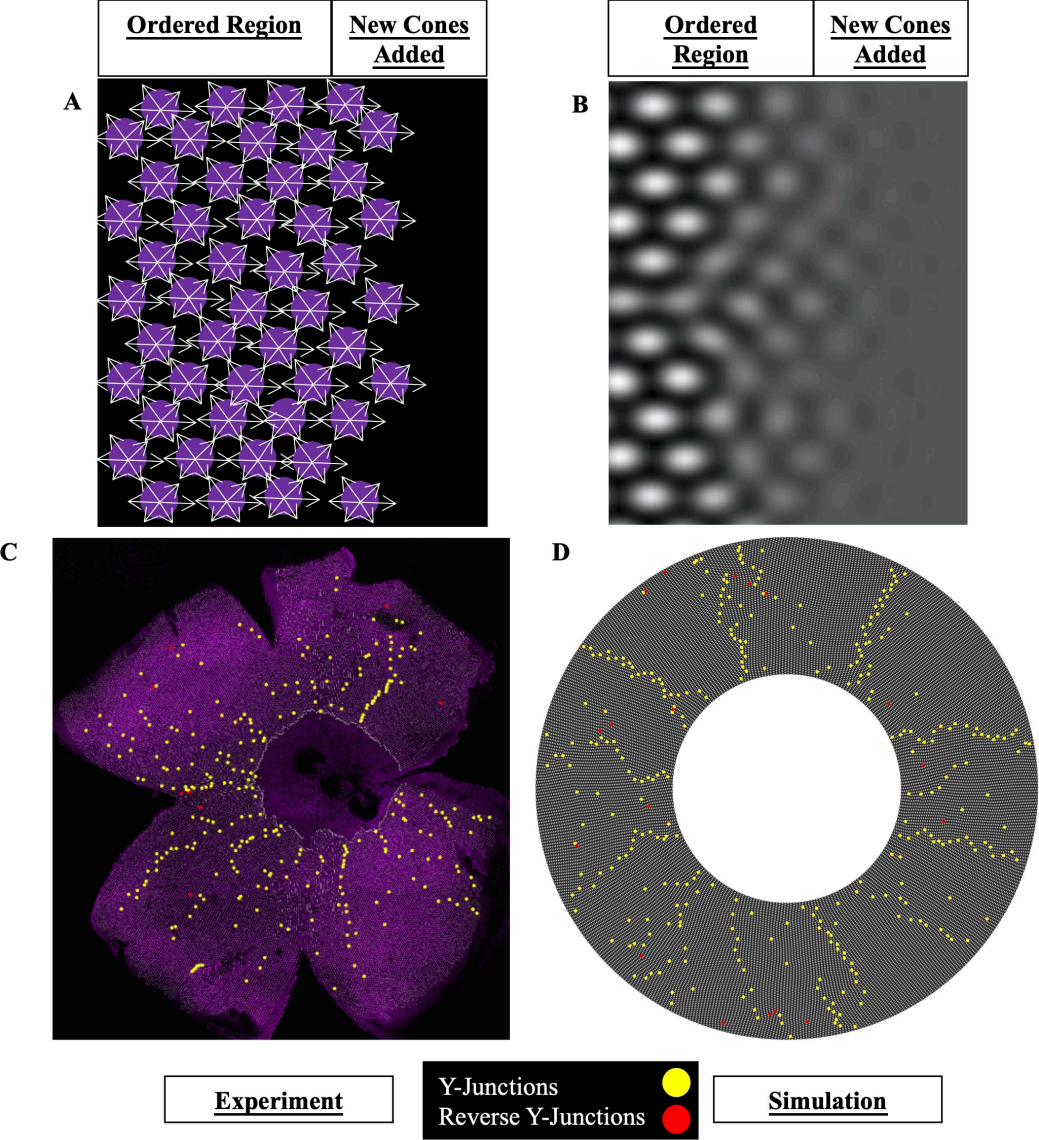
Phase-field crystal model of cone mosaic formation. (A) Schematic of contact-interaction model in which fate-committed cones interact homotypically and form an anisotropic lattice. UV cones (magenta circles) interact with nearest neighbors of same subtype. White arrows: contact interactions. New fate-committed cones are incorporated to right of the ordered region. (B) In the phase-field crystal model, a continuum field describes the positions of UV cones. UV cones are most likely to be found around peaks in the density (white regions) and least likely to be found in troughs (dark regions). (C) Flat-mounted retina (same as Fig 3A and 3B) in which UV cones express a transgenic reporter under control of the UV cone opsin promoter. Yellow dots: Y-Junctions. Red dots: reverse Y-Junctions. (D) Simulation of phase-field crystal model on the surface of a cone. The number of initial rows and total number of columns are comparable to those in retinae, and this leads to a defect density similar to that measured in experiments. The degree of anisotropy of the triangular lattice is constrained by the anisotropy in unit cell dimensions measured in our live-imaging experiments. Yellow dots: Y-Junctions. Red dots: reverse Y-Junctions.

To model repulsive interactions between fate-committed cones of the same subtype, leading to a preferred cell spacing in the row and column directions, we employ a phase-field model of crystallization (Fig 7A and 7B). These models are widely employed to describe various phenomena in physical crystals, where they are known to give qualitatively, and in some cases quantitatively, accurate descriptions of processes including nucleation of crystalline domains in a supercooled fluid and the mechanical hardness of a solid based on the microstructure of crystalline domains, while having vastly improved computational efficiency compared to particle-based models [61,62]. Importantly, the basic form of a phase-field crystal model can be determined from system symmetries and similar phenomenological considerations, so that these models in some sense provide a generic description of crystallization, independent of many specifics of the microscopic interactions between individual particles.

Instead of representing a crystal as composed of discrete particles, phase-field crystal models employ a continuum field, which corresponds to modulations in the particle density (Fig 7B) [61–68]. Particles are most likely to be found around peaks in the density and least likely to be found in troughs. Based on our simplification of the cone mosaic to a lattice of UV cones, the particles in our case can be identified with UV cones. If the field is homogeneous in space, the UV cones form a “liquid,” with no clear periodicity. If the field is periodic in space, they form an ordered crystal.

The phase-field crystal model assumes that the density field evolves to minimize a free energy functional while conserving the total number of particles. This dynamical law can be derived from a microscopic model of individual particles whose pairwise interactions include a short-ranged repulsion and that are subject to frictional damping and thermal noise; the approximations involved in the derivation amount in essence to assuming that higher-order correlations relax quickly compared to two-body correlations and that the crystal structure can be described by keeping only Fourier modes in the particle density of the dominant wavelength [62]. The existence of a dominant wavelength, in turn, arises from the fact that the particles repel each other at short range and thus have a preferred minimum spacing. (Although there is no *a priori* reason that the dynamics of a biological system should be governed by a free energy, we expect that many models with symmetric, pairwise, repulsive interactions between unpolarized cells can be mapped onto an effective free energy of the phase-field crystal form.)

To model the growth of the cone mosaic, we initialize the phase-field to be entirely uniform except near the center of the simulation domain (S1–S6 Videos; see Numerical solutions of anisotropic phase-field crystal model on cone). The crystalline region grows outward from the center, as fate-committed cones in the disordered region are incorporated (Fig 7A and 7B).

For a fixed anisotropy of the lattice spacing (S2 Fig), the simulation results depend on two free parameters in the model free energy. The first parameter specifies the degree of undercooling. When the temperature is below the melting temperature (*i*.*e*., the liquid is “undercooled”), the density field is unstable to the formation of periodic structures; otherwise, only the liquid phase is stable. As there is no clear analogue to temperature in our system, we interpret this parameter as quantifying the strength of the interactions relative to the random noise in cone cell motion. The second parameter is the mean of the cone density field, which is conserved by the dynamics.

The values of these two free parameters, the interaction strength and the mean of the density modulation field, determine which phase (or phases) are stable. Depending on these parameters, this model can generate three phases: a constant (“liquid”) phase, a striped phase, and a triangular phase [61,64]. We can constrain these two free parameters to the region of the phase diagram in which the only stable phase is the triangular phase. To ensure that any conclusions from the model do not depend on fine-tuning of parameters (S8 Fig; see Numerical solutions of anisotropic phase-field crystal model on cone), we scan the parameters over a one-dimensional cut of this region of the phase diagram, while also varying the magnitude of noise in the initial conditions.

By simulating the phase-field crystal model in a geometry of comparable size to the retina and with comparable defect densities, we find that this model generically produces spatial distributions of Y-Junctions that are similar to those observed in the flat-mounted retinae (Figs 3, 7C, 7D, S4 and S8 and S5 Table and S6 Video). As we scan over simulation parameters, the fraction of Y-Junctions in grain boundaries, as quantified by the same measure that we applied to the flat-mounted retinae with the same cutoff as in Table 1, is slightly greater than fifty percent on average (S8 and S9 Figs and S5 Table). For our simulations with the smallest magnitude of undercooling, the fraction of Y-Junctions in grain boundaries reaches approximately seventy percent. In the flat-mounted retinae, approximately seventy-five percent of Y-Junctions are in grain boundaries (Tables 1 and S2; see Detection of grain boundaries in simulated cone mosaic for a discussion of sources of error that could lead to a mild overcount of Y-Junctions in grain boundaries in flat-mounted retinae and see Alternative method for detection of grain boundaries for an alternative grain boundary detection method that avoids these issues at the expense of introducing additional *ad hoc* parameters). Thus, this model, for which there is supportive evidence in embryonic retinae [42], tends to group a majority of Y-Junctions into grain boundaries, consistent with observations of flat-mounted retinae (Figs 7C, 7D, S8 and S9 and Tables S2 and S5).

### Additional insights generated by model of cell-cell repulsive interactions

Armed with this cell-cell repulsion model, we now generate insights into cone mosaic formation. First, we address how the specific orientation of the cone mosaic lattice is selected and how it is maintained as the crystal grows. With isotropic interactions in an isotropic medium, at the onset of ordering, crystallites form with random orientations (S4 Video). Additionally, as the crystal grows outward, the crystallites tend to rotate into an orientation that is misaligned with the orientation observed in the retinae by thirty degrees (*i*.*e*., maximally misaligned) (S10A Fig and S3 and S4 Videos). For an anisotropic crystal, as in the retinae, the correct crystallographic orientation is selected even without spatial ordering in the original cone positions, and that crystallographic orientation is maintained as the crystal grows (S10B Fig and S2 and S5 Videos).

This model also suggests a mechanism by which grain boundaries form during initial mosaic formation. We consistently observe in our phase-field crystal simulations that the density profiles near a grain boundary remain poorly resolved even as neighboring regions of the crystal grow outward, leading to a characteristic V-shape of the crystal surface (S10C Fig). Our model predicts that there should be a lag in proper positioning of cones near a grain boundary.

### Implausibility of lateral inhibition mechanism for cone mosaic formation

Before concluding, we briefly discuss a possible alternative model of cone mosaic formation and consider its shortcomings in generating a crystalline mosaic. The alternative model is motivated by cell fate lattices, like those formed by sensory bristles or R8 photoreceptors in *Drosophila* [3,5–7,27,48,49], where neural cells are selected through a process of lateral inhibition mediated by the Notch signaling system. In these examples, cell motion is largely absent during pattern formation. Mathematical models of motionless tissues in which cells differentiate, inhibiting neighboring cells within some range from committing to the same fate, have had some success in reproducing the patterns observed in these systems [3,5,6,27,69–76].

Specifically, we adapt a lateral inhibition model originally developed to describe sensory bristle patterning in *Drosophila* [27] (see Numerical solutions of lateral inhibition on disordered cell packing). An important difference between sensory bristle patterning in *Drosophila* and cone mosaic formation in zebrafish is that the lattice vectors in the cone mosaic are much shorter, in units of cell diameters, than the lattice vectors of the sensory bristle lattice. There are five to six cells between nearest-neighbor sensory bristle cells [27], but only one to two cells between nearest-neighbor UV cones. Therefore, we expect a cone mosaic lattice generated by lateral inhibition to be more sensitive to disorder in cell packing than is the sensory bristle lattice.

In S11 Fig, we provide illustrative examples of lateral inhibition with different signaling ranges (see Numerical solutions of lateral inhibition on disordered cell packing). As a wave of differentiation moves from the left side of the cell packing to the right, individual cells differentiate into the inhibiting, neural cell fate (dark cells, *u*≈1), and these cells signal to their neighbors, causing them to adopt the alternative (non-neural, *u*≈0) fate. For the shortest inhibition range, appropriate for the UV cone mosaic, the resulting fate pattern has many defects, disrupting the long-ranged crystalline order expected in the cone mosaic [21,40]. Thus, in this model, the underlying disordered cell packing prevents the formation of a precise triangular lattice, even before we consider the effects of patterning on a curved surface. This contrasts with the results of the model discussed in previous sections in which fate specification occurs first and cells of known spectral fate then move into the correct lattice position.

Since the basic impediment to pattern formation by lateral inhibition in this model is the disorder in the cell packing, one might be tempted to consider a model in which lateral inhibition instead acts to impose a fate pattern on an ordered packing of equipotent cells. In such a model, however, there would have to be defects in the ordered packing for it to fit onto the retina’s curved surface, and the pattern of defects in the eventual cone mosaic might be expected to follow the pattern of defects in the underlying packing. Thus, the problem would again be reduced to that of arranging cells (necessarily with some short-ranged repulsion, as they cannot overlap) into a crystal on the surface of a growing hemisphere–that is, essentially to the same problem solved by our earlier phase-field model of cone mosaic formation.

## Discussion

In this paper, we characterize the properties of Y-junction defects in the zebrafish cone mosaic and their spatial distribution across the retina. Strikingly, Y-junctions are organized into grain boundaries oriented perpendicular to the retinal margin, as would be expected if they were positioned to minimize the elastic energy of a physical crystal [23,58]. We show, however, that unlike dislocations in most physical crystals [36,38], Y-junctions experience at most very limited diffusive motion, implying that they must be positioned within existing grain boundaries when they are created at the cone mosaic’s growing margin. Inspired by these observations, as well as previous findings that cone photoreceptors in embryonic retinae are born from symmetric terminal divisions and then disperse to their final positions [42], we propose a model for cone mosaic formation based on interactions among mobile, fate-committed cells. This model reproduces the major features of the Y-Junction distribution in the zebrafish retina.

Our model of cell motion contrasts with most previous pictures of pattern formation in sensory epithelia [3,5–7,27,48,49,69–76], which have instead relied on inhibitory signaling between immobile cells to generate fate mosaics. Many of these earlier studies assume the patterning process takes place on top of a perfectly crystalline arrangement of identical cells, and the ultimate fate pattern inherits some of its regularity from this underlying lattice. Mathematical models of lateral inhibition using disordered cell packings have successfully reproduced observed fate patterns when the scale of the pattern is relatively large, with typical spacings of order 5–6 cell diameters, as in bristle or R8 photoreceptor specification in *Drosophila* [3,5,6,27].

We have argued, on the other hand, that if the lateral inhibition signaling range is on the order of the typical cell size in a disordered packing, one must invoke cell rearrangement to produce a crystalline mosaic. This finding is consistent with a very recent examination of the patterning of outer hair cells in the mammalian inner ear [1], which form a triangular lattice with spacing on the order of one cell diameter [1,4]; in this case, global shear forces driving cell neighbor exchanges are required along with short-ranged inhibitory signaling to produce a roughly crystalline array of cell fates.

In contrast to these earlier studies that emphasize biochemical signaling between cells in fixed positions, our model of cone mosaic crystallization is based on interactions between motile cones of the same subtype (*e*.*g*., UV cones) during their differentiation and incorporation into the retina. We hypothesize that these repulsive interactions are mediated by cellular appendages called telodendria [77,78]. Previous investigators have suggested that telodendria may be important in tangential photoreceptor dispersion after symmetric terminal cell division in zebrafish retinae [42]. Though less ordered than the zebrafish cone mosaic, both the retinal horizontal cell mosaic in mice and class IV neural cell mosaic in *Drosophila* form via similar neurite-mediated interactions [43–47], and both homotypic and heterotypic repulsions have been proposed to mediate the formation of the disordered, hyperuniform avian cone mosaic [12], demonstrating the importance of cell motion guided by repulsive interactions for forming neural cell mosaics.

It is tempting to speculate about the merits of cone mosaic formation via homotypic cell-cell repulsion between fate-specified cones as opposed to a more classic lateral inhibition pathway. For example, if the formation of defect-free crystalline domains (separated by grain boundaries) is functionally relevant, which aspects of fish retinae might allow our model to outperform lateral inhibition in creating such domains? One possibility is that it is precisely the curvature of the retinal hemisphere and the resulting need for lattice defects that favor the homotypic repulsion mechanism over lateral inhibition. Indeed, we have argued that a cone mosaic formed in such a way will have many of the same features as a physical crystal. These features include, in particular, the presence of an effective long-ranged, elastic interaction between Y-Junctions that is absent in models of fate specification through signaling on a fixed cell packing. Such a long-ranged interaction likely makes it easier to position Y-Junctions correctly across the retina, as exemplified by the spontaneous appearance of grain boundaries in our phase-field crystal model.

Finally, our findings highlight the importance of the kinetics of crystal growth in determining the observed spatial distribution of defects. Many studies of physical crystals focus on the agreement between theoretically predicted ground-state defect patterns and experimentally observed defect distributions [23,24,34] rather than on the kinetics of grain boundary formation. In the zebrafish retina, in contrast, the effective absence of glide motion implies that the crystal cannot sample different defect arrangements to find the lowest energy but must instead place the defects correctly as the crystal forms. As in earlier work by Köhler *et al*. [52], our phase-field crystal simulations of cone mosaic formation suggest that dislocations are grouped into grain boundaries because of a delay in crystal growth near a grain boundary relative to growth of neighboring, defect-free domains. Biological tests of this prediction await further investigation.

## Materials and methods

### Zebrafish

Fish were maintained at approximately 28°C on a 14/10 h light/ dark cycle with standard husbandry procedures [79]. Zebrafish lines, *Tg(-5*.*5sws1*: *EGFP)kj9* [80], *Tg(-3*.*2sws2*: *mCherry)mi200*7 [21], *Tg(trβ2*: *tdTomato)* [42], *Tg(-3*.*2sws2*: *EGFP)* [81], Tg(*gnat2*:H2A-CFP), and pigment mutant *ruby* carrying *albino (slc45a)*^*b4/b4*^ and *roy*^*a9/a9*^ were used. All animal procedures were approved by the Institutional Animal Care and Use Committee at the University of Michigan.

### Histology

Retinal dissection, fixation and immunocytochemistry were performed as previously described [40,82,83]. Briefly, the isolated retina was fixed in 4% paraformaldehyde with 5% sucrose in 0.1M phosphate buffer, pH 7.4, at 4°C overnight. After antigen retrieval with 10 mM sodium citrate in 0.05% tween 20 (pH 6.0), the retina was incubated in blocking buffer for 2 hours followed by primary antibody incubation, mouse anti-Zonula Occludens (ZO1-1A1, 1:200, ThermoFisher Scientific, Waltham, MA) and rabbit anti-GFP (1:200, ThermoFisher Scientific) at room temperature overnight. Incubation with secondary antibodies (Alexa Fluor 555 and 649, ThermoFisher) was performed at room temperature overnight, and the retina was flat-mounted on a glass slide. For retinal cross sections, affinity-purified rabbit polyclonal opsin antibodies, a gift from Dr. David R. Hyde [84], were used. Images were acquired with a Zeiss AxioImage ZI Epifluorescent Microscope (Carl Zeiss Microimaging, Thornwood, NY) equipped with an ApoTome attachment for optical sectioning structured illumination, Leica DM6000 Upright Microscope System (Leica Microsystems, Werzlarm Germany) and a Leica TCS SP5 confocal microscope equipped with Leica 40X HCX PL APO CS Oil Immersion lens.

### Generation of transgenic zebrafish with nuclear-localized photoconvertible (green-to-red) EOS protein expressed specifically in UV cones

Multi-Gateway-based *tol2* kit system was used to generate expression vectors [85]. In brief, the 5’ entry clone, *p5E*- *5*.*5sws1* [80], middle entry clone, *pME-nEOS* (gift from Dr. David Raible), and 3’ Entry clone, *p3E-polyA*, were assembled into a destination vector, *pDestTol2pA* [85] using LR Clonase II Plus enzyme (Thermo Fisher Scientific). Embryos of the transparent *ruby* genetic background [40] at the 1-cell stage were injected with 1 nL of solution containing 25 pg plasmid DNA and 25 pg tol2 transposase mRNA [86]. Founders (F0) with germline transmission of the transgene were identified by outcrossing with wildtype animals, and their F1 progenies were screened for nEOS expression at 4 days post fertilization.

### CRISPR-Cas9 mediated mutation in the *thrb* gene

A genetic mutation targeting the type 2 isoform of the *thrb* gene (synonym, *trß2*) was generated by CRISPR-Cas9 gene editing methods [87]. Briefly, pT7 gRNA vector (Addgene #46759) was used as a template to construct the *thrb2* gRNA [87]. PCR based method was performed using specific primers, 5’-GGGGTAATACGACTCACTATAGGCAACACAGCCAACCCTATGTTTTAGAGCTAGAAATAGCAAG-3’; 5’-AAAAAGCACCGACTCGGTGCC-3’. The MEGAscript T7 Kit (Ambion Inc., Austin, TX) was used to transcribe the gRNA. For the *nlsCas9nl* mRNA synthesis and purification, mMESSAGE mMACHINE T7 Transcription Kit (Ambion) and Qiagen RNeasy mini kit (Qiagen, Hilden, Germany) were used. For genotyping of the *thrb* mutation, PCR fragments of the *thrb* gene, amplified using specific primer set, 5’-CATGGTGTAAGTGGCGGATATG -3’; 5’-TCCACTGCATCTGAGAGAAATCC-3’, were subjected to restriction with BstXI (New England Biolabs, Ipswich, MA).

### nEOS photoconversion and imaging

Photoconversion of nEOS protein was performed on *ruby; Tg(sws1*:*nEos)* fish [88]. Juvenile zebrafish (0.7 to 0.88 cm standard body length) were anesthetized with 0.672 mg/ml Tricaine S/ MS-222 (Western Chemical Inc., Ferndale, WA) and placed dorsal side down on a 50 mm glass bottom petri dish with a No. 1.5 coverslip (MarTek Corporation, Ashlan MA, see [40]) and held in place with damped Kimwipes. Imaging and photoconversion were performed with a Leica TCS SP8 LSCM (Leica Microsystems, Werzlar, Germany) equipped with Leica 40X PL APO CS2 Water Immersion lens, 1.1 NA with 650 μm working distance. Green to red photoconversion of nEOS protein was performed by a 405 Diode laser at 400 Hz scan speed with a resolution of 512 x 512 pixels in the *xy* dimension at a single optical plane. Pre and post photoconversion images were captured with the White Light Laser tuned to 506 nm for nEOS (green) and 573 nm for nEOS (red). Leica HyD hybrid detectors were tuned to 516–525 nm for nEOS (green) and 620–761 nm nEOS (red).

### Large tile scans of flat-mounted retinae

Large tile scans of entire flat-mounted retinae from adult *Tg(sws1*:*EGFP)* zebrafish immunostained for ZO1 were acquired with a Leica TCS SP8 LSCM (Leica Microsystems) equipped with Leica 20X PL APO Dry lens. The GFP signal was recovered by immunostaining with anti-GFP antibody. The White Light Laser was tuned to 555 nm for Alexa Fluor 555 and 649 nm for Alexa Fluor 649. The Leica HyD hybrid detectors were tuned to 600–641 nm for Alexa Fluor 555 and 701–751 nm for Alexa Fluor 649. Images were acquired at 700 Hz scan speed with a resolution of 2048 x 2048 pixels in the *xy* dimension with a 2.0 μm interval between optical sections in the z-dimension.

### Row tracing of flat-mounted retinae

We manually traced rows of UV cones, starting near the region coinciding with the disorder-to-order transition (Figs 3A, 3B and S4 and Tables 1 and S1). The row tracing extends over approximately one hundred columns of UV cones from the larval remnant to the periphery, avoiding regions of the retinae that were damaged during flat mounting. Based on the row tracing, we identified where rows are inserted (*i*.*e*., Y-Junctions) and where rows are removed (*i*.*e*., reverse Y-Junctions, see S1 Table).

### Detection of grain boundaries in flat-mounted, row-traced retinae

In this section, we describe our method for identifying grain boundaries in images from experiments or simulations. In an infinite crystal, the rotation of the crystal axes in its vicinity is a clear signature of a grain boundary: The direction of these axes is unperturbed far from an isolated dislocation, but a finite linear density of dislocations must induce a rotation in the grain orientation. The situation is somewhat more ambiguous in our finite-sized, flat-mounted retinae (Figs 3A, 3B and S4). In particular, it is certainly possible to distribute Y-junctions roughly uniformly across the retina, such that the orientation of the rows in the crystalline cone mosaic rotates smoothly through 360 degrees as one goes around the pole of the retinal hemisphere. In such a situation, we would not say that grain boundaries are present. Instead, we would like to define a grain boundary as a region where the Y-junctions tend to coalesce into radially directed lines, or, equivalently, as one where the row orientation undergoes a rapid change. There is, however, no unique answer to the question of how large a jump in orientation over how short a distance is required for a grain boundary to be present. Instead, we will be forced to introduce cutoffs that, while corresponding reasonably well to our intuitive picture of what constitutes a grain boundary, are nonetheless somewhat arbitrary.

Specifically, in order to facilitate comparisons between experimental and simulation results, we would like to analyze our data in a way that tells us whether or not a given Y-Junction is part of a grain boundary. In the flat-mounted retinae, we have the positions of Y-Junctions, which generate row insertions, in the traced regions. In addition, individual rows are traced (meaning that each row has a distinct curve running along it). To define whether a given Y-junction belongs to a grain boundary, we check whether the row orientation of the surrounding domain rotates appreciably in the vicinity of the Y-Junction.

This method requires two arbitrary parameters:

- the length-scale over which we average the domain rotation (which we will call Δ*r*)
- how large the domain rotation must be for a defect to be “in a grain boundary”

We describe positions on the flattened retinae via polar coordinates (where the approximate center of the retina lies at the origin). For each Y-junction, we average the domain orientation in five distinct boxes (which we will use to compute how much the row orientation changes near the Y-Junction):

- The first box is centered on the Y-junction of interest (at radial coordinate *r* = *r*_0_ and angular coordinate *θ* = *θ*_0_). It runs from $r=r_{0}-\frac{\Delta r}{2}$ to $r=r_{0}+\frac{\Delta r}{2}$ and from $\theta =\theta_{0}-\frac{\frac{1}{2}\Delta r}{r_{0}}$ to $\theta =\theta_{0}+\frac{\frac{1}{2}\Delta r}{r_{0}}$.
- The second box runs from $r=r_{0}-3\frac{\Delta r}{2}$ to $r=r_{0}-\frac{\Delta r}{2}$ and from $\theta =\theta_{0}-\frac{\frac{1}{2}\Delta r}{r_{0}}$ to $\theta =\theta_{0}+\frac{\frac{1}{2}\Delta r}{r_{0}}$.
- The third box runs from $r=r_{0}+\frac{\Delta r}{2}$ to $r=r_{0}+3\frac{\Delta r}{2}$ and from $\theta =\theta_{0}-\frac{\frac{1}{2}\Delta r}{r_{0}}$ to $\theta =\theta_{0}+\frac{\frac{1}{2}\Delta r}{r_{0}}$.
- The fourth box runs from $r=r_{0}-\frac{\Delta r}{2}$ to $r=r_{0}+\frac{\Delta r}{2}$ and from $\theta =\theta_{0}-3\frac{\frac{1}{2}\Delta r}{r_{0}}$ to $\theta =\theta_{0}-\frac{\frac{1}{2}\Delta r}{r_{0}}$.
- The fifth box runs from $r=r_{0}-\frac{\Delta r}{2}$ to $r=r_{0}+\frac{\Delta r}{2}$ and from $\theta =\theta_{0}+\frac{\frac{1}{2}\Delta r}{r_{0}}$ to $\theta =\theta_{0}+3\frac{\frac{1}{2}\Delta r}{r_{0}}$.

To calculate the orientation within each box, we first identify all row lines that run through the box. For each row line (a skeletonized line composed of individual pixels), we calculate its orientation via principal component analysis:

- computing the covariance matrix of the row’s pixels’ positions within the box of interest (cov; MATLAB 2016B, MathWorks, Natick, MA)
- subsequently identifying the direction along which the row’s pixels’ positions vary the most (eig; MATLAB 2016B, MathWorks, Natick, MA)

By convention, row lines always run from the center of the retina to its periphery, so we encode each row’s orientation with a unit vector that runs along the row from center to periphery (rather than from periphery to center). Then, within the box of interest, we do a weighted average over the unit vectors (one for each row line within the box), where the weight of each row is simply the number of pixels of that row line that fall within the box. Then, we convert this weighted average of row vectors to a unit vector, which represent the row orientation within the box.

Now that we have the row orientation for each of the five boxes (unit vector ${\hat{u}}_{1}$ for box 1, …, unit vector ${\hat{u}}_{5}$ for box 5). We calculate how much the row orientation changes about the Y-Junction along the *θ* direction ($\Delta \phi_{45}=\arccos{(\hat{u}}_{4}\cdot {\hat{u}}_{5})$) as well as along the *r* direction ($\Delta \phi_{23}=\arccos{(\hat{u}}_{2}\cdot {\hat{u}}_{3})$). If the grain boundaries were purely radial, we could focus on Δ*ϕ*_45_ and ignore Δ*ϕ*_23_. In flat-mounted retina, because of retinal deformations due to cutting and flattening, grain boundaries are frequently not well-aligned with the nominal radial direction. Because of this, we compute $\Delta \phi_{rms}=\sqrt{{\left(\Delta \phi_{45}\right)}^{2}+{\left(\Delta \phi_{23}\right)}^{2}}.\;\Delta \phi_{rms}$ takes into account the change in row orientation in the vicinity of the Y-Junction in both directions.

To determine whether a Y-Junction is within a grain boundary or not, we compare its Δ*ϕ*_*rms*_ to some cutoff (for example, ten degrees or twelve degrees or fourteen degrees). If Δ*ϕ*_*rms*_ is greater than the chosen cutoff, the Y-Junction is in a grain boundary; if not, the Y-Junction is not in a grain boundary. Because by its very nature this cutoff is arbitrary, we vary this parameter (as well as Δ*r*) to see how the fraction of defects in grain boundaries changes with these parameters. We find that the fraction of Y-Junctions in grain boundaries depends very weakly on Δ*r*, but strongly on the cutoff in rotation of row orientation. To see if our simulations and experimental images have similar fractions of Y-Junctions in grain boundaries, we fix the values of these two parameters when comparing flat-mounted retinae to simulations.

We conclude this section by noting a possible pitfall of this method as applied to flat-mounted retinae: In regions where the row orientation is slightly warped due to deformation of the retina from the flattening process, Y-junctions may be designated as “in a grain boundary” (even if they are isolated) because of this artifactual deformation of row orientations. Thus, it is possible that this approach somewhat overestimates the fraction of Y-junctions in grain boundaries in flat-mount images compared to the fraction in simulations. (See below for an alternative method of defining grain boundaries that is expected to be less sensitive to distortion upon flattening.)

### Detection of grain boundaries in simulated cone mosaic

The basic method for detection of grain boundaries in simulated retinae is identical to that for flat-mounted (experimental) retinae with a couple of exceptions:

First, unlike for the experimental images, we do not trace rows by hand. Instead, we compute a Delaunay triangulation that connects peaks in the simulated particle density. Then, we identify “row” bonds based on which bonds are well-aligned with the local radial direction (that runs from the cone periphery to the cone center). More specifically, for each peak in the particle density, we:

1. compute the negative of the local radial direction ($-\hat{r}$)
2. compute a vector from this peak to each of its nearest neighbors in the Delaunay triangulation
3. compute the dot product of $-\hat{r}$ with each of the vectors to nearest neighbors

Then, whichever bond has the largest (positive) value of this dot product is a “row” bond. Note that based on this definition, a particle density peak associated with a Y-Junction ends up with three row bonds because it has two row bonds emanating from it (towards the periphery of the simulation domain) and one row bond flowing into it (from the center of the simulation domain). On the other hand, a reverse Y-Junction only has one row bond because no row bonds emanate from it to the periphery of the simulation domain.

After identifying row bonds in the Delaunay triangulation, to calculate the row orientation within a given box, we find row bonds that fall entirely within the box. We compute a weighted average over these row bonds, represented as vectors that run (mostly) from center of simulated retina to its periphery; each weight is equal to the length of the corresponding row bond.

### Alternative method for detection of grain boundaries

The definition of grain boundaries described in the preceding sections and used in the main text has the advantages that it connects directly to the basic definition of a grain boundary as the interface between two domains with different crystallographic orientations and that it depends on only two parameters, both with clear biophysical interpretations. As we noted above, however, when it is applied to images of flat-mounted retinae, it is potentially susceptible to systematic biases caused by distortions introduced by the mounting and fixation process. To verify that these biases do not dominate our results, in this section we consider an alternative method of defining grain boundaries that views the boundaries as collections of Y-junctions forming a (nearly) straight line. Because it does not depend as strongly on the local row orientation, this method is more robust against artifacts like deformation of flat-mounted tissue. This robustness, however, comes with the tradeoff that the method requires setting several *ad hoc* parameters that lack clear and appealing interpretations.

To define grain boundaries, we search for approximately linear chains of five Y-Junctions, which are nearest neighbors. To the image of all Y-Junctions in the traced regions, we apply the following algorithm:

1. Loop through all Y-Junctions one-by-one. We will build a chain of nearest neighbors, of five Y-Junctions, for each Y-Junction.
  - Look for the Y-Junction’s nearest neighboring Y-Junction, using a k-nearest neighbors search (knnsearch; MATLAB 2016B, MathWorks, Natick, MA). Add the nearest neighbor to the chain.
  - For that nearest neighbor, add its nearest neighbor, excluding any Y-Junctions that already belong to the chain.
  - Repeat b until you have a chain of five Y-Junctions, including the Y-Junction that initialized the chain.
2. Now, based on the calculation in step 1, every Y-Junction, indexed by *i* below, has a chain of nearest neighbors, including the Y-Junction itself. We index the five defects in the chain by *j*. The position of the *j*^th^ defect in the *i*^th^ chain is $\vec{r_{i,j}}$. For each Y-Junction, we compute the following sum:
  $$a_{i}=\frac{1}{4}\sum_{j=1}^{4}\frac{\left(\vec{r_{i,1}}-\vec{r_{i,5}}\right)}{\left|\vec{r_{i,1}}-\vec{r_{i,5}}\right|}∙\frac{\left(\vec{r_{i,j}}-\vec{r_{i,j+1}}\right)}{\left|\vec{r_{i,j}}-\vec{r_{i,j+1}}\right|}.$$
3. Now, based on the calculations in steps 1 and 2, every Y-Junction, indexed by *i*, has a chain of nearest neighbors, which is assigned a score *a*_*i*_. If *a*_*i*_ = 1, the chain of five Y-Junctions is perfectly linear. If *a*_*i*_>*a*_*gb*_, we call the *i*^th^ chain a grain boundary. We set the cutoff *a*_*gb*_ equal to seven-eighths.
4. We initialize an empty array, in which we will store Y-Junctions that belong to grain boundaries. Loop through all Y-Junctions, indexed by *i*, one-by-one.
  - If *a*_*i*_>*a*_*gb*_, store (*i*.*e*., in the array of all Y-Junctions in grain boundaries) the five Y-Junctions which belong to this chain.

We also perform this computation (steps 1–4) on the Y-Junctions in simulations of the phase-field crystal model. In that case, we map the cone frustum to the flat plane. This does not generate distortions because cones are isometric to the plane. We, then, calculate which dislocations form grain boundaries, respecting the periodic boundary conditions of the flattened cone frustum. To compare the experimental results to simulations, see S6 Table and S12 Fig.

The fraction of defects in grain boundaries, as quantified by this alternative method that we applied to the flat-mounted retinae, is approximately sixty percent (S12 Fig). In the eight flat-mounted retinae, approximately fifty percent of Y-Junctions are in grain boundaries (S6 Table).

### Tracking nuclear positions in photoconverted regions

In the photoconversion experiments, we observe the same region of the same retina at two different times in live fish. Given a nucleus at one time point, we want to find the same nucleus in the image at the other time point. One image of the region is taken immediately after photoconversion, which we call day 0. Across fish, we vary the time between photoconversion and the time of the second observation (*i*.*e*., two days after photoconversion at the earliest and four days after photoconversion at the latest). We call the second time point day 2–4.

At both times of observation for each fish, we have an image with two channels. One channel corresponds to the color of the photoconverted fluorescent protein. The other channel corresponds to the color of the non-photoconverted fluorescent protein. For the image analysis below, we use the photoconverted channel at both times. The image is three-dimensional, and the plane which contains the UV cone nuclei (*i*.*e*., where the fluorescent protein is localized) is mostly parallel to the x-y plane. This fact allows us to perform most of the computations, for tracking each nucleus from one image to the other, based on two-dimensional projections.

For each z-stack, we compute a two-dimensional wiener filter (wiener2; MATLAB 2016B Image Processing Toolbox, MathWorks) with a filter size of eight pixels, which is approximately a micron. This filter removes noisy specks (*i*.*e*., spikes in intensity at small length scales). We, then, compute a two-dimensional projection by summing over z-stacks. The photoconverted UV cones are in the middle of the image. The intensity in the photoconverted channel is significantly weaker for UV cones near the edge of the image. This provides us the reference boundary by which we can identify common nuclei (*i*.*e*., which nucleus in the day 2–4 image corresponds to a specific nucleus in the day 0 image).

We perform an image registration, computing the combination of rotation and translation that optimizes the normalized cross-correlation between the two images (normxcorr2; MATLAB 2016B Image Processing Toolbox, MathWorks). Then, we segment nuclei in the two images. Because the intensity of UV cone nuclei varies significantly across the image, we use both adaptive thresholding (adaptthresh; MATLAB 2016B Image Processing Toolbox, MathWorks) and a low absolute threshold. We morphologically open the thresholded image, followed by morphological closing. We fill holes in the image (imfill; MATLAB 2016B Image Processing Toolbox, MathWorks) and clear the border of the image (imclearborder; MATLAB 2016B Image Processing Toolbox, MathWorks). We perform minimal manual correction of these segmentations. Given that we have aligned the two images and segmented the nuclei, we track each nucleus from one image to the other by computing for each nucleus in the day 0 image its nearest neighbor in the day 2–4 image (knnsearch; MATLAB 2016B, MathWorks). As a sanity check, for each nucleus in the day 2–4 image we compute its nearest neighbor in the day 0 image to make sure that this calculation returns, for each nucleus, an answer consistent with the reverse calculation. We manually correct any errors.

Following this segmentation and identification of common nuclei between the two images, we want to estimate the three-dimensional position of each nucleus based on the raw z-stacks rather than on a post-processed version. We identify a circular region, of radius two and a half microns, in the xy-plane centered on each of the segmented nuclei. This radius is larger in the xy-plane than the nuclear radius but small enough not to encompass other nuclei. This circular region corresponds to a pillar in the z-direction. To estimate the three-dimensional position of each nucleus in both images, we use the raw z-stacks, computing the center of intensity of each pillar (*i*.*e*., weighted average of voxel positions in each pillar where the weights are the voxel intensities). At the end of this entire procedure, for each nucleus common to both images, we know its position at both time points.

### Tracking UV cone positions in photoconverted regions and measuring glide motion

To identify the location of the Y-Junction, we need to calculate a triangulation over the nuclear positions. At both day 0 and at day 2–4, the UV cone nuclei positions in each experiment are well fit by a plane, which we fit by simple least-squares minimization (see RMSE information in S3 Table). For calculating the triangulation, we project the UV cone positions onto the plane of best fit. We, then, calculate the triangulation in that plane (delaunayTriangulation; MATLAB 2016B, MathWorks).

We want to track movement of UV cones near the Y-Junction core along the direction of glide motion. We systematically search for bond flips (*i*.*e*., any change in nearest neighbor assignments as in Fig 5C and 5C’) between day 0 and day 2–4 for any bonds that could be flipped in glide motion (see Fig 4C). Which UV cone bonds lie along the glide line is always unambiguous based on the triangulation. We never observe glide motion by more than one row, as illustrated in Figs 4C and 5A. We show an experimental example of glide motion by one row in Fig 5C and 5C’ (see S3 Table).

### Statistical significance of growing grain boundaries in live fish

In S6 Fig, we show examples of images in which we can identify newly incorporated Y-Junctions lining up into grain boundaries. These are images of UV cone nuclei near the retinal margin (*i*.*e*., where the layer grows by addition of post-mitotic cells). These images are oriented such that the margin is parallel to the y-axis. Our field of view in these images contains approximately forty rows of UV cones and forty columns of UV cones.

To identify grain boundaries that are already visible immediately after photoconversion, we trace rows of UV cones at the retinal margin. If immediately after photoconversion the row direction rotates about a group of defects by ten degrees or more at the margin, we call the group of Y-Junctions in-between the rotated rows a grain boundary. (S4 Table also lists results for analyses where this threshold is instead taken to be twelve or fourteen degrees.) As a justification for this use of row rotation to identify grain boundaries, see Figs 3C, 3D, 5D, 5D’ and S6. For a cutoff of twelve degrees, out of the eighteen samples, nine samples have grain boundaries. Since some samples have two grain boundaries, in total we observe eleven grain boundaries above the twelve-degree cutoff in live fish (Table 2).

All subsequent analysis is based on the later image (*i*.*e*., two, three, or four days later). We trace rows in the later image. We identify newly inserted rows (*i*.*e*., newly incorporated Y-Junctions) in the later image, and we again identify the old defects within each grain boundary (*i*.*e*., those not newly incorporated). We calculate a one-dimensional coordinate for the location of each grain boundary in the later image. This one-dimensional coordinate is the average of y-coordinates (*i*.*e*., axis approximately parallel to the margin in the image) of all defects (*i*.*e*., not newly incorporated) within each grain boundary. We are interested in how close, along the y-direction, newly incorporated Y-Junctions are to the nearest grain boundary in the image.

Suppose that in the image there is only one grain boundary that is identifiable at the time of photoconversion and later imaging. Suppose this grain boundary is located at coordinate *y*_*gb*_. The image spans from *y* = 0 to *y* = *y*_*max*_. For each new Y-Junction, we generate one hundred thousand random Y-Junction positions, uniformly distributed from *y* = 0 to *y* = *y*_*max*_ (rand; MATLAB 2016B, MathWorks). We calculate the distance between each of these one hundred thousand random Y-Junction positions and the grain boundary (at *y*_*gb*_). We call the array of distances between each random Y-Junction position and the grain boundary $\vec{\delta_{rand}}$. We also store the actual distance, which we call *δ*_*actual*_, between each observed newly incorporated Y-Junction in the image and the nearest grain boundary in the image.

Suppose that in the image there are two grain boundaries that are identifiable at the time of photoconversion and later imaging. Suppose their coordinates are *y*_*gb*,1_ and *y*_*gb*,2_. The image spans from *y* = 0 to *y* = *y*_*max*_. For each new Y-Junction, we generate one hundred thousand random Y-Junction positions, uniformly distributed from *y* = 0 to *y* = *y*_*max*_ (rand; MATLAB 2016B, MathWorks). We calculate the distance between each of these one hundred thousand random Y-Junction positions and the nearest grain boundary (at either *y*_*gb*,1_ or *y*_*gb*,2_). We call the array of distances between each random Y-Junction position and the nearest grain boundary $\vec{\delta_{rand}}$. We also store the actual distance, *δ*_*actual*_, between each newly incorporated Y-Junction and its nearest grain boundary.

Based on the procedure outlined above, for each new Y-Junction, we have a vector of length one hundred thousand and a scalar, $\vec{\delta_{rand}}$ and *δ*_*actual*_, respectively (see S4 Table). If after random incorporation with respect to the grain boundaries in the image, a newly incorporated Y-Junction moves at a speed of one row per two days closer to the nearest grain boundary (with spacing between rows approximately equal to six microns as shown in S2 Fig), the distribution of distances with respect to the nearest grain boundary becomes $\max\left(\vec{\delta_{rand}}-3\frac{\mu m}{day}\Delta t*\vec{1},\vec{0}\right)$, where Δ*t* is the time between photoconversion and later imaging (*i*.*e*., two, three, or four days).

Depending on which cutoff we use to define a grain boundary (S4 Table), we have as many as thirty-seven new Y-Junctions across twelve samples (with at least one grain boundary at the retinal margin). We would like to compare these scalar values of *δ*_*actual*_ to a concatenated vector of $\max\left(\vec{\delta_{rand}}-3\frac{\mu m}{day}\Delta t*\vec{1},\vec{0}\right)$ across the newly incorporated defects. This concatenated vector has a length that is one hundred thousand times as long as *δ*_*actual*_. We test whether the distribution of *δ*_*actual*_ has the same median as the concatenated vector of $\max\left(\vec{\delta_{rand}}-3\frac{\mu m}{day}\Delta t*\vec{1},\vec{0}\right)$. We assign a p-value to that comparison via Mann-Whitney U-test (ranksum; MATLAB 2016B Statistics and Machine Learning Toolbox, MathWorks).

### Numerical solutions of lateral inhibition on disordered cell packing

Starting with a Voronoi tessellation of uniformly (randomly) distributed points, we generated large, disordered, periodic cell packings (*e*.*g*., 20,000 total cells in S11 Fig) via vertex model simulations with equal tensions on all edges as described in [89,90]. We model dynamics of individual cell fates on the static cell packing according to the model described in [27], but do not include noise in the dynamics (*D* = 0). Since we changed some aspects of the model, including the external signaling gradient and the noise in fate, we describe the model in [27] below for the sake of clarity. The fate of cell *i*, *u*_*i*_, evolves as:

$$\tau \frac{du_{i}}{dt}=f(u_{i},s_{i})-u_{i}$$

where *s*_*i*_ is the signal each cell receives from other cells as well as from any external gradients. We interpret the *u* = 1 fate as the UV cone spectral subtype and the *u* = 0 fate to be other spectral subtypes. Also, *f*(*u*, *s*) is sigmoidal: $f(u,s)\equiv f(u-s)=\sigma [2(u-s)]=\frac{\left(1+\tanh\left(2(2(u-s))\right)\right)}{2}$.

The signal that cell *i* receives, *s*_*i*_, includes an external, time-dependent signal *s*_0_(*x*, *t*) as well as signals from neighboring cells in a distance-dependent manner.

$$s_{i}=s_{0}(x_{i},t)+\sum_{j}c_{ij}D^{*}(u_{j})$$

The external signal provided to the cells has the following form: $s_{0}(x,t)=S_{0}\sigma \left(\frac{x-vt}{\epsilon \sqrt{A_{0}}}\right)\equiv S_{0}\left(\frac{1+\tanh\left(\frac{2(x-vt)}{\epsilon \sqrt{A_{0}}}\right)}{2}\right)$ where *S*_0_ = 1 and $v=\frac{l}{4\tau }$ and $\epsilon =\frac{1}{50}$. *τ* is the timescale for cell fate dynamics, and *l* is the characteristic cell-cell signaling range. The distance-dependent coupling constant *c*_*ij*_ between cell *i* and cell *j* is of the form: $c_{ij}=e^{-{d_{ij}}^{2}/(2l^{2})}$, where *d*_*ij*_ is the distance between the centroids of cell *i* and cell *j*. No cell signals to itself directly: *c*_*ii*_ = 0.

A cell of fate *u*_*j*_ produces signal D*(*u*_*j*_) = *a*(*u*_*j*_)*D*(*u*_*j*_). The ligand level of cell *j*, *D*(*u*_*j*_), is directly proportional to the fate *u*_*j*_. The ligand activity of cell *j*, called *a*(*u*_*j*_), is of the form: $a(u_{j})=a_{0}+\frac{3u^{3}}{1+u^{2}}a_{1}$. The constants *a*_0_ and *a*_1_ are 0.05 and 0.95, respectively.

To explore the effects of cell-cell signaling range on the final fate pattern, we systematically change the signaling range *l* from $l=3.0\sqrt{A_{0}}$ to $l=1.75\sqrt{A_{0}}$ to $l=\sqrt{A_{0}}$, where *A*_0_ is the mean cell area. All cells are initially in the *u* = 0 state. The sigmoidal signaling front, sharper than the characteristic cell size, starts at left side of the packing (*x* = 0 at *t* = 0) and moves to the right. In the wake of the front, individual cells differentiate into the *u*≈1 fate, inhibiting their neighbors from adopting the *u*≈1 fate within the specified cell-cell signaling range. We solve the differential equations for cell fates using ode45 (MATLAB 2016B, MathWorks).

### Numerical solutions of anisotropic phase-field crystal model on cone

The free energy functional *F* for an anisotropic phase-field *ψ*, with undercooling parameter *R*, is an integral over the simulation domain [61,68,91]:

$$F=\int (\frac{1}{2}\psi [R+{\left(1+\nabla_{s}^{2}\right)}^{2}]\psi +\frac{\psi^{4}}{4})d\vec{r}$$

$$\nabla_{s}^{2}=b^{2}\frac{\partial^{2}}{{\partial x}^{2}}+\frac{1}{b^{2}}\frac{\partial^{2}}{{\partial y}^{2}};b>1.$$

(stretched along the *x* direction)

$$\nabla_{s}^{2}=b^{2}\frac{1}{r}\frac{\partial }{\partial r}\left(r\frac{\partial }{\partial r}\right)+\frac{1}{b^{2}}\frac{1}{r^{2}}\frac{\partial^{2}}{{\partial \theta }^{2}};b>1.$$

(stretched along the *r* direction in polar coordinates)

The particle-density-modulation field *ψ* is evolved to minimize the free energy functional *F* while conserving the mean of the field ($\psi_{0}=\int \psi d\vec{r}/\int d\vec{r}$):

$$\frac{\partial \psi }{\partial t}=\nabla^{2}([R+{\left(1+\nabla_{s}^{2}\right)}^{2}]\psi +\psi^{3})$$

For solving this equation on the cone, we first map the cone to a flat plane, which does not generate distortions because the cone is isometric to the plane. We, then, use the Laplacian for polar coordinates, respecting the periodic boundary condition of the cone. We set up the problem in terms of the variables *u*, *v*, *ψ* as defined in [92]. For computational efficiency, we take the Fourier transform along any direction which is periodic (*e*.*g*., along the *θ* direction on the cone). We use first-order implicit-explicit methods, as in [93], treating the non-linear term in *ψ* explicitly. We implement all derivatives by finite differences [94]. We use no-flux boundary conditions at each non-periodic boundary.

We evolve the system with a fixed step size in time (Δ*t* = 0.075). The computational grid is such that there are approximately 25 grid points per lattice spacing along the circumferential direction in the initial column, and approximately 10 grid points per lattice spacing along the radial direction.

We systematically vary the parameters of the phase-field crystal model as done in [61]; note that stretching the crystal does not change the phase diagram as discussed in [64]. In short, we take a 1D cut of the phase diagram, setting $\psi_{0}=-\frac{\sqrt{-R}}{2}$ as we vary the undercooling parameter *R*. For this cut, we also vary the strength of the noise in the initial conditions. These three parameters are the parameters on which we do not have any quantitative handle (relative to experiments); therefore, we perform this robustness analysis on these three parameters. The results of this analysis are shown in S8 Fig.

### Geometry for cone mosaic growth

The retina is approximately hemispheric. A hemisphere might, thus, seem like the most obvious choice of geometry in which to test cone mosaic growth. It is important to note, however, that the retina is not a hemisphere of a fixed radius during development. Its radius increases as new retinal cells are incorporated. As the hemisphere dilates, the existing cone photoreceptor layer must be deformed. The exact way in which the existing pattern deforms, and how that affects subsequent cone mosaic formation, is beyond the scope of this paper. Our aim is to choose a minimal geometry which allows us to test the phase-field crystal model’s ability to form grain boundaries.

We choose a geometry in which we can easily tune the defect density. We choose a cone frustum, which is constructed by slicing off the top of a cone with a plane that is parallel to its base. By changing the level at which we slice the cone, we tune the number of UV cones in the initial column. We choose the top level such that there are approximately two hundred initial rows, which is consistent with the number of initial rows identified in flat-mounted retinae. By changing the opening angle of the cone, we can tune the number of Y-Junctions required to maintain constant cell-cell spacing per added column. We choose an opening angle such that two row insertions are required per added column to maintain approximately constant cell-cell spacing. The number of Y-Junctions necessary to maintain constant cell-cell spacing is comparable to the number of Y-Junctions observed in the retinae (Table 1 and S4 Fig).

### Initial conditions for cone mosaic growth

At the very top level of the cone frustum, we lay down one column of cones (see one-mode approximation in [61]). We add a white-noise mask to this initial column of cones. Because we do not have any quantitative handle on the noise in cell positions at the onset of patterning in the zebrafish retina, we vary the noise strength, exploring its effect on the subsequent pattern of defects (S8 Fig and S5 Table).

## Supporting information

S1 Fig Defects other than Y-Junctions observed in live fish. (A) In this image, the nuclear-localized, photoconverted protein in UV cones is pseudo-colored magenta. White bonds: triangulation connecting nearest neighbors. The seven- and five- coordinated UV cones: seven- and five-sided stars, respectively. A Y-Junction exists near the reverse Y-Junction. Gray oval encloses the reverse Y-Junction. Row counts are annotated on each side of the image. (B) Example of double-row insertion near a standard Y-Junction. The double-row insertion, enclosed by the gray oval, corresponds to a five- and seven-coordinated particle that are not directly connected by a bond in the lattice. Note that this double-row insertion does not disrupt the patterning of the cone mosaic. Row counts are annotated on each side of the image.
(TIF)

S2 Fig In live-imaging experiments, we quantify anisotropy of the UV cone triangular lattice. (A) Patch of photoconverted UV cones near the retinal margin. This patch of UV cones, that express nuclear-localized fluorescent protein, does not contain a Y-Junction. We use this patch, and two others, to quantify spacing between UV cones. (B) Triangulation for the patch of UV cones in panel A. In this triangulation, bonds connecting UV cones in the same row are black lines. Bonds along the other two principal directions of the lattice are blue and red (not at all related to Blue and Red cones). (C) Scatter plot of bond length versus bond orientation in triangulation from panel B. The same color scheme denotes bonds along the row direction and along the two other principal directions. p-values calculated via Mann-Whitney U-test. (D-E) Equivalent of panel C for two other samples. (F) The column direction is NOT a principal direction in the triangular lattice, meaning that UV cones in the same column are not each other’s nearest neighbors. Using a section of the triangulation from panel B, we illustrate spacing along the row direction by black arrows, and the spacing along the column direction by gray arrows. For an isotropic lattice, the column spacing is a square root of three times the row spacing. For this lattice, we can calculate the column spacing, given mean bond lengths in the three principal directions. We find that the column spacing is approximately twelve and a quarter microns, as compared to a row spacing of approximately ten and a quarter microns. This column to row spacing ratio is less than the square root of three, meaning that row bonds are elongated relative to the case of an isotropic lattice.
(TIF)

S3 Fig Distribution of Red and Green cones near Y-Junction core. (A) Two Y-Junctions (asterisks) in a flat-mount retinal preparation from an adult, triple transgenic (Tg[*sws2*:*GFP*; *trβ2*:*tdTomato*; *gnat2*:*CFP*]) fish. Blue cones express a fluorescent reporter (pseudo-colored blue) under control of the Blue opsin promoter *sws2*, and Red cones express a fluorescent reporter (pseudo-colored red) under control of the *trβ2* promoter. All cones express an additional fluorescent reporter under control of the *gnat2* promoter. Although UV and Green cones do not express different fluorescent reporters, these two cone subtypes are morphologically distinguishable. (B) Nodes of graph are Red cones from panel A, and edges connect nearest neighbors in honeycomb lattice. Note the existence of a heptagon-pentagon pair (*i*.*e*., a ‘glide’ dislocation) in both defect cores. (C) Nodes of the graph are Green cones from panel A, and edges connect nearest neighbors in honeycomb lattice. Note the existence of an octagon (*i*.*e*., a ‘shuffle’ dislocation) in both defect cores. (D-F) Another example of a Y-Junction from a flat-mount retinal preparation from the same triple transgenic line (akin to panels A-C).
(TIF)

S4 Fig Algorithm for identification of grain boundaries. (A-C) For each of three flat-mounted retinae, image on the left-hand side is of all identified Y-junctions (yellow dots). Central image is all Y-junctions that our algorithm identified as in a grain boundary (with cutoff of twelve degrees). Image on the right-hand side is all Y-junctions that our algorithm identified as in a grain boundary (with cutoff of fourteen degrees). Panel A is fish 5 (total number of Y-Junctions = 249). Panel B is fish 4 (total number of Y-Junctions = 275). Panel C is fish 8 (total number of Y-Junctions = 285), the retina in Figs 3 and 6. See S2 Table.
(TIF)

S5 Fig Climb motion requires the creation or annihilation of vacancies or interstitials, which have no analog in the cone mosaic. (A) The creation of a vacancy allows a dislocation to climb (*i*.*e*., move perpendicular to the Burgers vector). The lattice in panel 1 has a dislocation. Photoconverted UV cones are magenta, and non-photoconverted UV cones are yellow. Panel 2 is triangulation in panel 1 with a new vacancy. Gray arrow is where the vacancy will hop, to create the distribution in Panel 3. Gray arrow in Panel 3 is where the vacancy will hop, to create distribution in Panel 4. As the vacancy hops, the defect core moves perpendicular to the Burgers vector. (B) Vacancy in the cone mosaic (two missing Red cones, two missing Green cones, one missing Blue cone, and one missing UV cone) can be destroyed. Red cone in Panel 1 must move as indicated by gray arrow to create distribution in Panel 2. Movements denoted by gray arrows in Panel 2 allow for the vacancy to close and for the defect to move. Panel 3 corresponds to the distribution of cones after destruction of the vacancy. We never observe a vacancy (involving two missing Red cones, two missing Green cones, one missing Blue cone, and one missing UV cone) in the cone mosaic, and thus consider climb motion irrelevant for our system.
(TIF)

S6 Fig Examples of photoconverted retinae with grain boundary growth during initial cone mosaic formation. (A) We trace rows of UV cones (white dashed lines) near Y-Junctions. Yellow dots: Y-Junctions observed immediately after photoconversion. White dots: Y-Junctions that are incorporated during the two days after photoconversion. Double-sided black arrow: newly incorporated UV cone columns. This is grain boundary 1 in Table 2. (B) Grain boundaries 4–1 and 4–2 in Table 2. All row tracing and Y-Junctions denoted in same way as in panel A.
(PDF)

S7 Fig Mutation in the *trβ2* gene deletes Red cones, but not other cone subtypes. (A) Immunocytochemistry for cone-subtype-specific opsins, including Red opsin (red), Green opsin (green), Blue opsin (blue), and UV opsin (yellow) in wild-type and *trβ2* mutant retinae. (B) Flat-mount retinal preparation of *trβ2* mutant immunostained for ZO1 (green). Profiles of UV cone are large and rounded (see Fig 6C and 6D). White dashed lines: some rows of UV cones.
(PDF)

S8 FigScanning Parameters of Phase-Field Crystal Model.We take a one-dimensional cut of the two-dimensional phase diagram of the phase-field crystal model ($\psi_{0}=\frac{-\sqrt{-R}}{2}$), where *ψ*_0_ is the mean of the density modulation field and where *R* is the undercooling parameter. The number of initial rows on the cone frustum is two hundred. Approximately ninety-five columns exist from the top of the cone frustum to the bottom. About two row insertions per added column are necessary to maintain constant cell-cell spacing. The degree of anisotropy is constrained by S2 Fig. We assume that the row orientation must rotate by twelve degrees or more at the site of a Y-Junction for that Y-Junction to be in a grain boundary (see S5 Table for the results of changing of this rotation parameter). (A) Standard deviation of white noise field, added to the first two columns, in these simulations is three-quarters. Along the one-dimensional cut of the phase diagram, we measure the fraction of Y-Junctions in grain boundaries. (B) The standard deviation of the white noise field in these simulations is one. Along the one-dimensional cut of the phase diagram, we measure the fraction of Y-Junctions in grain boundaries. (C) The standard deviation of the white noise field in these simulations is five-quarters. Along the one-dimensional cut of the phase diagram, we measure the fraction of Y-Junctions in grain boundaries. (D) For the same simulations in panel A, we plot the number of Y-Junctions. (E) For same simulations in panel B, we plot the number of Y-Junctions. (F) For same simulations in panel C, we plot the number of Y-Junctions.(TIF)

S9 Fig Application of grain boundary detection algorithm to simulation from Fig 7D. (A) Positions of all Y-Junctions (yellow dots). (B) Positions of all Y-Junctions (yellow dots) which coincide with a row orientation change of more than ten degrees. (C) Positions of all Y-Junctions (yellow dots) which coincide with a row orientation change of more than twelve degrees. (D) Positions of all Y-Junctions (yellow dots) which coincide with a row orientation change of more than fourteen degrees. See Detection of grain boundaries in simulated cone mosaic.
(TIF)

S10 Fig Additional insights generated by phase-field crystal model of cone mosaic formation. (A) Example of isotropic crystal growth on cone frustum with an initial column as prepattern. Yellow dots: seven-coordinated particles (including, but not limited to, those contained in Y-Junctions). Note the lines of seven-coordinated particles that do not radiate from the center of the cone to the periphery (example within red oval). These non-radiating lines of seven-coordinated particles result from a rotation of crystallographic orientation during growth of the isotropic crystal, not observed in zebrafish retinae. (B) Example of anisotropic crystal growth on cone frustum with no initial column as prepattern. With only white noise at the top of the cone in initial conditions, the anisotropy of the crystal (*i*.*e*., in the phase-field crystal free energy) selects and maintains the orientation during growth (in contrast with panel A). Even when a domain forms with improper orientation (example within red oval), the domain rotates to the proper orientation during growth. (C) Zoomed-in snapshot of an anisotropic phase-field crystal simulation on the surface of a cone. Note that near grain boundaries (*i*.*e*., where the domain rotation rotates), there is a lag in proper positioning of UV cones (*i*.*e*., density field remains poorly resolved) relative to growth of neighboring domains. This results in a characteristic V-shape.
(TIF)

S11 FigLateral inhibition, with varying signaling ranges, in a disordered cell packing.Triangular lattice of *u*≈1 cells forms on a square packing of 20000 cells with periodic boundary conditions. Defects (*i*.*e*., seven-coordinated) in triangular lattice of *u*≈1 cells are yellow dots. Initially, all cells are in state (*u* = 0) because an external inhibiting signal is provided to all cells. Starting at *t* = 0, a wave of de-inhibition moves from left to right (see Numerical solutions of lateral inhibition on disordered cell packing). The wave moves at a speed $v=\frac{l}{4\tau }$ where *τ* is the timescale of cell differentiation, and *l* is the range of cell-cell signaling. In each panel, the black arrow is the direction of wave propagation. (A) The signaling range is $3\sqrt{A_{0}}$, where *A*_0_ is the mean cell area. This signaling range results in seven to eight *u*≈0 cells between each pair of neighboring *u*≈1 cells in the final pattern. Note that some defects are generated early in pattern formation (*i*.*e*., left side of packing), but the right side of the packing contains no defects. (B) The signaling range is $1.75\sqrt{A_{0}}$. This results in about five *u*≈0 cells between each pair of neighboring *u*≈1 cells in the final pattern. The entire packing contains defects. (C) The signaling range is $1\sqrt{A_{0}}$, comparable to lattice spacing of the cone mosaic. This results in one to two *u*≈0 cells between each pair of neighboring *u*≈1 cells in the final pattern. The entire packing contains defects. This image is enlarged relative to panels A and B for the sake of clarity.(PDF)

S12 Fig Percentage of Y-Junctions in grain boundaries (as quantified by an alternative grain boundary detection method) in our simulations. The simulations analyzed here are the same simulations as in S8 Fig. Instead of testing for the rotation of row orientation in the vicinity of a Y-Junction, here we search for Y-Junctions that are approximately linearly aligned with other Y-Junctions that are their nearest neighbors (see Alternative method for detection of grain boundaries). (A) Standard deviation of the white noise field, added to the first two columns, in these simulations is three-quarters. (B) Standard deviation of the white noise field in these simulations is one. (C) Standard deviation of the white noise field in these simulations is five-quarters.
(TIF)

S1 Table Counts of reverse Y-Junctions (*i*.*e*., row deletions) in regions of the retinae in which we traced rows. The same fish numbers are used in Table 1. *There is a large-angle grain boundary in this retina, where patterning of the cone mosaic is slightly disrupted. There are potentially 10 additional reverse Y-Junctions associated with that large-angle grain boundary.
(PDF)

S2 Table Application of grain boundary detection to flat-mounted retinae (varying two arbitrary parameters). See Detection of grain boundaries in flat-mounted, row-traced retinae. Note that the percentage of Y-Junctions in grain boundaries depends strongly on the cutoff on the row orientation’s change; this threshold determines whether or not a Y-Junction is within a grain boundary. The percentage of Y-Junctions in grain boundaries depends only weakly on the size of the boxes over which we average row orientation (to calculate the row orientation’s change near the Y-Junction).
(PDF)

S3 Table Motion of defects within photoconverted regions. Here we quantify the motion of Y-Junctions within photoconverted regions. The fish labels are the same as in Table 2. Note that some fish are missing from this list (*e*.*g*., 4 and 8). These two samples have grain boundaries in neighboring non-photoconverted regions, but do not have defects within the photoconverted region itself. For each sample, we fit the positions of UV cone nuclei in the photoconverted region to a plane in order to compute a triangulation. Because the photoconverted region is small relative to the radius of curvature of the retina, the positions of UV cone nuclei are well fit (as quantified by RMSE) by a plane at both imaging times. In the UV cone triangulations, we check for bond flips near the defect core between photoconversion and later imaging (see Tracking UV cone positions in photoconverted regions and measuring glide motion). If a Y-Junction glides by one row, we denote that with a 1, and if a Y-Junction does not glide, we denote that with a 0.
(PDF)

S4 Table New Y-Junctions are incorporated preferentially near existing grain boundaries in live fish. The fish labels are the same as in Table 2 and S6 Fig. We first list the number of Y-Junctions incorporated between photoconversion and later imaging (within the whole image, not just within the grain boundary). We list the average and median distances (*i*.*e*., along the margin axis) between a new Y-Junction and its nearest grain boundary if each new Y-Junction’s position is uncorrelated with positions of existing grain boundaries. We, then, list the actual distances (*i*.*e*., along the margin axis) between each newly incorporated Y-Junction and its nearest existing grain boundary in the image. Fish 8 is omitted because, within the image, no new Y-Junctions are added between photoconversion and later imaging. Note that whether a collection of defects is a grain boundary or not depends on how strongly the row orientation changes from one side of that collection of defects to the other side. We systematically change the cutoff in the angle of rotation of the row orientation that defines grain boundaries to see how our results depend on that cutoff.
(PDF)

S5 Table Application of grain boundary detection to simulation data (varying two arbitrary parameters of detection algorithm). We group the simulations into distinct tables based on the value of the undercooling parameter *R*. Within each table, the noise in the initial conditions is varied. Note that each simulation has a unique ID, which we list in the same column as the simulation parameters. For each simulation, we list the total number of Y-Junctions and the number of Y-Junctions in grain boundaries. We find that the results depend weakly on the size of the boxes over which we average row orientation (see Detection of grain boundaries in simulated cone mosaic) but depend strongly on the cutoff in row orientation used to define whether a Y-Junction is in a grain boundary.
(PDF)

S6 Table Application of alternative method for grain boundary detection to flat-mounted retinae. See Alternative method for detection of grain boundaries for a description of the alternative method for grain boundary detection. These retinae correspond to the same retinae in Table 1. We show the percentage of retinal area analyzed, the number of Y-Junctions identified, and the percentage of Y-Junctions in grain boundaries according to the alternative method (based on linear alignment of Y-Junctions).
(PDF)

S1 Video Formation of anisotropic triangular lattice in a rectangular domain. The top and bottom (y) boundaries are periodic; the left and right (x) boundaries are no-flux. There are one hundred rows and one hundred columns.
(ZIP)

S2 Video Formation of anisotropic triangular lattice on a cone frustum with a couple of initial columns (masked with white noise). The cone is mapped to a flat plane here. The inner boundary (closest to origin) and the outer boundary (farthest from origin) are no-flux boundaries. The remaining two boundaries are periodic. There are two hundred initial rows.
(ZIP)

S3 Video Formation of isotropic triangular lattice on a cone frustum with a couple of initial columns (masked with white noise). The cone is mapped to a flat plane here. The inner boundary (closest to origin) and the outer boundary (farthest from origin) are no-flux boundaries. The remaining two boundaries are periodic. There are two hundred initial rows. Note that the dimensions of this simulation domain are different than that of S2 Video (because this lattice has isotropic UV spacing, not anisotropic UV spacing).
(ZIP)

S4 Video Formation of isotropic triangular lattice on a cone frustum with only a white noise mask (no initial column prepattern). The cone is mapped to a flat plane here. The inner boundary (closest to origin) and the outer boundary (farthest from origin) are no-flux boundaries. The remaining two boundaries are periodic. The dimensions of the simulation domain are the same as in S3 Video.
(ZIP)

S5 Video Formation of anisotropic triangular lattice on a cone frustum with only a white noise mask (no initial column prepattern). The cone is mapped to a flat plane here. The inner boundary (closest to origin) and the outer boundary (farthest from origin) are no-flux boundaries. The remaining two boundaries are periodic. The dimensions of the simulation domain are the same as in S2 Video.
(ZIP)

S6 Video Simulation corresponding to Fig 7D. This simulation is of an anisotropic triangular lattice on a cone frustum with a couple of initial columns (masked with white noise). The cone is mapped to a flat plane here. The inner boundary (closest to origin) and the outer boundary (farthest from origin) are no-flux boundaries. The remaining two boundaries are periodic. There are two hundred fifty initial rows and approximately one hundred fifty columns.
(ZIP)
