# Supplementary figures and images for: Defect patterns on the curved surface of fish retinae suggest a mechanism of cone mosaic formation
Source: PLoS Comput Biol. 2020 Dec 15;16(12):e1008437. doi: 10.1371/journal.pcbi.1008437 (PMC7771878; doi:10.1371/journal.pcbi.1008437)

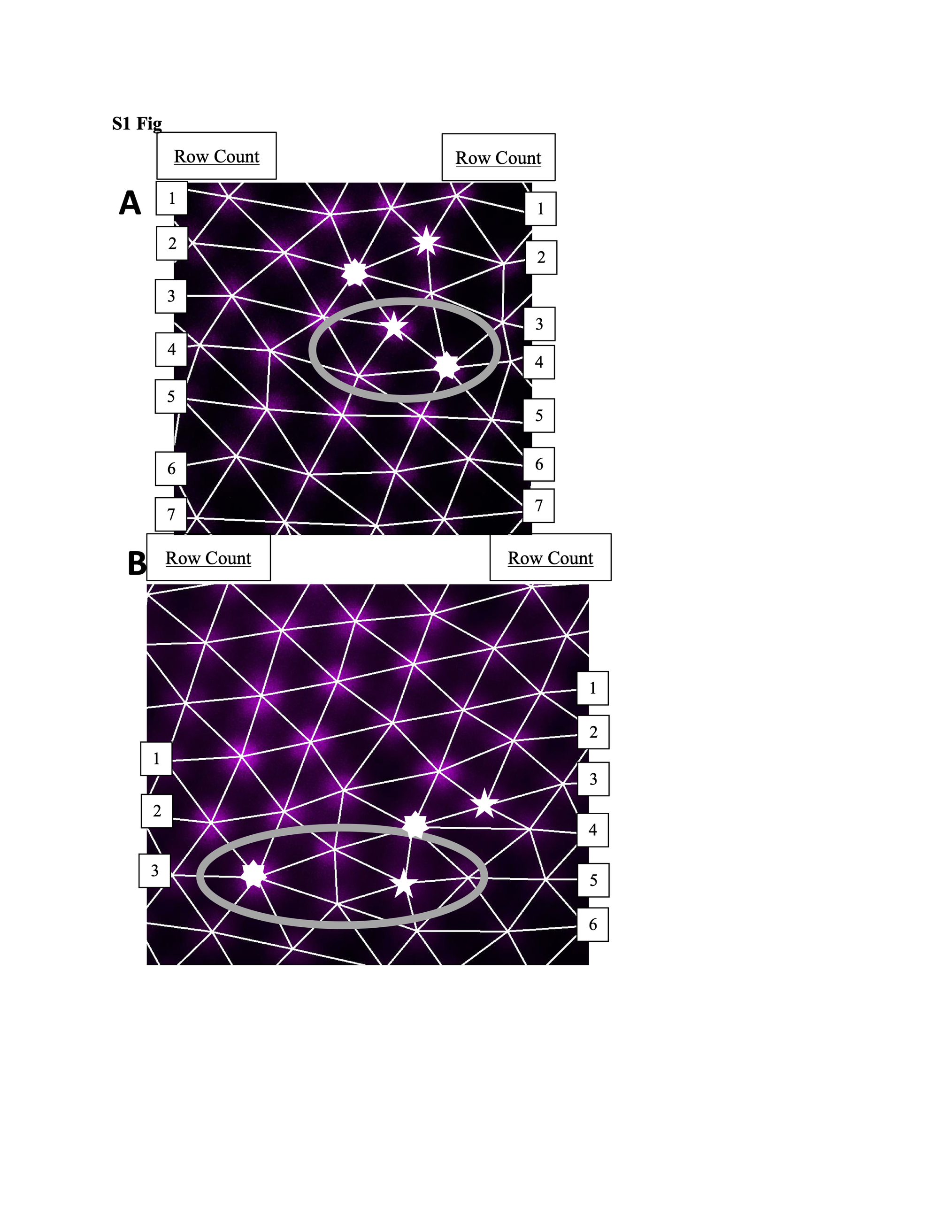

Supplement: S1 Fig — (A) In this image, the nuclear-localized, photoconverted protein in UV cones is pseudo-colored magenta. White bonds: triangulation connecting nearest neighbors. The seven- and five- coordinated UV cones: seven- and five-sided stars, respectively. A Y-Junction exists near the reverse Y-Junction. Gray oval encloses the reverse Y-Junction. Row counts are annotated on each side of the image. (B) Example of double-row insertion near a standard Y-Junction. The double-row insertion, enclosed by the gray oval, corresponds to a five- and seven-coordinated particle that are not directly connected by a bond in the lattice. Note that this double-row insertion does not disrupt the patterning of the cone mosaic. Row counts are annotated on each side of the image. (TIF) [file pcbi.1008437.s001.tif]

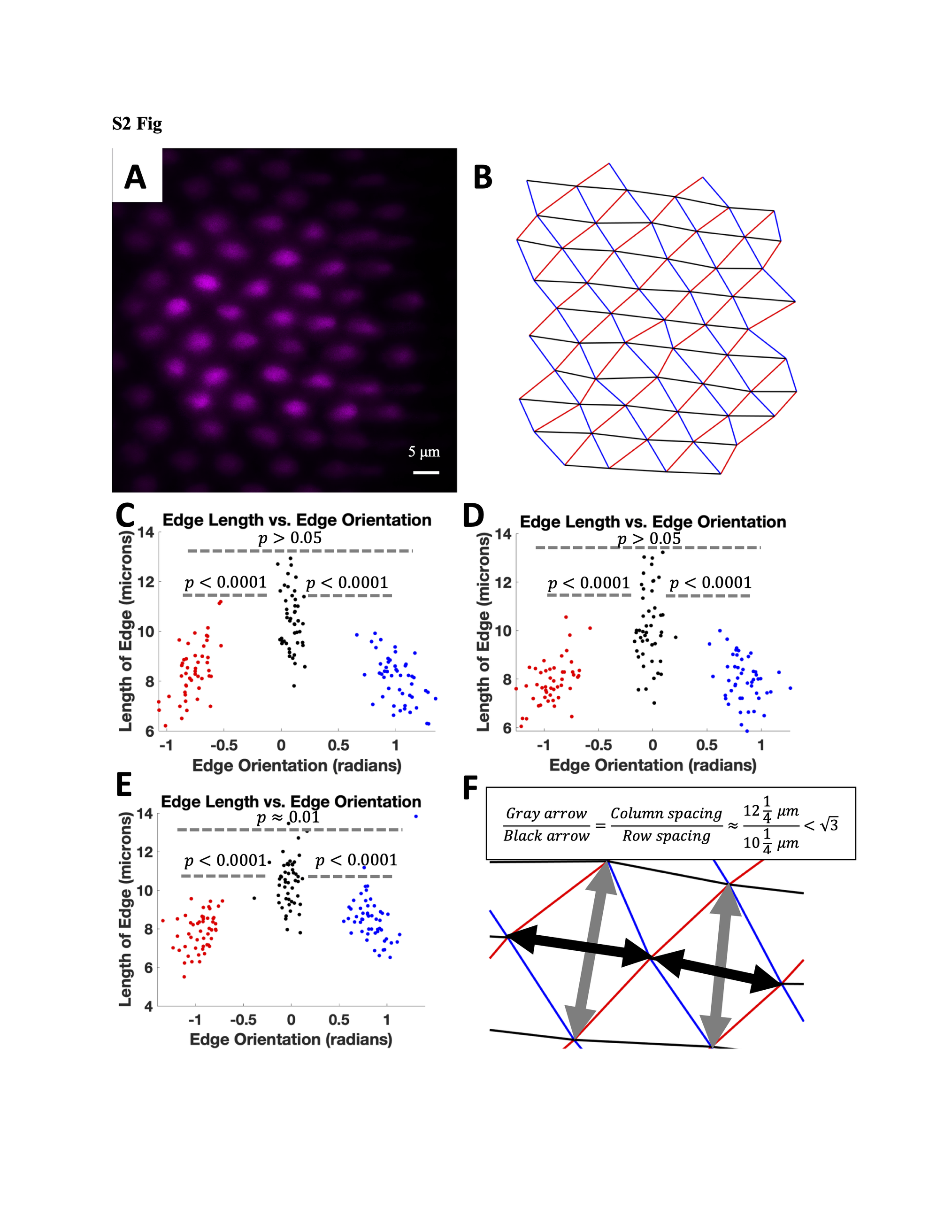

Supplement: S2 Fig — (A) Patch of photoconverted UV cones near the retinal margin. This patch of UV cones, that express nuclear-localized fluorescent protein, does not contain a Y-Junction. We use this patch, and two others, to quantify spacing between UV cones. (B) Triangulation for the patch of UV cones in panel A. In this triangulation, bonds connecting UV cones in the same row are black lines. Bonds along the other two principal directions of the lattice are blue and red (not at all related to Blue and Red cones). (C) Scatter plot of bond length versus bond orientation in triangulation from panel B. The same color scheme denotes bonds along the row direction and along the two other principal directions. p-values calculated via Mann-Whitney U-test. (D-E) Equivalent of panel C for two other samples. (F) The column direction is NOT a principal direction in the triangular lattice, meaning that UV cones in the same column are not each other’s nearest neighbors. Using a section of the triangulation from panel B, we illustrate spacing along the row direction by black arrows, and the spacing along the column direction by gray arrows. For an isotropic lattice, the column spacing is a square root of three times the row spacing. For this lattice, we can calculate the column spacing, given mean bond lengths in the three principal directions. We find that the column spacing is approximately twelve and a quarter microns, as compared to a row spacing of approximately ten and a quarter microns. This column to row spacing ratio is less than the square root of three, meaning that row bonds are elongated relative to the case of an isotropic lattice. (TIF) [file pcbi.1008437.s002.tif]

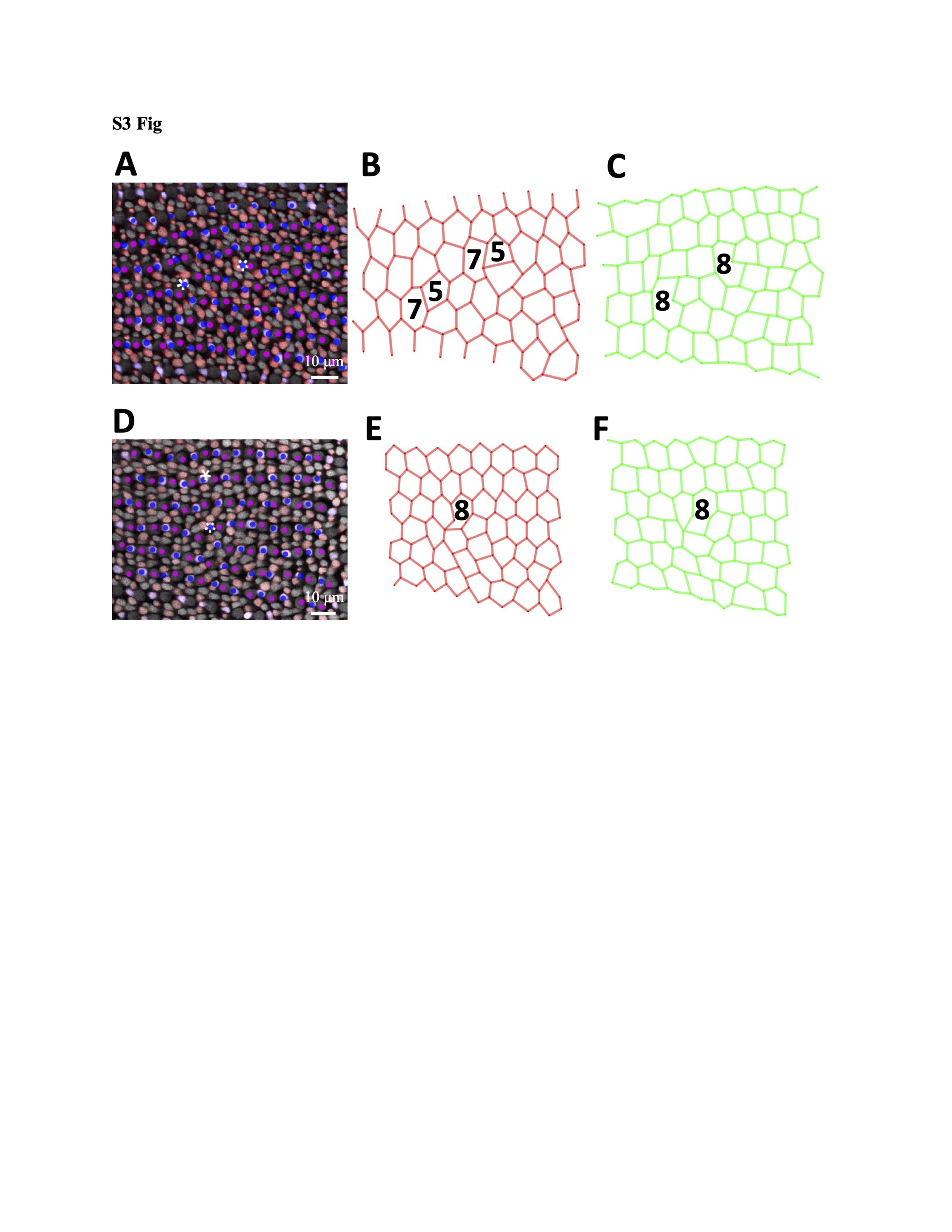

Supplement: S3 Fig — (A) Two Y-Junctions (asterisks) in a flat-mount retinal preparation from an adult, triple transgenic (Tg[sws2:GFP; trβ2:tdTomato; gnat2:CFP]) fish. Blue cones express a fluorescent reporter (pseudo-colored blue) under control of the Blue opsin promoter sws2, and Red cones express a fluorescent reporter (pseudo-colored red) under control of the trβ2 promoter. All cones express an additional fluorescent reporter under control of the gnat2 promoter. Although UV and Green cones do not express different fluorescent reporters, these two cone subtypes are morphologically distinguishable. (B) Nodes of graph are Red cones from panel A, and edges connect nearest neighbors in honeycomb lattice. Note the existence of a heptagon-pentagon pair (i.e., a ‘glide’ dislocation) in both defect cores. (C) Nodes of the graph are Green cones from panel A, and edges connect nearest neighbors in honeycomb lattice. Note the existence of an octagon (i.e., a ‘shuffle’ dislocation) in both defect cores. (D-F) Another example of a Y-Junction from a flat-mount retinal preparation from the same triple transgenic line (akin to panels A-C). (TIF) [file pcbi.1008437.s003.tif]

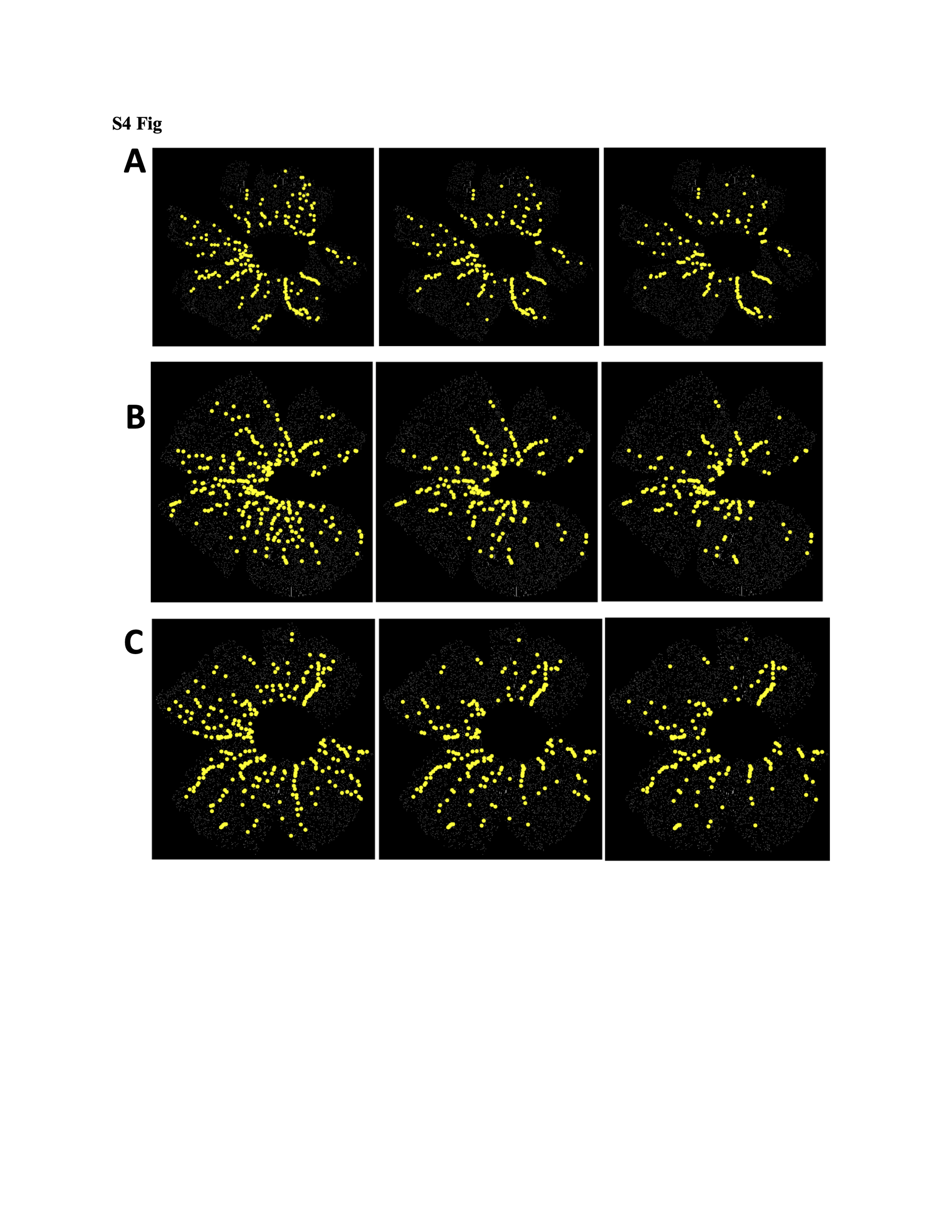

Supplement: S4 Fig — (A-C) For each of three flat-mounted retinae, image on the left-hand side is of all identified Y-junctions (yellow dots). Central image is all Y-junctions that our algorithm identified as in a grain boundary (with cutoff of twelve degrees). Image on the right-hand side is all Y-junctions that our algorithm identified as in a grain boundary (with cutoff of fourteen degrees). Panel A is fish 5 (total number of Y-Junctions = 249). Panel B is fish 4 (total number of Y-Junctions = 275). Panel C is fish 8 (total number of Y-Junctions = 285), the retina in Figs 3 and 6. See S2 Table. (TIF) [file pcbi.1008437.s004.tif]

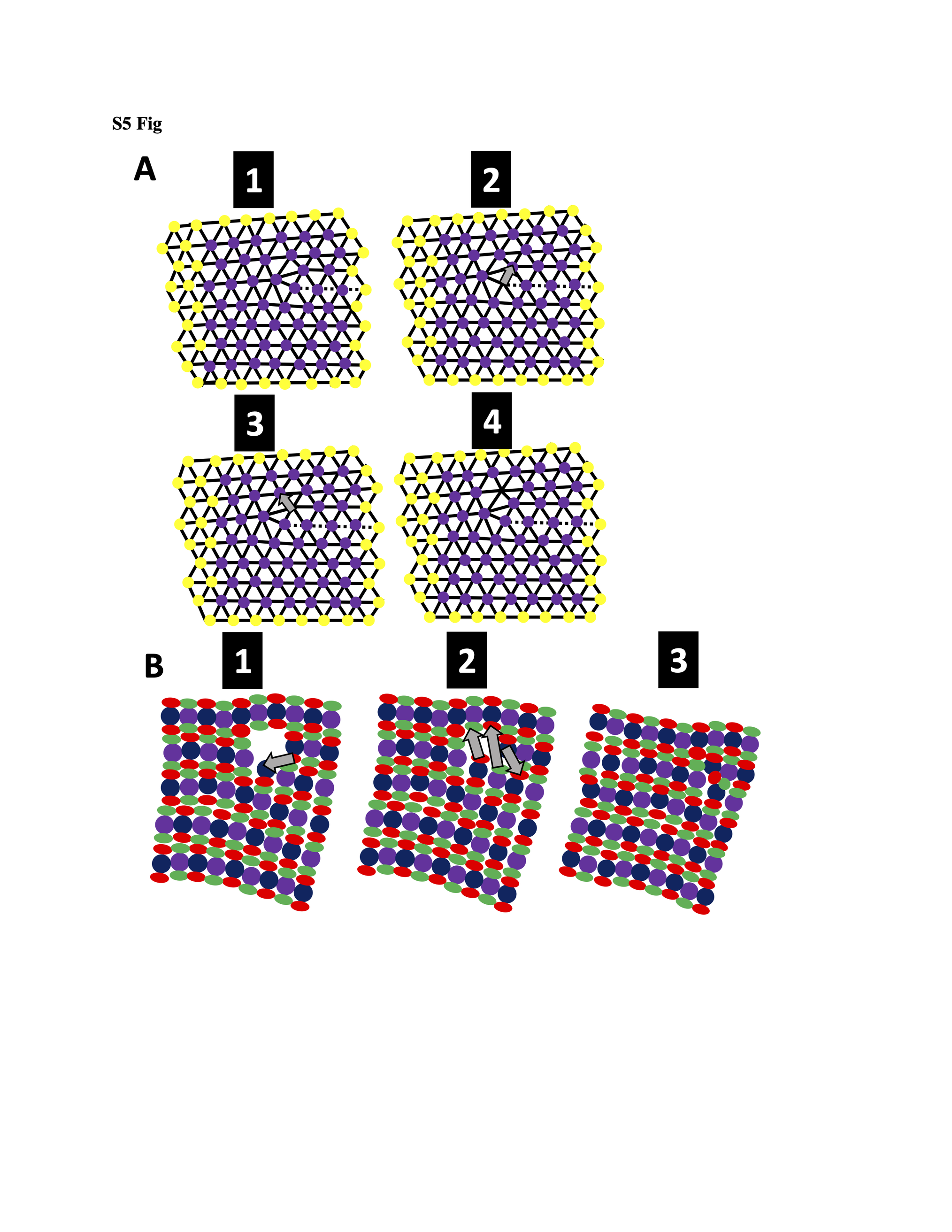

Supplement: S5 Fig — (A) The creation of a vacancy allows a dislocation to climb (i.e., move perpendicular to the Burgers vector). The lattice in panel 1 has a dislocation. Photoconverted UV cones are magenta, and non-photoconverted UV cones are yellow. Panel 2 is triangulation in panel 1 with a new vacancy. Gray arrow is where the vacancy will hop, to create the distribution in Panel 3. Gray arrow in Panel 3 is where the vacancy will hop, to create distribution in Panel 4. As the vacancy hops, the defect core moves perpendicular to the Burgers vector. (B) Vacancy in the cone mosaic (two missing Red cones, two missing Green cones, one missing Blue cone, and one missing UV cone) can be destroyed. Red cone in Panel 1 must move as indicated by gray arrow to create distribution in Panel 2. Movements denoted by gray arrows in Panel 2 allow for the vacancy to close and for the defect to move. Panel 3 corresponds to the distribution of cones after destruction of the vacancy. We never observe a vacancy (involving two missing Red cones, two missing Green cones, one missing Blue cone, and one missing UV cone) in the cone mosaic, and thus consider climb motion irrelevant for our system. (TIF) [file pcbi.1008437.s005.tif]

S7 Fig

**A**

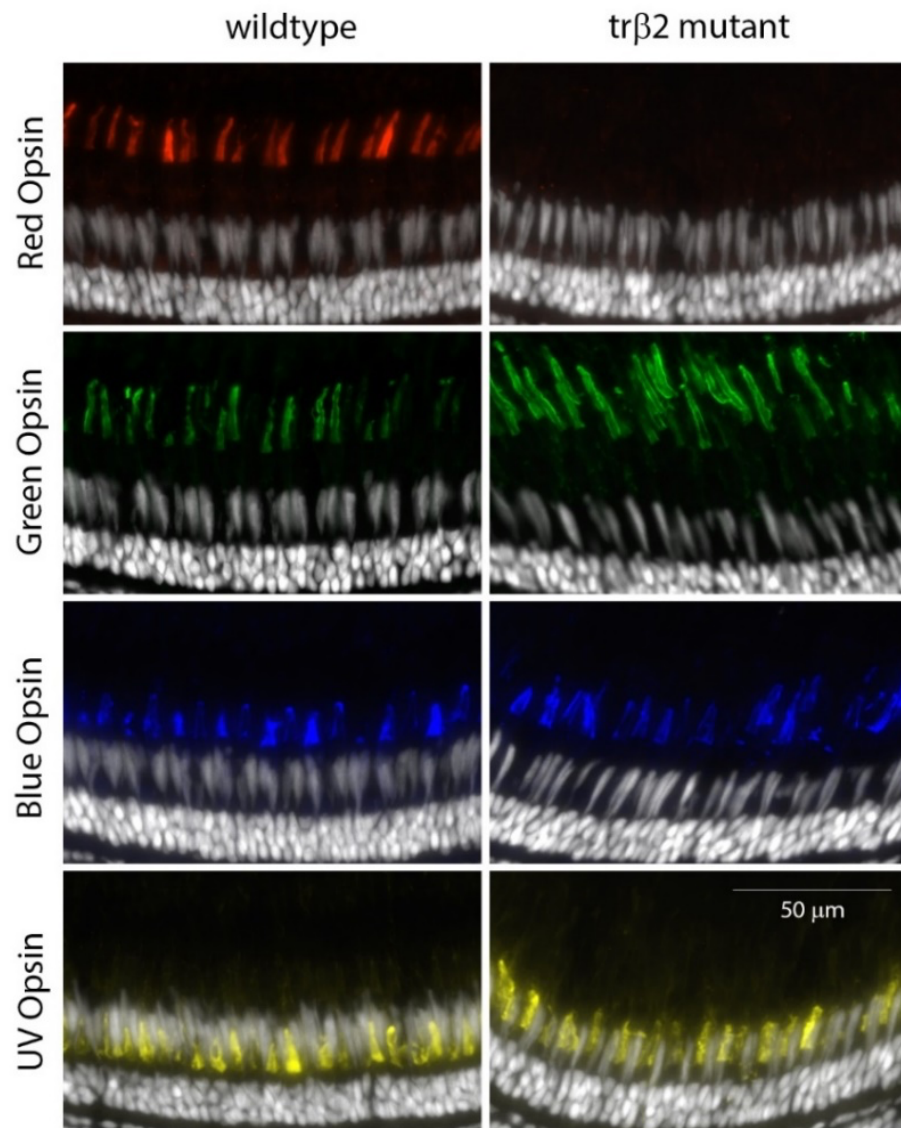

S7 Fig (Continued).

**B**

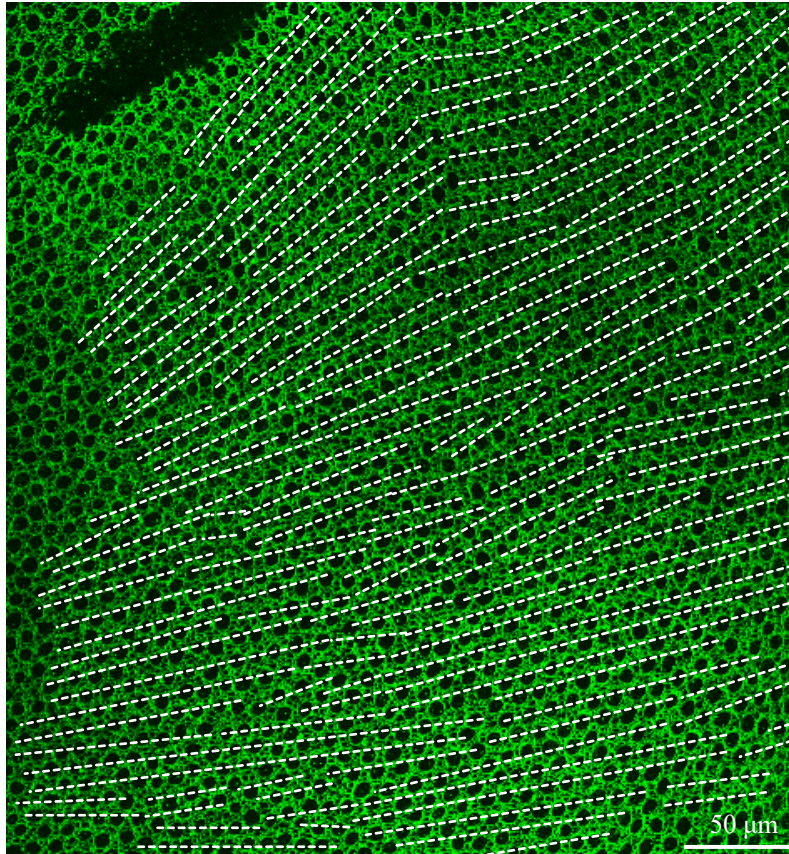

Supplement: S7 Fig — (A) Immunocytochemistry for cone-subtype-specific opsins, including Red opsin (red), Green opsin (green), Blue opsin (blue), and UV opsin (yellow) in wild-type and trβ2 mutant retinae. (B) Flat-mount retinal preparation of trβ2 mutant immunostained for ZO1 (green). Profiles of UV cone are large and rounded (see Fig 6C and 6D). White dashed lines: some rows of UV cones. (PDF) [file pcbi.1008437.s007.pdf]

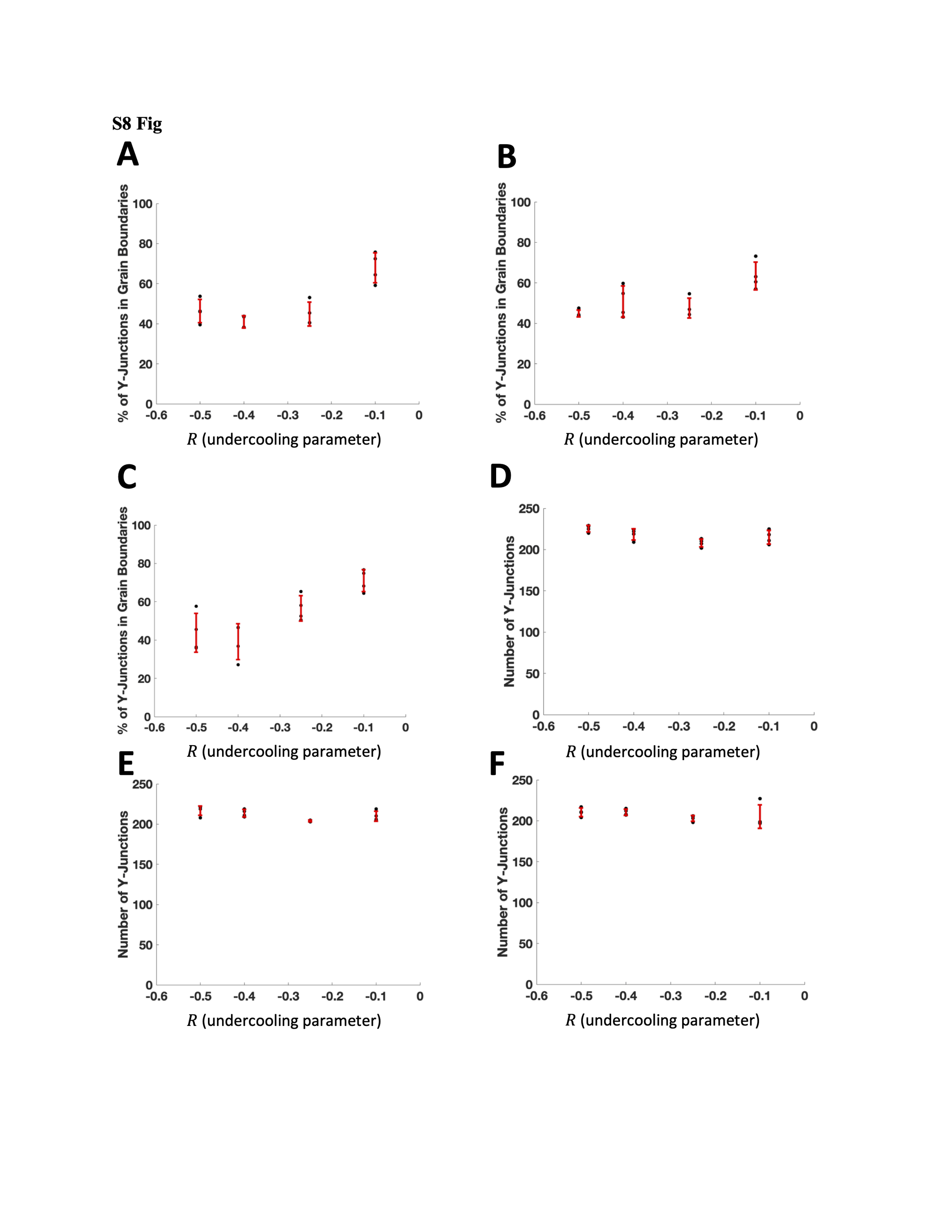

Supplement: S8 Fig — We take a one-dimensional cut of the two-dimensional phase diagram of the phase-field crystal model (ψ0=−−R2), where ψ0 is the mean of the density modulation field and where R is the undercooling parameter. The number of initial rows on the cone frustum is two hundred. Approximately ninety-five columns exist from the top of the cone frustum to the bottom. About two row insertions per added column are necessary to maintain constant cell-cell spacing. The degree of anisotropy is constrained by S2 Fig. We assume that the row orientation must rotate by twelve degrees or more at the site of a Y-Junction for that Y-Junction to be in a grain boundary (see S5 Table for the results of changing of this rotation parameter). (A) Standard deviation of white noise field, added to the first two columns, in these simulations is three-quarters. Along the one-dimensional cut of the phase diagram, we measure the fraction of Y-Junctions in grain boundaries. (B) The standard deviation of the white noise field in these simulations is one. Along the one-dimensional cut of the phase diagram, we measure the fraction of Y-Junctions in grain boundaries. (C) The standard deviation of the white noise field in these simulations is five-quarters. Along the one-dimensional cut of the phase diagram, we measure the fraction of Y-Junctions in grain boundaries. (D) For the same simulations in panel A, we plot the number of Y-Junctions. (E) For same simulations in panel B, we plot the number of Y-Junctions. (F) For same simulations in panel C, we plot the number of Y-Junctions. (TIF) [file pcbi.1008437.s008.tif]

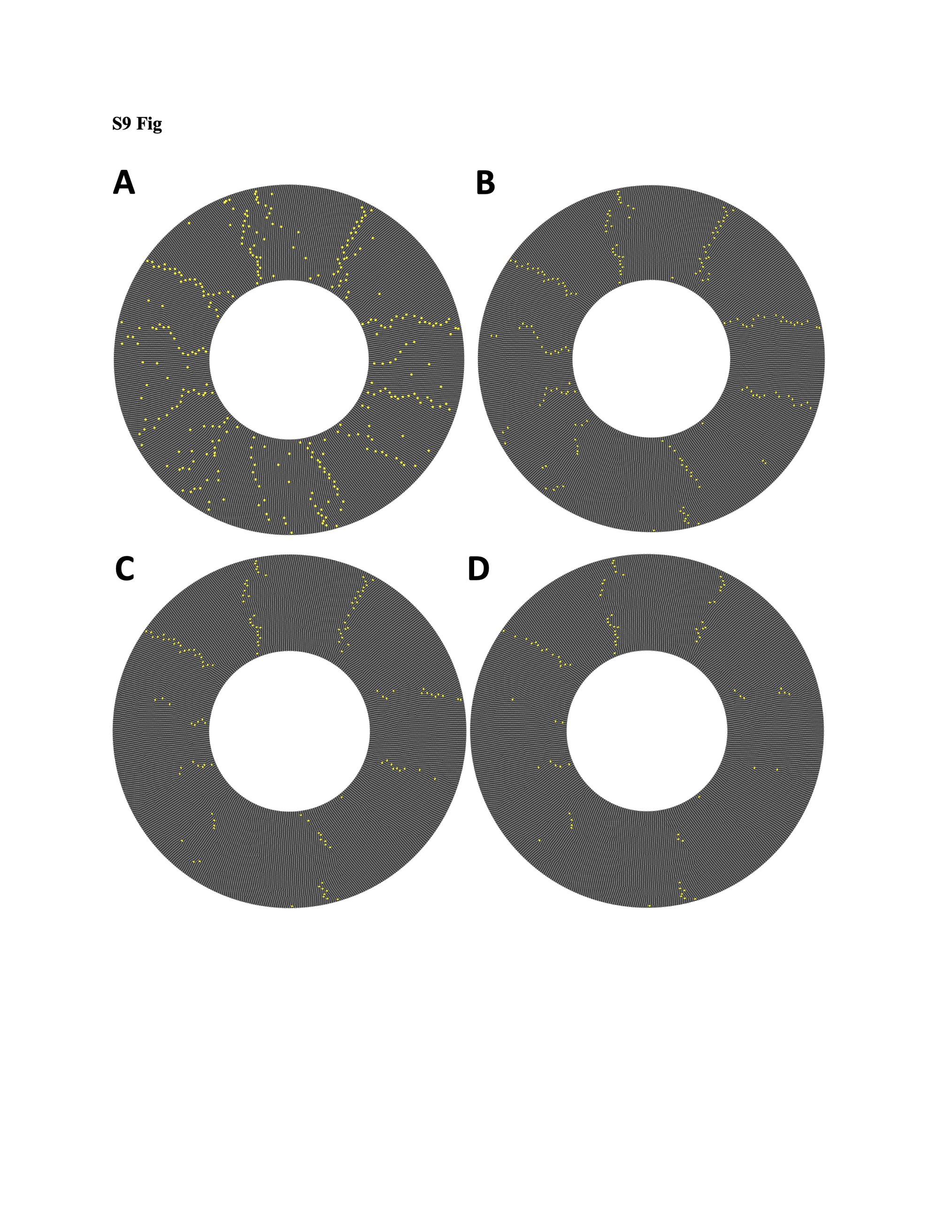

Supplement: S9 Fig — (A) Positions of all Y-Junctions (yellow dots). (B) Positions of all Y-Junctions (yellow dots) which coincide with a row orientation change of more than ten degrees. (C) Positions of all Y-Junctions (yellow dots) which coincide with a row orientation change of more than twelve degrees. (D) Positions of all Y-Junctions (yellow dots) which coincide with a row orientation change of more than fourteen degrees. See Detection of grain boundaries in simulated cone mosaic. (TIF) [file pcbi.1008437.s009.tif]

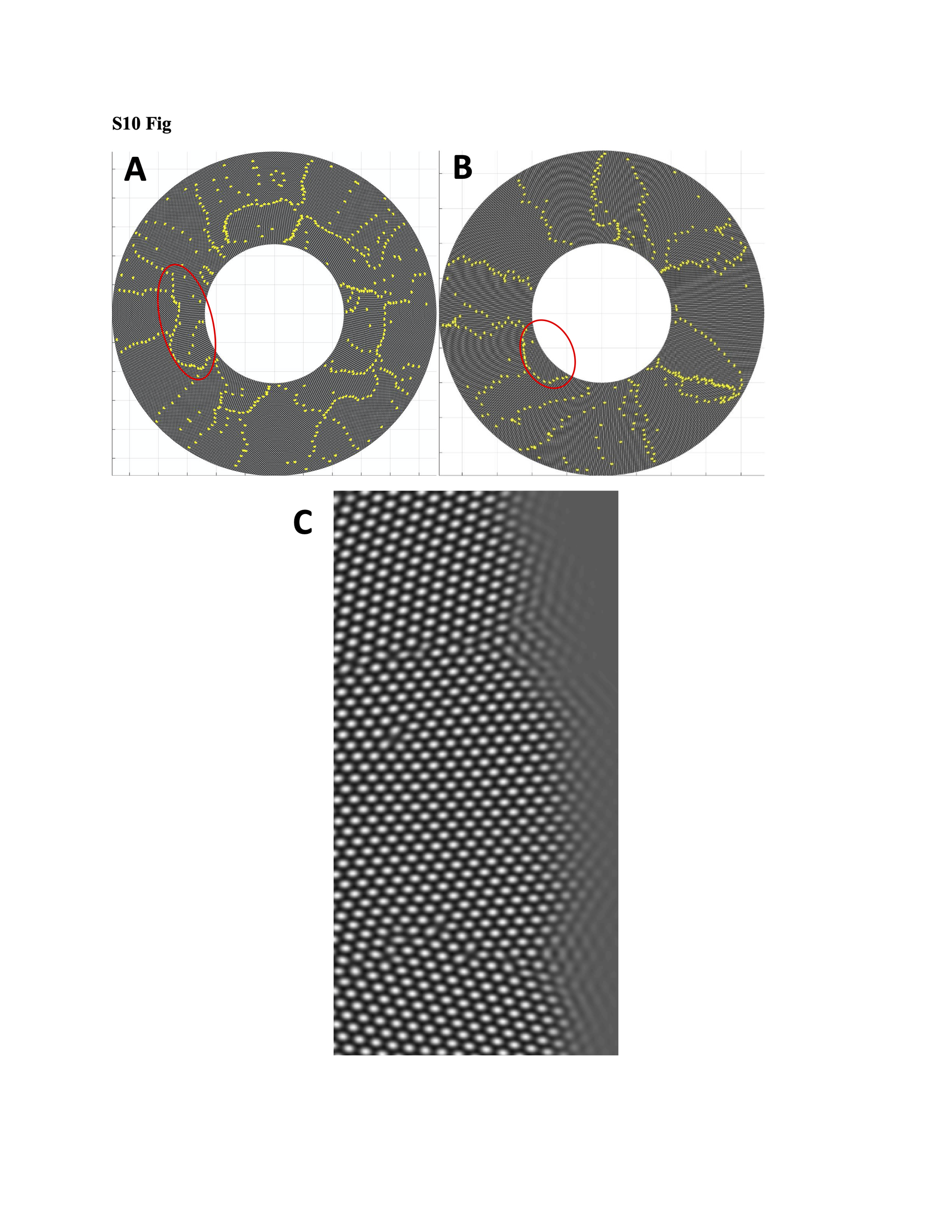

Supplement: S10 Fig — (A) Example of isotropic crystal growth on cone frustum with an initial column as prepattern. Yellow dots: seven-coordinated particles (including, but not limited to, those contained in Y-Junctions). Note the lines of seven-coordinated particles that do not radiate from the center of the cone to the periphery (example within red oval). These non-radiating lines of seven-coordinated particles result from a rotation of crystallographic orientation during growth of the isotropic crystal, not observed in zebrafish retinae. (B) Example of anisotropic crystal growth on cone frustum with no initial column as prepattern. With only white noise at the top of the cone in initial conditions, the anisotropy of the crystal (i.e., in the phase-field crystal free energy) selects and maintains the orientation during growth (in contrast with panel A). Even when a domain forms with improper orientation (example within red oval), the domain rotates to the proper orientation during growth. (C) Zoomed-in snapshot of an anisotropic phase-field crystal simulation on the surface of a cone. Note that near grain boundaries (i.e., where the domain rotation rotates), there is a lag in proper positioning of UV cones (i.e., density field remains poorly resolved) relative to growth of neighboring domains. This results in a characteristic V-shape. (TIF) [file pcbi.1008437.s010.tif]

S11 Fig

**A**

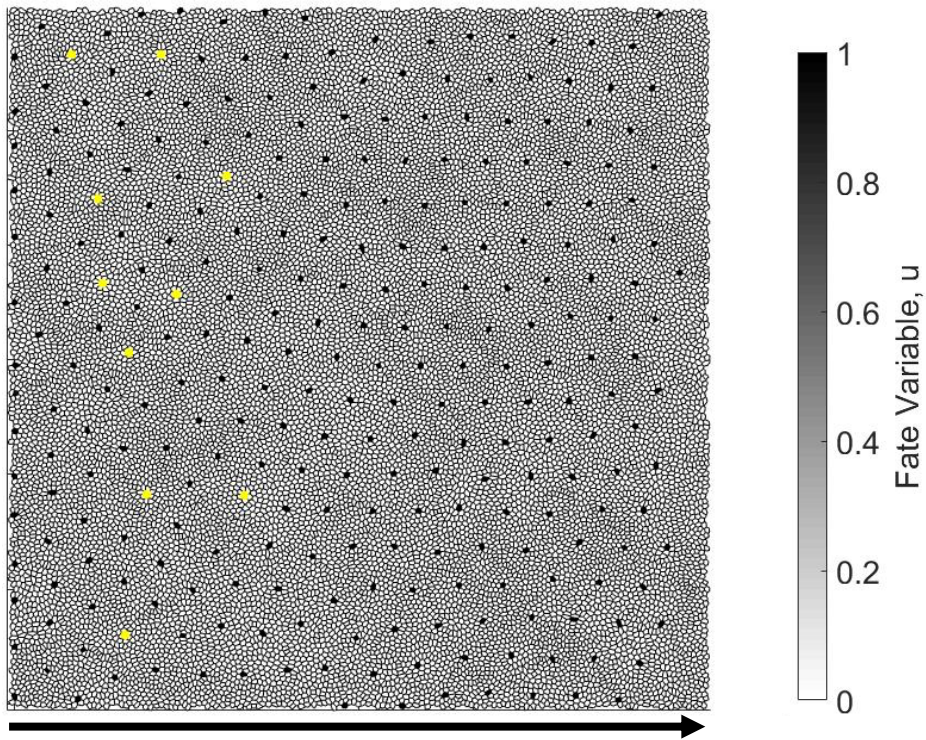

**B**

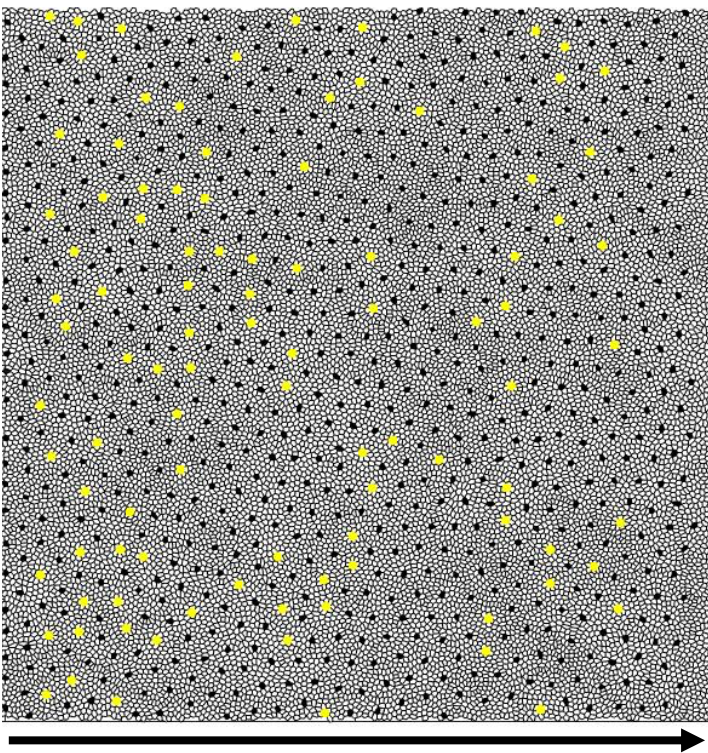

S11 Fig (Continued).

**C**

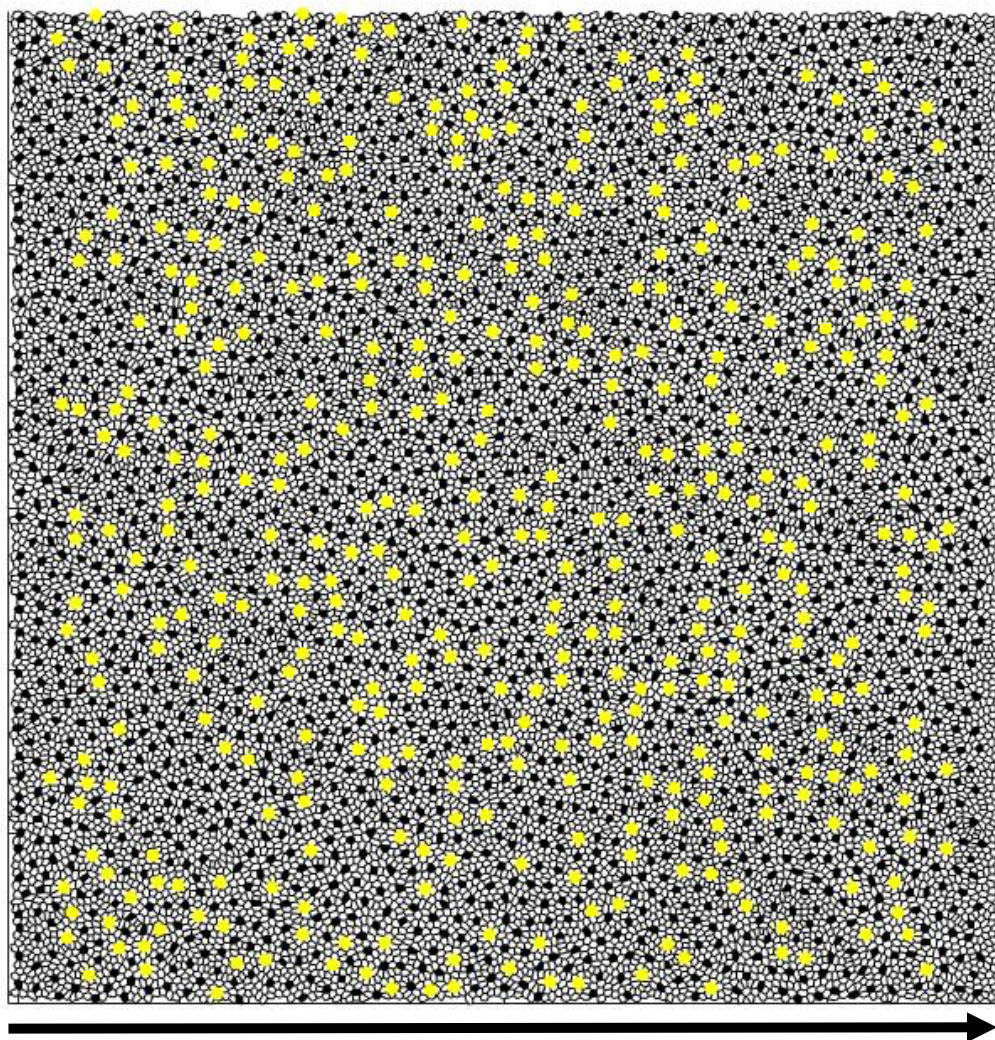

Supplement: S11 Fig — Triangular lattice of u≈1 cells forms on a square packing of 20000 cells with periodic boundary conditions. Defects (i.e., seven-coordinated) in triangular lattice of u≈1 cells are yellow dots. Initially, all cells are in state (u = 0) because an external inhibiting signal is provided to all cells. Starting at t = 0, a wave of de-inhibition moves from left to right (see Numerical solutions of lateral inhibition on disordered cell packing). The wave moves at a speed v=l4τ where τ is the timescale of cell differentiation, and l is the range of cell-cell signaling. In each panel, the black arrow is the direction of wave propagation. (A) The signaling range is 3A0, where A0 is the mean cell area. This signaling range results in seven to eight u≈0 cells between each pair of neighboring u≈1 cells in the final pattern. Note that some defects are generated early in pattern formation (i.e., left side of packing), but the right side of the packing contains no defects. (B) The signaling range is 1.75A0. This results in about five u≈0 cells between each pair of neighboring u≈1 cells in the final pattern. The entire packing contains defects. (C) The signaling range is 1A0, comparable to lattice spacing of the cone mosaic. This results in one to two u≈0 cells between each pair of neighboring u≈1 cells in the final pattern. The entire packing contains defects. This image is enlarged relative to panels A and B for the sake of clarity. (PDF) [file pcbi.1008437.s011.pdf]

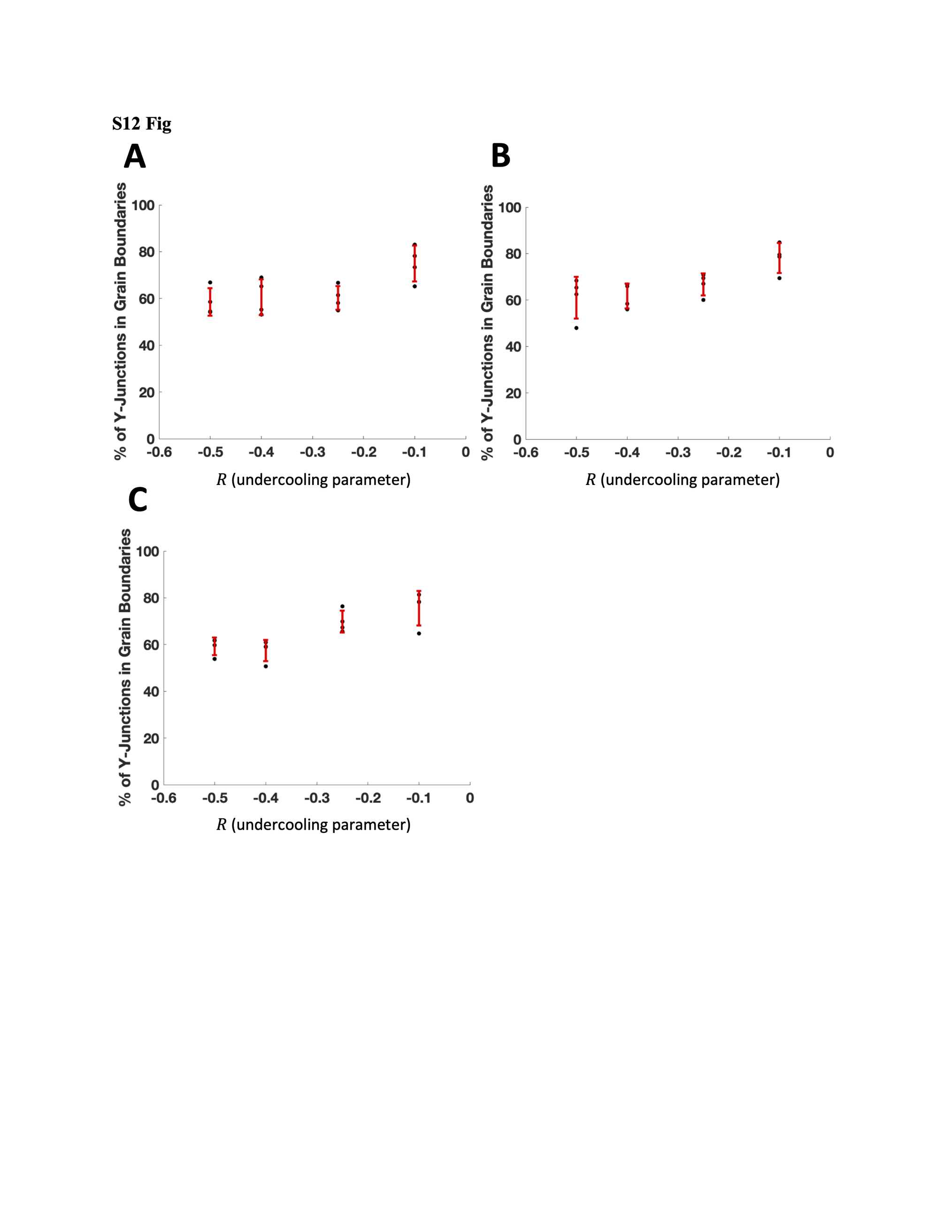

Supplement: S12 Fig — The simulations analyzed here are the same simulations as in S8 Fig. Instead of testing for the rotation of row orientation in the vicinity of a Y-Junction, here we search for Y-Junctions that are approximately linearly aligned with other Y-Junctions that are their nearest neighbors (see Alternative method for detection of grain boundaries). (A) Standard deviation of the white noise field, added to the first two columns, in these simulations is three-quarters. (B) Standard deviation of the white noise field in these simulations is one. (C) Standard deviation of the white noise field in these simulations is five-quarters. (TIF) [file pcbi.1008437.s012.tif]
