# Supplementary material for: Defect patterns on the curved surface of fish retinae suggest a mechanism of cone mosaic formation
Source: PLoS Comput Biol. 2020 Dec 15;16(12):e1008437. doi: 10.1371/journal.pcbi.1008437 (PMC7771878; doi:10.1371/journal.pcbi.1008437)

S6 Fig

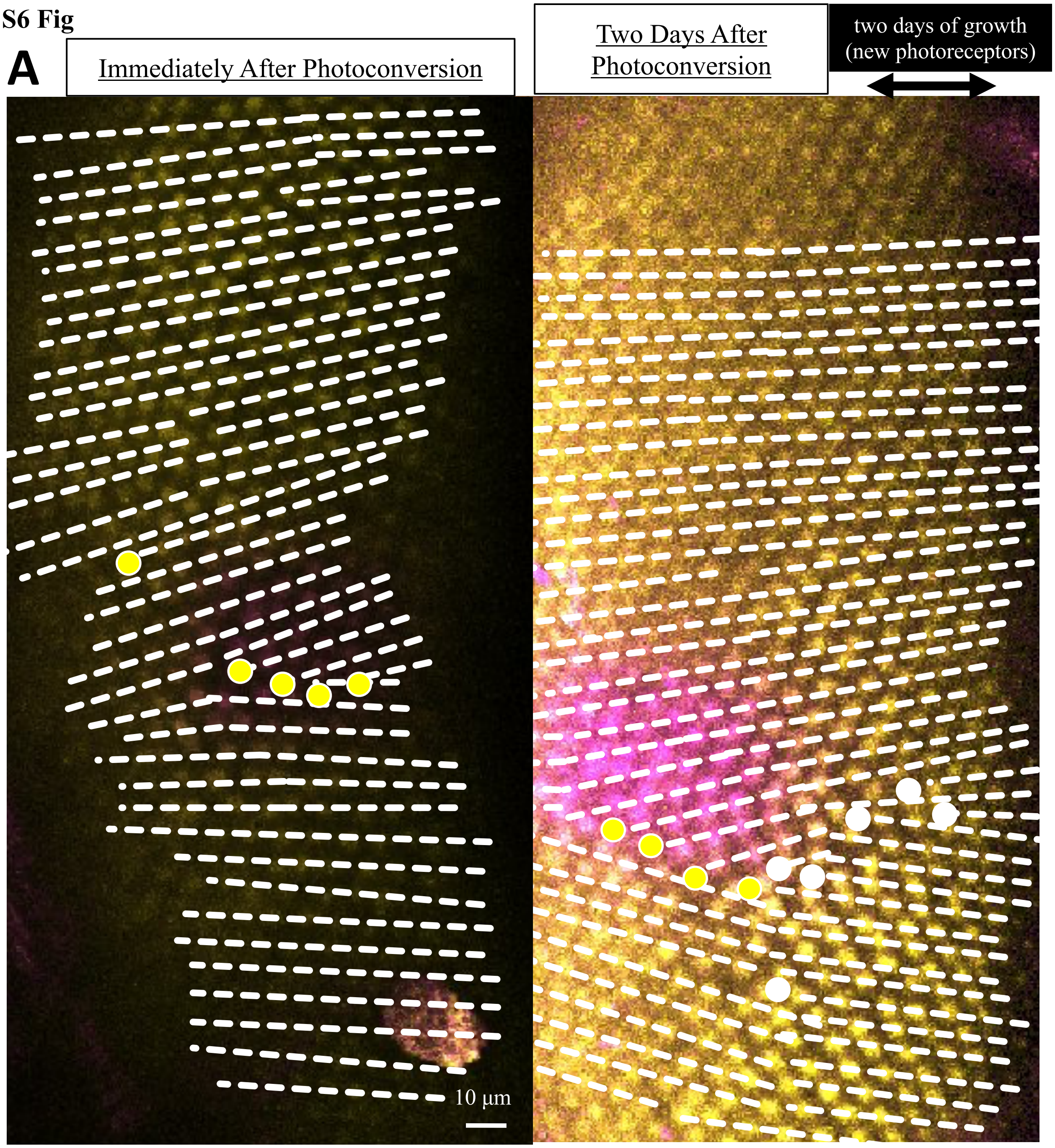
A
Immediately After Photoconversion
Two Days After Photoconversion
two days of growth (new photoreceptors)
10 µm

S6 Fig (Continued).

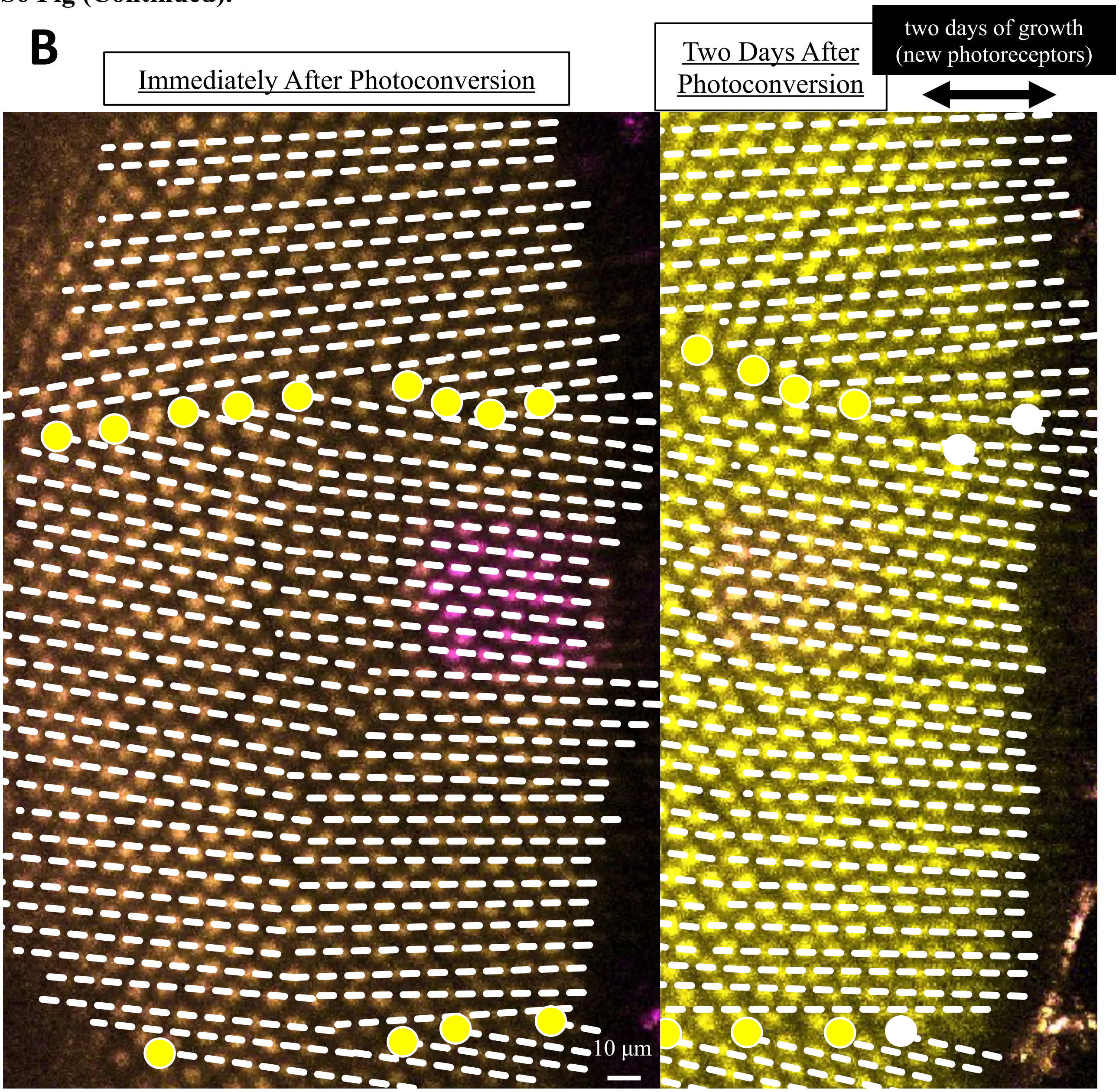

Supplement: S6 Fig — (A) We trace rows of UV cones (white dashed lines) near Y-Junctions. Yellow dots: Y-Junctions observed immediately after photoconversion. White dots: Y-Junctions that are incorporated during the two days after photoconversion. Double-sided black arrow: newly incorporated UV cone columns. This is grain boundary 1 in Table 2. (B) Grain boundaries 4–1 and 4–2 in Table 2. All row tracing and Y-Junctions denoted in same way as in panel A. (PDF) [file pcbi.1008437.s006.pdf]
