# Supplementary material for: Defect patterns on the curved surface of fish retinae suggest a mechanism of cone mosaic formation
Source: PLoS Comput Biol. 2020 Dec 15;16(12):e1008437. doi: 10.1371/journal.pcbi.1008437 (PMC7771878; doi:10.1371/journal.pcbi.1008437)
Supplement: S1 Table — The same fish numbers are used in Table 1. *There is a large-angle grain boundary in this retina, where patterning of the cone mosaic is slightly disrupted. There are potentially 10 additional reverse Y-Junctions associated with that large-angle grain boundary. (PDF) [file pcbi.1008437.s013.pdf]

Fish #	Number of (forward) Y-Junctions	Number of (reverse) Y-Junctions
1	155	0
2	166	5
3	221	0
4	275	5
5	249	14
6	184	2
7	182	10*
8	285	7
