# Supplementary material for: Defect patterns on the curved surface of fish retinae suggest a mechanism of cone mosaic formation
Source: PLoS Comput Biol. 2020 Dec 15;16(12):e1008437. doi: 10.1371/journal.pcbi.1008437 (PMC7771878; doi:10.1371/journal.pcbi.1008437)
Supplement: S2 Table — See Detection of grain boundaries in flat-mounted, row-traced retinae. Note that the percentage of Y-Junctions in grain boundaries depends strongly on the cutoff on the row orientation’s change; this threshold determines whether or not a Y-Junction is within a grain boundary. The percentage of Y-Junctions in grain boundaries depends only weakly on the size of the boxes over which we average row orientation (to calculate the row orientation’s change near the Y-Junction). (PDF) [file pcbi.1008437.s014.pdf]

| Domain Rotation Cutoff | Box Dimension | | | | | | | | |
|---|---|---|---|---|---|---|---|---|---|
| | 88 pixels=19.6 μm≈3 rows | | | 118 pixels=26.3 μm≈4 rows | | | 147 pixels=32.7 μm≈5 rows | | |
| 10.0° | Fish # | # Y-Junctions | # Y-Junctions in GB | Fish # | # Y-Junctions | # Y-Junctions in GB | Fish # | # Y-Junctions | # Y-Junctions in GB |
| | 1 | 155 | 144 | 1 | 155 | 141 | 1 | 155 | 140 |
| | 2 | 166 | 151 | 2 | 166 | 144 | 2 | 166 | 146 |
| | 3 | 221 | 209 | 3 | 221 | 204 | 3 | 221 | 199 |
| | 4 | 275 | 231 | 4 | 275 | 220 | 4 | 275 | 220 |
| | 5 | 249 | 220 | 5 | 249 | 212 | 5 | 249 | 205 |
| | 6 | 184 | 177 | 6 | 184 | 166 | 6 | 184 | 162 |
| | 7 | 182 | 167 | 7 | 182 | 156 | 7 | 182 | 156 |
| | 8 | 285 | 243 | 8 | 285 | 234 | 8 | 285 | 230 |
| | Average % in GB | | 90% | Average % in GB | | 86% | Average % in GB | | 85% |
| 12.0° | Fish # | # Y-Junctions | # Y-Junctions in GB | Fish # | # Y-Junctions | # Y-Junctions in GB | Fish # | # Y-Junctions | # Y-Junctions in GB |
| | 1 | 155 | 139 | 1 | 155 | 131 | 1 | 155 | 131 |
| | 2 | 166 | 138 | 2 | 166 | 137 | 2 | 166 | 137 |
| | 3 | 221 | 202 | 3 | 221 | 191 | 3 | 221 | 184 |
| | 4 | 275 | 197 | 4 | 275 | 191 | 4 | 275 | 189 |
| | 5 | 249 | 202 | 5 | 249 | 193 | 5 | 249 | 188 |
| | 6 | 184 | 169 | 6 | 184 | 157 | 6 | 184 | 146 |
| | 7 | 182 | 158 | 7 | 182 | 141 | 7 | 182 | 137 |
| | 8 | 285 | 221 | 8 | 285 | 209 | 8 | 285 | 204 |
| | Average % in GB | | 83% | Average % in GB | | 79% | Average % in GB | | 77% |
| 14.0° | Fish # | # Y-Junctions | # Y-Junctions in GB | Fish # | # Y-Junctions | # Y-Junctions in GB | Fish # | # Y-Junctions | # Y-Junctions in GB |
| | 1 | 155 | 128 | 1 | 155 | 119 | 1 | 155 | 120 |
| | 2 | 166 | 126 | 2 | 166 | 122 | 2 | 166 | 124 |
| | 3 | 221 | 192 | 3 | 221 | 175 | 3 | 221 | 176 |
| | 4 | 275 | 165 | 4 | 275 | 162 | 4 | 275 | 166 |
| | 5 | 249 | 180 | 5 | 249 | 163 | 5 | 249 | 165 |
| | 6 | 184 | 156 | 6 | 184 | 143 | 6 | 184 | 133 |
| | 7 | 182 | 149 | 7 | 182 | 132 | 7 | 182 | 125 |
| | 8 | 285 | 199 | 8 | 285 | 168 | 8 | 285 | 174 |
| | Average % in GB | | 75% | Average % in GB | | 69% | Average % in GB | | 69% |

Pixel size is 0.2227074 μm X 0.2227074 μm
