# Supplementary material for: Defect patterns on the curved surface of fish retinae suggest a mechanism of cone mosaic formation
Source: PLoS Comput Biol. 2020 Dec 15;16(12):e1008437. doi: 10.1371/journal.pcbi.1008437 (PMC7771878; doi:10.1371/journal.pcbi.1008437)
Supplement: S3 Table — Here we quantify the motion of Y-Junctions within photoconverted regions. The fish labels are the same as in Table 2. Note that some fish are missing from this list (e.g., 4 and 8). These two samples have grain boundaries in neighboring non-photoconverted regions, but do not have defects within the photoconverted region itself. For each sample, we fit the positions of UV cone nuclei in the photoconverted region to a plane in order to compute a triangulation. Because the photoconverted region is small relative to the radius of curvature of the retina, the positions of UV cone nuclei are well fit (as quantified by RMSE) by a plane at both imaging times. In the UV cone triangulations, we check for bond flips near the defect core between photoconversion and later imaging (see Tracking UV cone positions in photoconverted regions and measuring glide motion). If a Y-Junction glides by one row, we denote that with a 1, and if a Y-Junction does not glide, we denote that with a 0. (PDF) [file pcbi.1008437.s015.pdf]

Fish Label	# of Y-Junctions in Photoconverted Region	Days between Photoconversion and Later Imaging	RMSE for Fit Plane at Day 0 (μm)	RMSE for Fit Plane at Later Imaging (μm)	Glide Motion of Defect(s) In Units of Rows
1	2	2	0.85	0.63	(0, 0)
2	1	2	0.76	0.55	0
3	2	2	0.50	0.42	(0, 0)
5	1	2	0.70	0.26	1
6	1	2	1.0	0.59	0
7	1	2	0.48	0.63	1
9	1	2	0.96	0.53	0
10	2	3	1.1	0.62	(0, 1)
11	1	4	1.8	0.82	0
12	1	4	1.5	0.74	1
13	1	2	0.85	0.31	1
14	1	2	0.90	1.1	0
15	1	2	1.2	0.45	1
