# Supplementary material for: Defect patterns on the curved surface of fish retinae suggest a mechanism of cone mosaic formation
Source: PLoS Comput Biol. 2020 Dec 15;16(12):e1008437. doi: 10.1371/journal.pcbi.1008437 (PMC7771878; doi:10.1371/journal.pcbi.1008437)
Supplement: S4 Table — The fish labels are the same as in Table 2 and S6 Fig. We first list the number of Y-Junctions incorporated between photoconversion and later imaging (within the whole image, not just within the grain boundary). We list the average and median distances (i.e., along the margin axis) between a new Y-Junction and its nearest grain boundary if each new Y-Junction’s position is uncorrelated with positions of existing grain boundaries. We, then, list the actual distances (i.e., along the margin axis) between each newly incorporated Y-Junction and its nearest existing grain boundary in the image. Fish 8 is omitted because, within the image, no new Y-Junctions are added between photoconversion and later imaging. Note that whether a collection of defects is a grain boundary or not depends on how strongly the row orientation changes from one side of that collection of defects to the other side. We systematically change the cutoff in the angle of rotation of the row orientation that defines grain boundaries to see how our results depend on that cutoff. (PDF) [file pcbi.1008437.s016.pdf]

If we define a grain boundary (GB) as a region (at the retinal margin) at which the domain orientation rotates by 10 degrees or more at the time of photoconversion, the following table (and following p-value for significance of grain boundary growth) applies:

Fish Label	# of Y-Junctions Added Between Photoconversion and Later Imaging	Mean Distance Between New Y-Junction and Nearest GB if Uncorrelated (μm)	Median Distance Between New Y-Junction and Nearest GB if Uncorrelated (μm)	Actual Distance Between new Y-Junctions and GB (μm)
1	6	84	73	[9, 2, 15, 12, 12, 46]
2	2	91	73	[24, 4]
3	3	87	73	[1, 2, 65]
4	4	45	44	[24, 1, 5, 1]
5	1	73	73	1
6	2	75	73	[4, 1]
7	3	74	73	[11, 123, 42]
9	2	73	73	[18, 20]
10	2	94	78	[2, 2]
11	8	43	39	[12, 23, 45, 1, 0, 95, 20, 69]
12	4	39	36	[15, 5, 11, 36]

$$p < 0.000001$$

If we define a grain boundary (GB) as a region (at the retinal margin) at which the domain orientation rotates by 12 degrees or more at the time of photoconversion, the following table (and following p-value for significance of grain boundary growth) applies:

Fish Label	# of Y-Junctions Added Between Photoconversion and Later Imaging	Mean Distance Between New Y-Junction and Nearest GB if Uncorrelated (μm)	Median Distance Between New Y-Junction and Nearest GB if Uncorrelated (μm)	Actual Distance Between new Y-Junctions and GB (μm)
1	6	84	73	[9, 2, 15, 12, 12, 46]
2	2	91	73	[24, 4]
3	3	87	73	[1, 2, 65]
4	4	45	44	[24, 1, 5, 1]

6	2	75	73	[4, 1]
10	2	94	78	[2, 2]
11	8	99	88	[12, 23, 158, 205, 203, 108, 20, 69]
12	4	39	36	[15, 5, 11, 36]

$$p < 0.00006$$

If we define a grain boundary (GB) as a region (at the retinal margin) at which the domain orientation rotates by 14 degrees or more at the time of photoconversion, the following table (and following p-value for significance of grain boundary growth) applies:

Fish Label	# of Y-Junctions Added Between Photoconversion and Later Imaging	Mean Distance Between New Y-Junction and Nearest GB if Uncorrelated (μm)	Median Distance Between New Y-Junction and Nearest GB if Uncorrelated (μm)	Actual Distance Between new Y-Junctions and GB (μm)
1	6	84	73	[9, 2, 15, 12, 12, 46]
2	2	91	73	[24, 4]
3	3	87	73	[1, 2, 65]
4	4	45	44	[24, 1, 5, 1]
10	2	94	78	[2, 2]
11	8	99	88	[12, 23, 158, 205, 203, 108, 20, 69]
12	4	39	36	[15, 5, 11, 36]

$$p = 0.0003$$
