# Supplementary material for: Defect patterns on the curved surface of fish retinae suggest a mechanism of cone mosaic formation
Source: PLoS Comput Biol. 2020 Dec 15;16(12):e1008437. doi: 10.1371/journal.pcbi.1008437 (PMC7771878; doi:10.1371/journal.pcbi.1008437)
Supplement: S5 Table — We group the simulations into distinct tables based on the value of the undercooling parameter R. Within each table, the noise in the initial conditions is varied. Note that each simulation has a unique ID, which we list in the same column as the simulation parameters. For each simulation, we list the total number of Y-Junctions and the number of Y-Junctions in grain boundaries. We find that the results depend weakly on the size of the boxes over which we average row orientation (see Detection of grain boundaries in simulated cone mosaic) but depend strongly on the cutoff in row orientation used to define whether a Y-Junction is in a grain boundary. (PDF) [file pcbi.1008437.s017.pdf]

For all simulations in the below table, $R = -\frac{1}{4}; \psi_0 = -\frac{\sqrt{-R}}{2}$

Domain Rotation Cutoff		Box Dimension (in units of row width)								
		≈3			≈4			≈5		
	10.0°	noise = $\frac{3}{4}$	# Y-Junctions	# Y-Junctions in GB	noise = $\frac{3}{4}$	# Y-Junctions	# Y-Junctions in GB	noise = $\frac{3}{4}$	# Y-Junctions	# Y-Junctions in GB
		68817	213	146	68817	213	137	68817	213	133
		153033	202	121	153033	202	118	153033	202	109
		4526243	210	122	4526243	210	115	4526243	210	115
		24927051	207	130	24927051	207	120	24927051	207	112
		Average % in GB		62%	Average % in GB		59%	Average % in GB		56%
		noise = 1	# Y-Junctions	# Y-Junctions in GB	noise = 1	# Y-Junctions	# Y-Junctions in GB	noise = 1	# Y-Junctions	# Y-Junctions in GB
		56824	205	119	56824	205	121	56824	205	120
		452849	205	130	452849	205	122	452849	205	116
		7967093	203	145	7967093	203	136	7967093	203	137
		48068598	203	127	48068598	203	121	48068598	203	115
		Average % in GB		64%	Average % in GB		61%	Average % in GB		60%
		noise = $\frac{5}{4}$	# Y-Junctions	# Y-Junctions in GB	noise = $\frac{5}{4}$	# Y-Junctions	# Y-Junctions in GB	noise = $\frac{5}{4}$	# Y-Junctions	# Y-Junctions in GB
		990	203	137	990	203	139	990	203	134
		14026	206	145	14026	206	141	14026	206	131
		100657	198	129	100657	198	128	100657	198	125
		271575	205	156	271575	205	157	271575	205	162
		Average % in GB		70%	Average % in GB		70%	Average % in GB		68%
	12.0°	noise = $\frac{3}{4}$	# Y-Junctions	# Y-Junctions in GB	noise = $\frac{3}{4}$	# Y-Junctions	# Y-Junctions in GB	noise = $\frac{3}{4}$	# Y-Junctions	# Y-Junctions in GB
		68817	213	114	68817	213	113	68817	213	112
		153033	202	76	153033	202	82	153033	202	86
		4526243	210	88	4526243	210	85	4526243	210	89
		24927051	207	87	24927051	207	94	24927051	207	86
		Average % in GB		44%	Average % in GB		45%	Average % in GB		45%
		noise = 1	# Y-Junctions	# Y-Junctio	noise = 1	# Y-Junctions	# Y-Junctio	noise = 1	# Y-Junctions	# Y-Junctio

				ns in GB			ns in GB			ns in GB
		56824	205	89	56824	205	91	56824	205	86
		452849	205	95	452849	205	96	452849	205	92
		7967093	203	109	7967093	203	111	7967093	203	111
		48068598	203	95	48068598	203	90	48068598	203	95
		Average % in GB		48%	Average % in GB		48%	Average % in GB		47%
		noise = $\frac{5}{4}$	# Y-Junctions	# Y-Junctions in GB	noise = $\frac{5}{4}$	# Y-Junctions	# Y-Junctions in GB	noise = $\frac{5}{4}$	# Y-Junctions	# Y-Junctions in GB
		990	203	112	990	203	118	990	203	119
		14026	206	106	14026	206	104	14026	206	98
		100657	198	96	100657	198	104	100657	198	104
		271575	205	129	271575	205	134	271575	205	131
		Average % in GB		55%	Average % in GB		57%	Average % in GB		56%
		noise = $\frac{3}{4}$	# Y-Junctions	# Y-Junctions in GB	noise = $\frac{3}{4}$	# Y-Junctions	# Y-Junctions in GB	noise = $\frac{3}{4}$	# Y-Junctions	# Y-Junctions in GB
		68817	213	81	68817	213	89	68817	213	88
		153033	202	51	153033	202	58	153033	202	66
		4526243	210	68	4526243	210	68	4526243	210	62
		24927051	207	65	24927051	207	71	24927051	207	67
		Average % in GB		32%	Average % in GB		34%	Average % in GB		34%
		noise = 1	# Y-Junctions	# Y-Junctions in GB	noise = 1	# Y-Junctions	# Y-Junctions in GB	noise = 1	# Y-Junctions	# Y-Junctions in GB
	14.0°	56824	205	65	56824	205	66	56824	205	57
		452849	205	68	452849	205	70	452849	205	65
		7967093	203	83	7967093	203	87	7967093	203	82
		48068598	203	67	48068598	203	67	48068598	203	69
		Average % in GB		35%	Average % in GB		36%	Average % in GB		33%
		noise = $\frac{5}{4}$	# Y-Junctions	# Y-Junctions in GB	noise = $\frac{5}{4}$	# Y-Junctions	# Y-Junctions in GB	noise = $\frac{5}{4}$	# Y-Junctions	# Y-Junctions in GB
		990	203	88	990	203	90	990	203	93
		14026	206	74	14026	206	77	14026	206	65
		100657	198	80	100657	198	82	100657	198	81
		271575	205	91	271575	205	94	271575	205	90
		Average % in GB		41%	Average % in GB		42%	Average % in GB		41%

For all simulations in the below table, $R = -\frac{1}{2}; \psi_0 = -\frac{\sqrt{-R}}{2}$

Domain Rotation Cutoff		Box Dimension (in units of row width)								
		≈3			≈4			≈5		
	10.0°	noise = $\frac{3}{4}$	# Y-Junctions	# Y-Junctions in GB	noise = $\frac{3}{4}$	# Y-Junctions	# Y-Junctions in GB	noise = $\frac{3}{4}$	# Y-Junctions	# Y-Junctions in GB
		582	225	136	582	225	136	582	225	126
		70355	220	140	70355	220	146	70355	220	141
		136537	229	147	136537	229	139	136537	229	136
		9052576	228	150	9052576	228	143	9052576	228	130
		Average % in GB		64%	Average % in GB		63%	Average % in GB		59%
		noise = 1	# Y-Junctions	# Y-Junctions in GB	noise = 1	# Y-Junctions	# Y-Junctions in GB	noise = 1	# Y-Junctions	# Y-Junctions in GB
		35260	221	138	35260	221	126	35260	221	129
		161783	208	123	161783	208	116	161783	208	109
		594379	219	133	594379	219	124	594379	219	130
		4864909	219	143	4864909	219	131	4864909	219	121
		Average % in GB		62%	Average % in GB		57%	Average % in GB		56%
		noise = $\frac{5}{4}$	# Y-Junctions	# Y-Junctions in GB	noise = $\frac{5}{4}$	# Y-Junctions	# Y-Junctions in GB	noise = $\frac{5}{4}$	# Y-Junctions	# Y-Junctions in GB
		1079	210	141	1079	210	147	1079	210	144
		110372	217	116	110372	217	105	110372	217	100
		293012	204	122	293012	204	110	293012	204	108
		600365	211	126	600365	211	126	600365	211	125
		Average % in GB		60%	Average % in GB		58%	Average % in GB		57%
	12.0°	noise = $\frac{3}{4}$	# Y-Junctions	# Y-Junctions in GB	noise = $\frac{3}{4}$	# Y-Junctions	# Y-Junctions in GB	noise = $\frac{3}{4}$	# Y-Junctions	# Y-Junctions in GB
		582	225	100	582	225	89	582	225	98
		70355	220	121	70355	220	118	70355	220	110
		136537	229	103	136537	229	106	136537	229	99
		9052576	228	105	9052576	228	105	9052576	228	97
		Average % in GB		48%	Average % in GB		46%	Average % in GB		45%
		noise = 1	# Y-Junctions	# Y-Junctions in GB	noise = 1	# Y-Junctions	# Y-Junctions in GB	noise = 1	# Y-Junctions	# Y-Junctions in GB

		35260	221	109	35260	221	105	35260	221	108
		161783	208	93	161783	208	92	161783	208	87
		594379	219	97	594379	219	96	594379	219	86
		4864909	219	98	4864909	219	96	4864909	219	90
		Average % in GB		46%	Average % in GB		45%	Average % in GB		43%
		noise = $\frac{5}{4}$	# Y-Junctions	# Y-Junctions in GB	noise = $\frac{5}{4}$	# Y-Junctions	# Y-Junctions in GB	noise = $\frac{5}{4}$	# Y-Junctions	# Y-Junctions in GB
		1079	210	117	1079	210	121	1079	210	117
		110372	217	74	110372	217	78	110372	217	73
		293012	204	82	293012	204	74	293012	204	74
		600365	211	88	600365	211	96	600365	211	86
		Average % in GB		43%	Average % in GB		44%	Average % in GB		42%
		noise = $\frac{3}{4}$	# Y-Junctions	# Y-Junctions in GB	noise = $\frac{3}{4}$	# Y-Junctions	# Y-Junctions in GB	noise = $\frac{3}{4}$	# Y-Junctions	# Y-Junctions in GB
		582	225	71	582	225	68	582	225	67
		70355	220	98	70355	220	97	70355	220	90
		136537	229	81	136537	229	78	136537	229	73
		9052576	228	77	9052576	228	74	9052576	228	71
		Average % in GB		36%	Average % in GB		35%	Average % in GB		33%
		noise = 1	# Y-Junctions	# Y-Junctions in GB	noise = 1	# Y-Junctions	# Y-Junctions in GB	noise = 1	# Y-Junctions	# Y-Junctions in GB
	14.0°	35260	221	87	35260	221	87	35260	221	88
		161783	208	69	161783	208	66	161783	208	68
		594379	219	67	594379	219	59	594379	219	56
		4864909	219	79	4864909	219	71	4864909	219	64
		Average % in GB		35%	Average % in GB		33%	Average % in GB		32%
		noise = $\frac{5}{4}$	# Y-Junctions	# Y-Junctions in GB	noise = $\frac{5}{4}$	# Y-Junctions	# Y-Junctions in GB	noise = $\frac{5}{4}$	# Y-Junctions	# Y-Junctions in GB
		1079	210	98	1079	210	97	1079	210	92
		110372	217	43	110372	217	44	110372	217	38
		293012	204	53	293012	204	56	293012	204	54
		600365	211	68	600365	211	66	600365	211	61
		Average % in GB		31%	Average % in GB		31%	Average % in GB		29%

For all simulations in the below table, $R = -\frac{2}{5}; \psi_0 = -\frac{\sqrt{-R}}{2}$

		Box Dimension (in units of row width)								
		≈3			≈4			≈5		
Domain Rotation Cutoff	10.0°	noise = $\frac{3}{4}$	# Y-Junctions	# Y-Junctions in GB	noise = $\frac{3}{4}$	# Y-Junctions	# Y-Junctions in GB	noise = $\frac{3}{4}$	# Y-Junctions	# Y-Junctions in GB
		906	209	127	906	209	118	906	209	110
		4047	224	138	4047	224	124	4047	224	127
		90525	219	131	90525	219	118	90525	219	121
		882232	222	118	882232	222	116	882232	222	111
		Average % in GB		59%	Average % in GB		55%	Average % in GB		54%
		noise = 1	# Y-Junctions	# Y-Junctions in GB	noise = 1	# Y-Junctions	# Y-Junctions in GB	noise = 1	# Y-Junctions	# Y-Junctions in GB
		5062	209	138	5062	209	127	5062	209	122
		29490	216	142	29490	216	126	29490	216	121
		65719	219	157	65719	219	139	65719	219	142
		1275730	211	149	1275730	211	149	1275730	211	141
		Average % in GB		69%	Average % in GB		63%	Average % in GB		62%
		noise = $\frac{5}{4}$	# Y-Junctions	# Y-Junctions in GB	noise = $\frac{5}{4}$	# Y-Junctions	# Y-Junctions in GB	noise = $\frac{5}{4}$	# Y-Junctions	# Y-Junctions in GB
		55142	208	130	55142	208	124	55142	208	123
		151274	215	135	151274	215	139	151274	215	125
		569271	212	121	569271	212	125	569271	212	110
		1006568	207	116	1006568	207	107	1006568	207	98
		Average % in GB		60%	Average % in GB		59%	Average % in GB		54%
	12.0°	noise = $\frac{3}{4}$	# Y-Junctions	# Y-Junctions in GB	noise = $\frac{3}{4}$	# Y-Junctions	# Y-Junctions in GB	noise = $\frac{3}{4}$	# Y-Junctions	# Y-Junctions in GB
		906	209	82	906	209	80	906	209	81
		4047	224	102	4047	224	98	4047	224	91
		90525	219	96	90525	219	95	90525	219	96
		882232	222	78	882232	222	85	882232	222	83
		Average % in GB		41%	Average % in GB		41%	Average % in GB		40%
		noise = 1	# Y-Junctions	# Y-Junctions in GB	noise = 1	# Y-Junctions	# Y-Junctions in GB	noise = 1	# Y-Junctions	# Y-Junctions in GB

		5062	209	90	5062	209	95	5062	209	97
		29490	216	97	29490	216	93	29490	216	90
		65719	219	114	65719	219	120	65719	219	114
		1275730	211	121	1275730	211	126	1275730	211	116
		Average % in GB		49%	Average % in GB		51%	Average % in GB		49%
		noise = $\frac{5}{4}$	# Y-Junctions	# Y-Junctions in GB	noise = $\frac{5}{4}$	# Y-Junctions	# Y-Junctions in GB	noise = $\frac{5}{4}$	# Y-Junctions	# Y-Junctions in GB
		55142	208	97	55142	208	97	55142	208	90
		151274	215	94	151274	215	100	151274	215	92
		569271	212	86	569271	212	78	569271	212	74
		1006568	207	57	1006568	207	56	1006568	207	59
		Average % in GB		40%	Average % in GB		39%	Average % in GB		37%
		noise = $\frac{3}{4}$	# Y-Junctions	# Y-Junctions in GB	noise = $\frac{3}{4}$	# Y-Junctions	# Y-Junctions in GB	noise = $\frac{3}{4}$	# Y-Junctions	# Y-Junctions in GB
		906	209	56	906	209	52	906	209	48
		4047	224	85	4047	224	74	4047	224	69
		90525	219	79	90525	219	85	90525	219	86
		882232	222	64	882232	222	59	882232	222	57
		Average % in GB		32%	Average % in GB		31%	Average % in GB		30%
		noise = 1	# Y-Junctions	# Y-Junctions in GB	noise = 1	# Y-Junctions	# Y-Junctions in GB	noise = 1	# Y-Junctions	# Y-Junctions in GB
	14.0°	5062	209	66	5062	209	67	5062	209	65
		29490	216	66	29490	216	64	29490	216	65
		65719	219	94	65719	219	97	65719	219	89
		1275730	211	97	1275730	211	101	1275730	211	98
		Average % in GB		38%	Average % in GB		38%	Average % in GB		37%
		noise = $\frac{5}{4}$	# Y-Junctions	# Y-Junctions in GB	noise = $\frac{5}{4}$	# Y-Junctions	# Y-Junctions in GB	noise = $\frac{5}{4}$	# Y-Junctions	# Y-Junctions in GB
		55142	208	63	55142	208	65	55142	208	54
		151274	215	65	151274	215	65	151274	215	63
		569271	212	60	569271	212	50	569271	212	49
		1006568	207	33	1006568	207	32	1006568	207	35
		Average % in GB		26%	Average % in GB		25%	Average % in GB		24%

For all simulations in the below table, $R = -\frac{1}{10}; \psi_0 = -\frac{\sqrt{-R}}{2}$

Domain Rotation Cutoff		Box Dimension (in units of row width)								
		≈3			≈4			≈5		
	10.0°	noise = $\frac{3}{4}$	# Y-Junctions	# Y-Junctions in GB	noise = $\frac{3}{4}$	# Y-Junctions	# Y-Junctions in GB	noise = $\frac{3}{4}$	# Y-Junctions	# Y-Junctions in GB
		41329	206	176	41329	206	172	41329	206	172
		260407	211	160	260407	211	157	260407	211	154
		797443	225	171	797443	225	178	797443	225	177
		56228384	218	162	56228384	218	161	56228384	218	160
		Average % in GB		78%	Average % in GB		78%	Average % in GB		77%
		noise = 1	# Y-Junctions	# Y-Junctions in GB	noise = 1	# Y-Junctions	# Y-Junctions in GB	noise = 1	# Y-Junctions	# Y-Junctions in GB
		87316	210	175	87316	210	158	87316	210	162
		316054	206	171	316054	206	164	316054	206	149
		3557464	205	173	3557464	205	170	3557464	205	167
		5098315	219	167	5098315	219	159	5098315	219	154
		Average % in GB		82%	Average % in GB		78%	Average % in GB		75%
		noise = $\frac{5}{4}$	# Y-Junctions	# Y-Junctions in GB	noise = $\frac{5}{4}$	# Y-Junctions	# Y-Junctions in GB	noise = $\frac{5}{4}$	# Y-Junctions	# Y-Junctions in GB
		1106	227	191	1106	227	196	1106	227	193
		118899	199	166	118899	199	170	118899	199	170
		292476	197	157	292476	197	155	292476	197	154
		1107199	198	159	1107199	198	155	1107199	198	158
		Average % in GB		82%	Average % in GB		82%	Average % in GB		82%
	12.0°	noise = $\frac{3}{4}$	# Y-Junctions	# Y-Junctions in GB	noise = $\frac{3}{4}$	# Y-Junctions	# Y-Junctions in GB	noise = $\frac{3}{4}$	# Y-Junctions	# Y-Junctions in GB
		41329	206	159	41329	206	156	41329	206	153
		260407	211	137	260407	211	136	260407	211	138
		797443	225	154	797443	225	163	797443	225	158
		56228384	218	124	56228384	218	129	56228384	218	131
		Average % in GB		67%	Average % in GB		68%	Average % in GB		67%
		noise = 1	# Y-Junctions	# Y-Junctions in GB	noise = 1	# Y-Junctions	# Y-Junctions in GB	noise = 1	# Y-Junctions	# Y-Junctions in GB

Angle									
	87316	210	122	87316	210	127	87316	210	130
	316054	206	139	316054	206	130	316054	206	130
	3557464	205	154	3557464	205	150	3557464	205	149
	5098315	219	128	5098315	219	125	5098315	219	124
	Average % in GB		65%	Average % in GB		63%	Average % in GB		63%
	noise = $\frac{5}{4}$	# Y-Junctions	# Y-Junctions in GB	noise = $\frac{5}{4}$	# Y-Junctions	# Y-Junctions in GB	noise = $\frac{5}{4}$	# Y-Junctions	# Y-Junctions in GB
	1106	227	172	1106	227	174	1106	227	164
	118899	199	140	118899	199	149	118899	199	149
	292476	197	118	292476	197	127	292476	197	118
	1107199	198	133	1107199	198	135	1107199	198	136
	Average % in GB		69%	Average % in GB		71%	Average % in GB		69%
	noise = $\frac{3}{4}$	# Y-Junctions	# Y-Junctions in GB	noise = $\frac{3}{4}$	# Y-Junctions	# Y-Junctions in GB	noise = $\frac{3}{4}$	# Y-Junctions	# Y-Junctions in GB
	41329	206	121	41329	206	121	41329	206	120
	260407	211	105	260407	206	114	260407	206	116
	797443	225	133	797443	225	125	797443	225	124
	56228384	218	107	56228384	218	111	56228384	218	109
	Average % in GB		54%	Average % in GB		55%	Average % in GB		55%
	noise = 1	# Y-Junctions	# Y-Junctions in GB	noise = 1	# Y-Junctions	# Y-Junctions in GB	noise = 1	# Y-Junctions	# Y-Junctions in GB
14.0°	87316	210	95	87316	210	101	87316	210	98
	316054	206	104	316054	206	97	316054	206	88
	3557464	205	120	3557464	205	121	3557464	205	119
	5098315	219	98	5098315	219	97	5098315	219	92
	Average % in GB		50%	Average % in GB		50%	Average % in GB		47%
	noise = $\frac{5}{4}$	# Y-Junctions	# Y-Junctions in GB	noise = $\frac{5}{4}$	# Y-Junctions	# Y-Junctions in GB	noise = $\frac{5}{4}$	# Y-Junctions	# Y-Junctions in GB
	1106	227	135	1106	227	133	1106	227	123
	118899	199	114	118899	199	117	118899	199	121
	292476	197	85	292476	197	87	292476	197	90
	1107199	198	106	1107199	198	116	1107199	198	119
	Average % in GB		54%	Average % in GB		55%	Average % in GB		55%
