# Supplementary material for: Defect patterns on the curved surface of fish retinae suggest a mechanism of cone mosaic formation
Source: PLoS Comput Biol. 2020 Dec 15;16(12):e1008437. doi: 10.1371/journal.pcbi.1008437 (PMC7771878; doi:10.1371/journal.pcbi.1008437)
Supplement: S6 Table — See Alternative method for detection of grain boundaries for a description of the alternative method for grain boundary detection. These retinae correspond to the same retinae in Table 1. We show the percentage of retinal area analyzed, the number of Y-Junctions identified, and the percentage of Y-Junctions in grain boundaries according to the alternative method (based on linear alignment of Y-Junctions). (PDF) [file pcbi.1008437.s018.pdf]

Fish #	Retinal Area ($10^6\ \mu m^2$)	% Area Analyzed	Y-Junction Count	% of Y-Junctions in GB
1	2.4	47	155	54
2	2.1	46	166	42
3	3.9	57	221	48
4	3.8	70	275	48
5	5.3	66	249	55
6	4.2	61	184	36
7	5.4	51	182	42
8	5.4	63	285	51
